# Mirtazapine for Methamphetamine Use Disorder: A Randomized Clinical Trial

**DOI:** 10.1001/jamapsychiatry.2026.0159

**Published:** 2026-04-01

**Authors:** Rebecca McKetin, Steven Shoptaw, Lucy Saunders, Long Nguyen, Philip J. Clare, Gregory J. Dore, Alyna Turner, Olivia M. Dean, Peter J. Kelly, Shalini Arunogiri, Juanita Koeijers, Tayla J. Degan, Louisa Degenhardt, Michael Farrell, David Goodman-Meza, Barbara Sinclair, David Reid, Frank Cordaro, Harry Hill, Robert Lundin, Jeremy Hayllar, Michael Christmass, Willy Liaw, Danica Liu, Amelia Woods, Blaire Brewerton, Ellie Holyoak, Brian Tid-Fung Wu, Hayley Maher, Noni O’Dea, Joel Keygan, Ava Kontogiannis, Lily Palmer, Caity Morrison, Anna Wrobel, Bec Hyland, Gift Kiden, Vanessa Romeo, Khine Wut Yee Kyaw, Marianne Byrne, Samantha Colledge-Frisby, Emma Zahra, Michael Berk

**Affiliations:** 1 National Drug and Alcohol Research Centre, University of New South Wales, Sydney, New South Wales, Australia; 2 Department of Family Medicine, David Geffen School of Medicine, Los Angeles, California; 3 Burnet Institute, Melbourne, Victoria, Australia; 4 Prevention Research Collaboration, Sydney School of Public Health, University of Sydney, Sydney, New South Wales, Australia; 5 Charles Perkins Centre, University of Sydney, Sydney, New South Wales, Australia; 6 Kirby Institute, University of New South Wales, Sydney, New South Wales, Australia; 7 The Institute for Mental and Physical Health and Clinical Translation, School of Medicine, Deakin University, Geelong, Victoria, Australia; 8 Florey Institute for Neuroscience and Mental Health, University of Melbourne, Melbourne, Victoria, Australia; 9 School of Psychology, Faculty of Arts, Social Sciences and Humanities, University of Wollongong, Wollongong, New South Wales, Australia; 10 Monash Addiction Research Centre, Eastern Health Clinical School, Monash University, Melbourne, Victoria, Australia; 11 Drug and Alcohol Service, Illawarra Shoalhaven Local Health District, Wollongong, New South Wales, Australia; 12 Drug and Alcohol Services, Barwon Health, Geelong, Victoria, Australia; 13 Biala, Metro North Health, Brisbane, Queensland, Australia; 14 Next Step Community Alcohol and Drug Service, Perth, Western Australia, Australia; 15 Drug and Alcohol Services of South Australia, Adelaide, South Australia, Australia; 16 Alcohol, Tobacco and Other Drugs Service, Townsville, Queensland, Australia; 17 National Drug Research Institute, Curtin University, Perth, Western Australia, Australia

## Abstract

**Importance:**

Methamphetamine use disorder is a global health challenge for which there are no approved pharmacotherapies. The safety and effectiveness of mirtazapine, a promising candidate for methamphetamine use disorder, has not been established in routine clinical practice.

**Objective:**

To determine the safety and effectiveness of mirtazapine as a pharmacotherapy for methamphetamine use disorder in routine clinical practice.

**Design, Setting, and Participants:**

This phase 3, parallel-group, double-blind, placebo-controlled randomized clinical trial was conducted between November 16, 2022, and May 1, 2025, at 6 outpatient alcohol and other drug clinics in Australia among adults with moderate to severe methamphetamine use disorder. Data analysis was conducted from May to September 2025.

**Intervention:**

Mirtazapine (30 mg daily for 12 weeks) or equivalent placebo.

**Main Outcomes and Measures:**

The primary end point was the change in days of methamphetamine use in the past 28 days from baseline to week 12. Secondary end points were depression, insomnia, HIV risk behavior, quality of life, and methamphetamine-negative oral fluid samples.

**Results:**

Of 344 participants randomized, 339 participants received the intervention (167 in the placebo group and 172 in the mirtazapine group). Mean (SD) age was 42.0 (8.6) years, 126 participants (37.2%) were female, and participants had used methamphetamine for a median (IQR) of 24 days (17-28) of the past 28 days at baseline. The mean reduction in days of methamphetamine use from baseline to week 12 was greater in the mirtazapine group (7.0 days of 28 days) than in the placebo group (4.8 days of 28 days; mean difference, 2.2 days; 95% CI, −4.2 to −0.2 days; *P* = .02). More participants in the mirtazapine group reported drowsiness (47% vs 33%) and weight gain (10% vs 3%). Forty participants (23%) discontinued mirtazapine due to adverse events compared to 25 participants (15%) in the placebo group. No significant effects of mirtazapine on secondary end points were found.

**Conclusions and Relevance:**

In this parallel-group randomized clinical trial, mirtazapine delivered in routine clinical practice reduced methamphetamine use in adults with methamphetamine use disorder. No unexpected safety concerns delivering mirtazapine in this setting were found; this finding has important clinical implications in the absence of any approved pharmacotherapies for methamphetamine use disorder.

**Trial Registration:**

anzctr.org.au Identifier: ACTRN12622000235707

## Key Points

**Question:**

Is mirtazapine safe and effective for methamphetamine use disorder when delivered in routine clinical practice?

**Findings:**

In this double-blind, placebo-controlled randomized clinical trial of 344 adults with methamphetamine use disorder, 12 weeks of mirtazapine (30 mg/day) delivered in routine clinical practice produced a greater reduction in methamphetamine use days than placebo. There were no unexpected safety concerns from mirtazapine.

**Meaning:**

Mirtazapine is safe and effective when used in routine clinical practice for reducing methamphetamine use in adults with methamphetamine use disorder.

## Teaser

This randomized clinical trial conducted in Australia determines the safety and effectiveness of mirtazapine as a pharmacotherapy for methamphetamine use disorder in routine clinical practice.

## Introduction

Methamphetamine is a highly addictive synthetic stimulant drug that is a growing global public health concern.^1^ An estimated 7.4 million people worldwide have a methamphetamine use disorder, which is associated with an elevated risk of psychosis, cardiovascular events, accidental injuries, suicide, homicide, and suboptimal neonatal outcomes.^1^ In the US, methamphetamine use is a leading cause of drug-related death.^2,3,4^ Globally, it accounts for an estimated excess of 326 000 deaths per year (95% uncertainty interval, 228 000-449 000).^1^ No medications are approved by the US Food and Drug Administration for methamphetamine use disorder, and only a few options have provided hope.^1,5,6^

The generic tetracyclic antidepressant mirtazapine is a promising candidate.^7^ Mirtazapine modulates dopamine function via its affinity for serotonin (5-hydroxytryptamine [5-HT]) 5-HT_2A_, 5-HT_2C_, and 5-HT_3_ receptors, and this is thought to mediate its ability to reduce methamphetamine’s effects^8,9^ and ameliorate the dopaminergic dysregulation seen in methamphetamine addiction.^9^ Mirtazapine also blocks central presynaptic α2-adrenergic receptors, which are dysregulated in methamphetamine addiction.^10^ Its antagonism of histamine-1 receptors^9^ benefits insomnia and anxiety, which often feature in methamphetamine use disorder.^11^

Evidence supporting the use of mirtazapine for methamphetamine use derives from 2 single-site phase 2 trials, the first with 60 participants and the second with 120.^12,13^ Together, these studies showed a 14% difference in active vs placebo condition in methamphetamine-positive urine tests.^7^ These benefits were apparent after 12 weeks of mirtazapine treatment.^12,13^ The second larger trial by Coffin and colleagues^13^ provided 24 weeks of mirtazapine treatment; the treatment effect of mirtazapine on methamphetamine use was similar at 12 and 24 weeks. Coffin and colleagues^13^ also found reductions in depressive symptoms and insomnia. Both trials were conducted in a clinical trial setting specifically in nondepressed men or transgender women who were having sex with men.

Based on consistent results from these trials, a multisite phase 3 randomized clinical trial was conducted to establish the effectiveness and safety of mirtazapine in a more diverse population of people with methamphetamine use disorder in routine clinical practice.

## Methods

### Trial Design

We conducted a phase 3, investigator-led, double-blind, placebo-controlled, parallel-arm randomized clinical trial at 6 outpatient government-run alcohol and other drug clinics in Australia. These clinics were located in Wollongong, Geelong, Townsville, Perth, Brisbane, and Adelaide. This trial was coordinated by the University of New South Wales, which was also the sponsor. The protocol^14^ was approved by an independent ethics committee and prospectively registered with the Australian and New Zealand Clinical Trials Registry (ACTRN12622000235707). The trial protocol is available in Supplement 1 and the statistical analysis plan in Supplement 2. The trial was conducted in accordance with the principles of the Declaration of Helsinki and with the Good Clinical Practice guidelines of the International Council for Harmonization. All participants provided informed written consent prior to participation. All clinicians, researchers, and investigators were blinded to condition allocation. An independent data safety and monitoring board oversaw the study.

### Participants

Participants were eligible if they were aged 18 to 65 years, had a moderate or severe methamphetamine use disorder in the past year according to the *DSM-5*,^15^ had used methamphetamine at least twice weekly in the past 4 weeks, and had a positive drug screen result for amphetamines. Participants were ineligible if they were taking prescribed antidepressant medication, had attempted suicide in the past year, were pregnant or lactating, needed acute medical care, were undergoing inpatient treatment, were incarcerated, or had contraindications for using mirtazapine. Detailed criteria are provided in the eMethods in Supplement 3.

### Procedures

Participants were recruited primarily via social media, flyers at health services, and word of mouth. Participants were phone screened and eligibility was confirmed at a face-to-face assessment at the trial site clinic, where participants were enrolled as patients. Participants were randomized 1:1 to receive either mirtazapine (30 mg/day for 12 weeks) or a matching placebo tablet. Placebo tablets were supplied by Syntro Pty Ltd. Randomization was stratified on sex (male vs female or other sex at birth) and depression (Patient Health Questionnaire–9 [PHQ-9] score ≥10). Participants were directed to take the trial medication orally each evening. The trial medication was provided to participants in bottles of 35 tablets, each fitted with a MEMS Cap (Aardex Group) to monitor medication adherence. The first medication bottle was provided at baseline. Medication bottles were replaced at weeks 4 and 8. Participants were provided with a taper dose (15 mg for 28 days) from week 12. All participants were provided with referral information and a drug use harm reduction brochure. Participants were free to access other available drug treatment and health services throughout the trial.

Clinicians could temporarily or permanently discontinue the trial medication in response to suspected adverse reactions. Participants could also choose to discontinue the trial medication for any reason. Clinicians were able to offer participants who discontinued the trial medication for tolerability reasons a 15-mg rescue dose (ie, taking half of the scored 30-mg trial medication tablet) to facilitate trial retention. Participants who discontinued the trial medication were encouraged to remain in the trial to complete outcome assessments.

Assessments of outcomes were at baseline, week 4, week 8, and week 12. An additional adverse event review was done by phone at week 2. Assessments were conducted by a trained researcher either in person or by phone. Data were collected and managed using an electronic data capture platform (REDCap)^16^ hosted by the University of New South Wales. Participants were reimbursed 50 Australian dollars (US $35.59) for each assessment.

### End Points

The primary outcome was the change in self-reported days of methamphetamine use in the past 28 days, assessed using the timeline follow-back (TLFB) method,^17^ from baseline to weeks 4, 8, and 12, with week 12 being the primary end point. The TLFB is a validated measure of stimulant use that shows 88% sensitivity, 96% specificity, and a 95% concordance against amphetamine urine test results.^17^ In this trial, sensitivity was 89% and specificity was 83% for the past 3 days of self-reported methamphetamine use against methamphetamine-positive oral fluid.

Secondary outcomes were the change in depression, insomnia, HIV risk behavior, and quality of life from baseline to weeks 4, 8, and 12, with week 12 being the primary end point. Depression was assessed using the PHQ-9,^18^ insomnia with the Athens Insomnia Scale (5-item version),^19^ HIV risk behavior with the HIV Risk-Taking Behavior Scale from the Opioid Treatment Index,^20^ and quality of life was the utility score from the 5-level EuroQol-5D.^21^ Oral fluid samples were collected at weeks 4, 8, and 12, assayed for methamphetamine (≥25 ng/mL), and compared across all 3 time points.

Exploratory outcomes included suicide risk (score ≥3 on the Columbia Suicide Severity Rating Scale–Screener [CSSRS-S])^22,23^ at any time in the 12-week intervention period and other substance use (total days of use for other major drug classes [tobacco, alcohol, cannabis, cocaine, ecstasy, hallucinogens, inhalants, and heroin] during the past 28 days). Medication adherence was based on returned MEMS Caps and defined as the percentage of days during the 12-week intervention period when the medication bottle was opened. Other exploratory end points are described in the eMethods in Supplement 3.

Safety was assessed as the percentage of participants reporting adverse events by system organ classification, coded according to the Medical Dictionary for Regulatory Activities, version 27.0.^24^ Participant-reported adverse event data were collected at each assessment.

### Statistical Analysis

It was estimated a sample size of 340 participants would provide 90% power to detect a minimum rate ratio of 0.75 on the primary end point with a 2-sided significance level of .05. The nominated effect size was based on the previous trial conducted by Coffin and colleagues.^13^ We assumed 75% follow-up at week 12. We did not adjust the width of confidence intervals for multiple comparisons.

All treatment estimands were based on participants who received the trial medication (n = 339) and all available data from the baseline assessment to the week 12 assessment, regardless of whether participants discontinued the trial medication or received the 15-mg rescue dose. The treatment estimand for the primary end point of methamphetamine use days and the secondary end points of depression, insomnia, HIV risk, and quality of life were determined using a condition (placebo vs mirtazapine) by time (baseline, week 4, week 8, week 12) interaction effect, with week 12 being the primary end point. Mixed models with a random intercept for repeated assessments were used. Time was included as a factor variable, producing treatment estimands for each time point (weeks 4, 8, and 12) and making no assumptions about the linearity of changes over time. The average treatment estimand was based on a comparison of conditions across all follow-up time points (weeks 4, 8, and 12). A negative binomial model was used for our primary outcome of methamphetamine use days, yielding an incidence rate ratio for days of use. An additional analysis of the primary end point was adjusted for a prespecified set of covariates from the eligibility assessment (days of methamphetamine use, methamphetamine injection, PHQ-9 score, age, and sex). The analysis of the methamphetamine-negative oral fluid samples compared conditions (placebo vs mirtazapine) across all 3 follow-up time points (weeks 4, 8, and 12) to obtain an average treatment estimand.

Sensitivity analyses were conducted for the primary end point and the secondary outcome of methamphetamine-negative oral fluid tests, which imputed missing data using imputation by chained equations (see the eMethods for details and eTables 1 and 2 in Supplement 3). Subgroup analyses included an interaction effect for sex (male vs female at birth) and depression at eligibility (PHQ-9 score <10 vs ≥10, the latter corresponding to a diagnosis of major depression with 88% sensitivity and 88% specificity^18^), respectively. Means, 95% confidence limits, and *P* values for subgroup comparisons were derived from models using Stata’s postestimation command suite for nonlinear combinations of estimates in version 18.0 SE (StataCorp). All analyses were prespecified in the trial statistical analysis plan (Supplement 2), which contains further details on the statistical analysis.

## Results

### Participants

**Figure 1.**
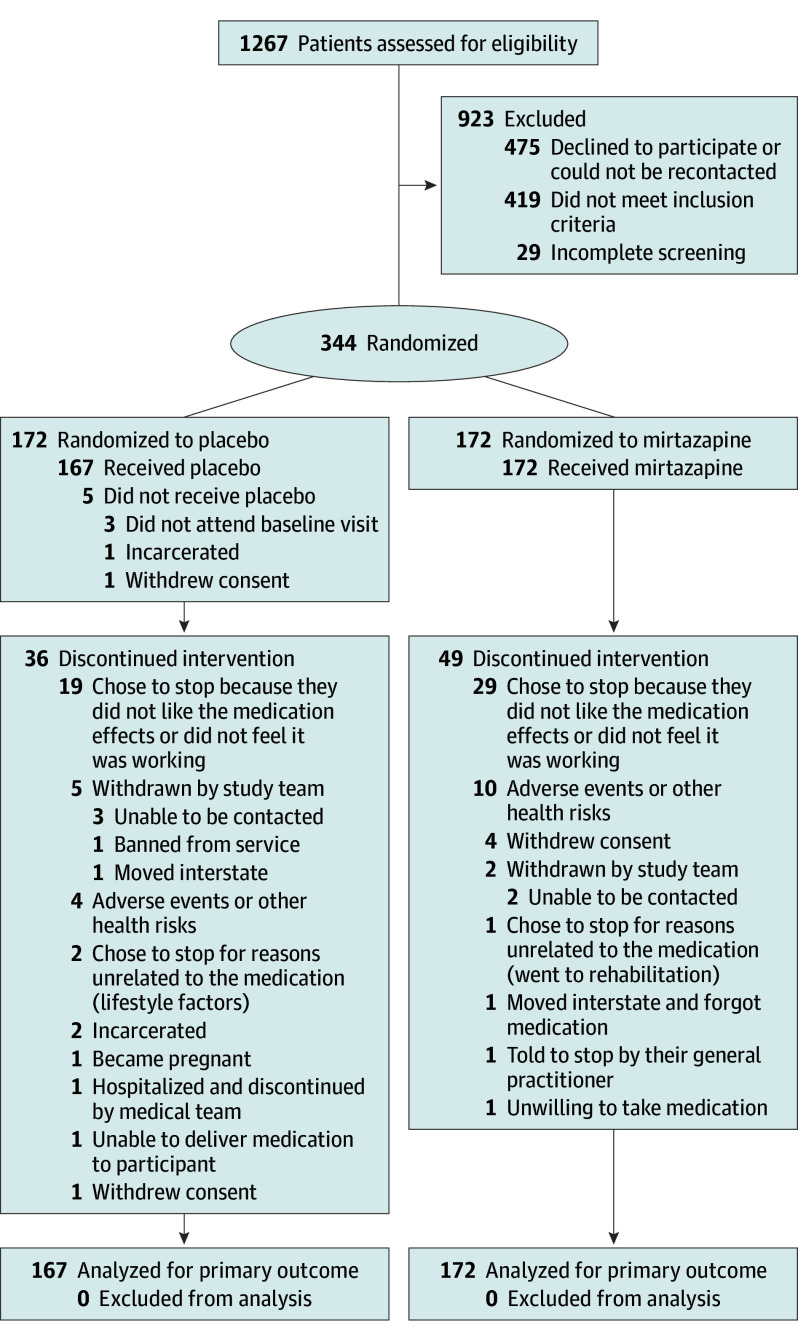
CONSORT Flow Diagram

The trial was conducted between November 16, 2022, and May 1, 2025. Of the 344 participants randomized, 339 attended the baseline assessment where they received the trial medication (Figure 1). Mean (SD) participant age was 42.0 (8.6) years, and 126 participants (37.2%) were female. Overall, 85 participants (25%) discontinued the trial medication, with 65 (19%) discontinuing for reasons related to the trial medication (23% in the mirtazapine group and 15% in the placebo group). Eight of these discontinued participants received the rescue dose (ie, 15 mg of the trial medication per day): 6 in the mirtazapine group and 2 in the placebo group. Median (IQR) medication adherence was 52% (26%-78%) overall and 62% (40%-81%) when excluding assessment periods when participants were discontinued from the trial medication. Week 12 assessment data were available for 293 of 339 participants (86%) (88% in the mirtazapine group and 85% in the placebo group).

Demographics and drug use were similar in the 2 groups (Table 1; additional demographics reported in eTable 3 in Supplement 3). A total of 179 participants (52.8%) were unemployed, and 136 (40%) had a prison history. The mean duration of methamphetamine use was 21 years. Participants used methamphetamine on a median (IQR) of 24 days (17-28) of the past 28 days at baseline.

**Table 1.** Demographic and Clinical Characteristics of the Participants at Baseline

Characteristic	No. (%)		*P* value
	Placebo (n = 167)	Mirtazapine (n = 172)	
Age, mean (SD), y	41.9 (8.3)	42.1 (8.9)	.79
Sex			
Female	61 (36.5)	65 (37.8)	.81
Male	106 (63.5)	107 (62.2)	
Born outside of Australia	27 (16.2)	28 (16.3)	.98
Schooling, median (IQR), y	10 (10-12)	10 (10-12)	.95
Employment			
Unemployed	90 (53.9)	89 (51.7)	.83
Full-time employment	39 (23.4)	39 (22.7)	
Other employment^a^	38 (22.8)	44 (25.6)	
Income in the past fortnight			
<A$800 (US $569.91)	64 (38.3)	57 (33.1)	.25
A$800-A$1199 (US $569.91-$854.16)	40 (24.0)	55 (32.0)	
≥A$1200 (US $854.87)	63 (37.7)	60 (34.9)	
Education			
No tertiary education	49 (29.3)	59 (34.3)	.59
Trade or technical qualification	106 (63.5)	100 (58.1)	
University degree	12 (7.2)	13 (7.6)	
Injecting methamphetamine	81 (48.5)	74 (43.0)	.31
Days of methamphetamine use in the past 4 wk, median (IQR)^b^	24 (16-28)	25 (18-28)	.30
Days of other substance use in the past 4 wk, mean (SD)	35.8 (20.5)	33.5 (19.2)	.30
Depression (PHQ-9 score), median (IQR)	9 (5-13)	8 (4-13)	.60
HIV risk (OTI HRBS score), median (IQR)	5 (2-8)	4 (2-7)	.79
Insomnia AIS-5 score, median (IQR)	3 (0-6)	3 (0-6)	.50
Quality of life (EQ-5D utility score), median (IQR)	0.92 (0.85-0.97)	0.92 (0.85-0.97)	.99
Depressed at eligibility (PHQ-9 score ≥10)	82 (49.1)	83 (48.3)	.88

Abbreviations: A$, Australian dollar; AIS-5, Athens Insomnia Scale, 5-item version; EQ-5D, EuroQol-5D; OTI HRBS, Opioid Treatment Index HIV Risk-Taking Behavior Scale; PHQ-9, Patient Health Questionnaire–9.

^a^ Casual or part-time employment, home duties, or student.

^b^ Censoring incarceration and hospitalization.

### Change in Methamphetamine Use

The mean days of methamphetamine use in the past 28 days at baseline was 22.0 (95% CI, 20.3-23.8) in the placebo group and 23.1 (95% CI, 21.3-24.9) in the mirtazapine group. Mirtazapine was associated with a greater reduction in days of methamphetamine use in the past 28 days from baseline to week 12 (−7.0 days; 95% CI, −8.5 to −5.6) than placebo (−4.8 days; 95% CI, −6.2 to −3.4), giving a treatment estimand of −2.2 days (95% CI, −4.2 to −0.2; *P* = .02) (Table 2; eTable 4 in Supplement 3; model details can be found in eTables 5 and 6 in Supplement 3). This treatment estimand represents an 8% reduction in the risk of methamphetamine use for a given day (or a reduction of 8 days of methamphetamine use of 100 possible use days; incidence rate ratio, 0.89; 95% CI, 0.80-0.98). The treatment estimands were smaller at week 4 (−1.3 days; 95% CI, −3.3 to 0.6; *P* = .20) and week 8 (−1.9 days; 95% CI, −3.9 to 0.1; *P* = .06) (eFigure 1 and eTable 5 in Supplement 3). The primary treatment estimand was robust to imputation of missing data (eTable 7 in Supplement 3) and adjusting for baseline covariates (eFigure 2, eTables 8 and 9 in Supplement 3). The mean treatment estimand across the 12-week intervention was a reduction of 1.8 days in the past 28 days (95% CI, −3.5 to −0.1; *P* = .03).

**Table 2.** Primary and Secondary End Points for the Intention-to-Treat Estimand

End point	Placebo (n = 167)	Mirtazapine (n = 172)	Treatment estimand (95% CI)	*P* value
Primary end point				
Mean change in days of methamphetamine use	−4.8	−7.0	−2.2 (−4.2 to −0.2)	.02
Secondary end points				
Methamphetamine-negative oral fluid samples, %	12.0	13.2	1.3 (−4.7 to 7.2)	.68
Mean change in PHQ-9 score	−2.3	−2.5	−0.2 (−1.3 to 1.0)	.77
Mean change in AIS-5 score	−1.2	−1.8	−0.6 (−1.4 to 0.2)	.13
Mean change in HIV risk behavior score	−1.1	−1.4	−0.3 (−1.0 to 0.3)	.32
Mean change in EQ-5D quality of life utility score	1.3	2.2	0.9 (−3.1 to 4.9)	.66

Abbreviations: AIS-5, Athens Insomnia Scale, 5-item version; EQ-5D, EuroQol-5D; PHQ-9, Patient Health Questionnaire–9.

### Secondary End Points

**Figure 2.**
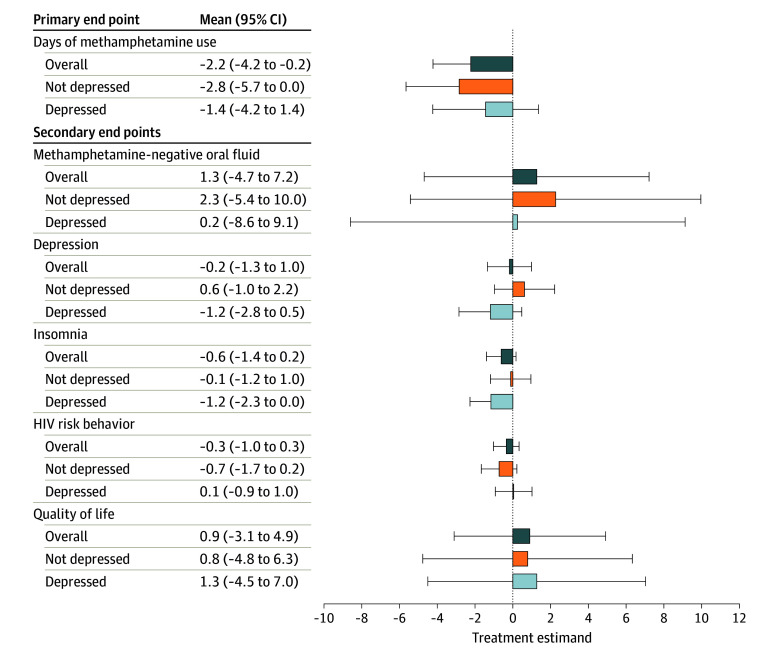
Forest Plot Showing Treatment Estimands by Whether Participants Were Depressed at Eligibility Bars represent the mean (95% CI) treatment estimand at week 12 for mirtazapine vs placebo by whether participants were not depressed (Patient Health Questionnaire–9 [PHQ-9] score <10) or depressed (PHQ-9 score ≥10) on entry to the trial. End points on the y-axis (from top to bottom): the number of days of methamphetamine use in the past 28 days assessed using the timeline follow-back method; the percentage of methamphetamine-negative oral fluid tests taken at weeks 4, 8, and 12; depression score on the PHQ-9; insomnia score on the Athens Insomnia Scale, 5-item version; HIV risk behavior score on the HIV Risk-Taking Behavior Scale of the Opioid Treatment Index; and quality of life utility score on the EuroQol-5D.

Treatment estimands on secondary end points did not differ significantly between conditions, but trends were in favor of a benefit of mirtazapine over placebo (Figure 2; eTable 4 in Supplement 3).

### Exploratory End Points

Eleven participants exceeded the suicide risk threshold on the CSSRS-S between baseline and week 12: 5 (3%) in the mirtazapine condition and 6 (4%) in the placebo condition (Table 3). The mirtazapine and placebo conditions did not differ in days of other substance use (eTable 2 in Supplement 3).

**Table 3.** Adverse Events and Safety During the 12 Weeks of Trial Medication

Adverse event	No. (%)			*P* value
	Placebo (n = 167)	Mirtazapine (n = 172)	Total (N = 339)	
All medication discontinuations^a^	36 (22)	49 (28)	85 (25)	.14
Medication discontinuation due to adverse reactions^a^	25 (15)	40 (23)	65 (19)	.19
Any adverse event	145 (87)	155 (90)	300 (89)	.34
Any serious adverse event	11 (7)	7 (4)	18 (5)	.30
Serious adverse events, No.	11	7	18	.30
Suicide risk^b^	6 (4)	5 (3)	11 (4)	.68
Adverse events by system organ class for adverse events occurring in ≥5% of participants				
Gastrointestinal disorders	26 (16)	22 (13)	48 (14)	.46
General disorders and administration site conditions	22 (13)	21 (12)	43 (13)	.79
Infections and infestations	33 (20)	32 (19)	65 (19)	.79
Injury, poisoning, and procedural complications	21 (13)	24 (14)	45 (13)	.71
Investigations	8 (5)	19 (11)	27 (8)	.03
Metabolism and nutrition disorders	48 (29)	55 (32)	103 (30)	.52
Musculoskeletal and connective tissue disorders	17 (10)	23 (13)	40 (12)	.36
Nervous system disorders	78 (47)	111 (65)	189 (56)	.001
Psychiatric disorders	74 (44)	85 (49)	159 (47)	.35
Respiratory, thoracic, and mediastinal disorders	23 (14)	26 (15)	49 (14)	.73
Skin and subcutaneous tissue disorders	15 (9)	15 (9)	30 (9)	.93
Adverse events occurring in ≥5% of participants^c^				
Drowsiness	55 (33)	80 (47)	135 (40)	.01
Increased appetite	45 (27)	48 (28)	93 (27)	.84
Weight gain	5 (3)	17 (10)	22 (6)	.01
Low mood	20 (12)	24 (14)	44 (13)	.59
Headache	23 (14)	21 (12)	44 (13)	.67
Vivid dreams	16 (10)	21 (12)	37 (11)	.44
Cold	14 (8)	15 (9)	29 (9)	.91
Irritable	9 (5)	13 (8)	22 (6)	.42
Suicidal ideation	11 (7)	11 (6)	22 (6)	.94

^a^ Excludes temporary discontinuation from the trial medication.

^b^ Score of ≥3 on the Columbia Suicide Severity Rating Scale–Screener.

^c^ By preferred Medical Dictionary for Regulatory Activities term.

### Subgroup Analysis

Treatment estimands for days of methamphetamine use and secondary end points were similar for male and female participants (eTables 10 and 11 in Supplement 3) and by whether participants were depressed (eTables 12 and 13 in Supplement 3), although the point estimands for insomnia and depression were larger for participants who were depressed (Figure 2), with these being statistically significant for insomnia. Among participants who were depressed at baseline, the treatment estimands for insomnia were −1.8 (95% CI, −2.9 to −0.8; *P* = .001) at week 4, −1.7 (95% CI, −2.8 to −0.6; *P* = .002) at week 8, and −1.2 (95% CI, −2.3 to 0.0; *P* = .04) at week 12; for depression, treatment estimands were −0.7 (95% CI, −2.3 to 0.9; *P* = .41) at week 4, −1.3 (95% CI, −3.0 to 0.3; *P* = .11) at week 8, and −1.2 (95% CI, −2.8 to 0.5; *P* = .16) at week 12.

### Safety

More participants in the mirtazapine group reported adverse events for system organ classes of nervous system disorders and investigations, these being related to excess drowsiness and reported weight gain, respectively (Table 3). Full details of adverse events that occurred or worsened from baseline to week 12 are provided in eTables 14 and 15 in Supplement 3. There were 18 serious adverse events during this period, with 7 in the mirtazapine group. None were deemed to be related to the trial medication. Details can be found in eTable 16 in Supplement 3.

## Discussion

In this randomized clinical trial, people with methamphetamine use disorder had a mean reduction of 7 days (out of 28 days) after 12 weeks of mirtazapine (30 mg/day) treatment, which was approximately 2 days more than the reduction seen with placebo. In this trial, we confirm the preliminary observed benefits of mirtazapine from phase 2 trials and generalize these to routine clinical practice and a broader population of people with methamphetamine use disorder. We did not find any unexpected safety concerns associated with prescribing mirtazapine to people with methamphetamine use disorder, although participants receiving mirtazapine reported more drowsiness (47% vs 33%) and weight gain (10% vs 3%).

The clinical significance of these findings lies in identifying a safe and cheap generic medication that can be prescribed to help people reduce methamphetamine use. The well-established safety profile of mirtazapine means it can be easily and safely prescribed in an outpatient setting with limited clinical oversight. Other pharmacotherapy agents under investigation (eg, high-dose prescription stimulant medications,^25^ long-acting opioid antagonists^6^) are usually prescribed by addiction medicine specialists and require close clinical supervision to manage potential risks (eg, overdose, toxicity, and abuse liability). The potential of mirtazapine for broader application addresses questions of accessibility and scalability inherent to other agents under investigation.

The impact of mirtazapine on methamphetamine use in routine clinical practice appeared diluted compared to the previous phase 2 trials.^7^ We found an 8% reduction in the risk of using methamphetamine on a given day compared to a 14% reduction in the risk of a methamphetamine-positive urine test in the previous phase 2 trials.^7^ However, in the absence of an approved pharmacotherapy, any leverage on improving clinical outcomes for methamphetamine use disorder is critical. Reductions in days of methamphetamine use impacts positively on functional outcomes^26^ and reduces the risk of methamphetamine-related psychotic symptoms^27^ and violent behavior.^28^ We also found preliminary evidence of benefits for insomnia among people with co-occurring depression, supporting the findings of the previous phase 2 trial by Coffin and colleagues.^13^

Consistent with the previous phase 2 trials of mirtazapine on methamphetamine use disorder,^12,13^ we found that reductions in methamphetamine use were not contingent on improvements in depression or insomnia. This finding implies that mirtazapine has a direct effect on addictive processes, consistent with animal models of addiction^9^ and human preclinical research.^8^ The neural mechanism behind this interaction is not known; however, mirtazapine has a high antagonism affinity for 5-HT_2A_ receptors, and antagonizing these receptors has been found to attenuate the rewarding properties of methamphetamine.^29,30^ Mirtazapine also enhances monoamine signaling, and this may help correct the downregulation of dopamine function seen following chronic methamphetamine use.^9^

### Strengths and Limitations

The strengths of this study include low study attrition and a generalizable target population that included essential subgroups who have been excluded from previous trials of mirtazapine for methamphetamine use, namely women (37% of our sample) and people who are depressed (49% of our sample). Rates of depression are similarly high among people who use methamphetamine in treatment settings^31^ and in the general community.^32^ Our measure of methamphetamine use days is a sensitive and clinically meaningful indicator of methamphetamine use that concords with functional outcomes.^26,33^ Self-reported substance use in research shows strong agreement with biological measures of substance use,^17,34,35^ including in our study. Treatment effects in this study may have been diluted by having a heterogeneous group of participants who had entrenched patterns of methamphetamine use, poor medication adherence, and high discontinuation rates. Greater adherence in highly motivated patients may yield more substantial benefits. However, our treatment effects are likely to be indicative of what can be expected in routine clinical practice.

## Conclusions

In summary, the results of this randomized clinical trial confirm that mirtazapine can be used in routine clinical practice to facilitate a reduction in methamphetamine use among people with a moderate to severe methamphetamine use disorder. There were no unexpected adverse reactions to mirtazapine that would compromise mirtazapine as a take-home medication in this population.

## Notes

Supplement 1. Trial Protocol

Supplement 2. Statistical Analysis Plan

Supplement 3. eMethods. Supplemental Methods eFigure 1. Estimated Mean Days of Methamphetamine Use by Condition From the ITT Model eFigure 2. Estimated Mean Days of Methamphetamine Use by Condition From the ITT Model Adjusted for Baseline Covariates eFigure 3. Estimated Means by Whether Participants Were Depressed for (A) Methamphetamine Use Days, (B) Depression, and (C) Insomnia eTable 1. Summary of Missing Data in Each Analysis and Auxiliary Variable eTable 2. Baseline Correlates of the Number of Missed Follow-Up Assessments (Weeks 4, 8, and 12) eTable 3. Participant Characteristics at Eligibility by Condition eTable 4. Primary, Secondary, and Tertiary End Points for the 12-Week Intention-to-Treat Estimand eTable 5. Model Output for the Primary End Point for the Intention-to-Treat Analysis eTable 6. Estimated Mean (95% CI) Days of Methamphetamine Use Extracted From the Intention-to-Treat Analysis eTable 7. Model Output for the Primary End Point for the Intention-to-Treat Analysis With Imputed Missing Data eTable 8. Model Output for the Primary End Point for the Intention-to-Treat Analysis With Adjustment for Baseline Covariates eTable 9. Estimated Mean (95% CI) Days of Methamphetamine Use Extracted From the Intention-to-Treat Analysis Adjusted for Baseline Covariates eTable 10. Primary and Secondary End Points for the Intention-to-Treat Estimand for Participants Who Were Male at Birth eTable 11. Primary and Secondary End Points for the Intention-to-Treat Estimand for Participants Who Were Female or Other at Birth eTable 12. Primary and Secondary End Points for the Intention-to-Treat Estimand for Participants Who Were Not Depressed at Eligibility eTable 13. Primary and Secondary End Points for the Intention-to-Treat Estimand for Participants Who Were Depressed at Baseline eTable 14. Adverse Events Within Each System Organ Classification (SOC) by Condition (From Baseline to Week 12) eTable 15. Adverse Events by MedDRA Diagnosis by Condition eTable 16. Serious Adverse Events by Condition eReferences.

Supplement 4. Data Sharing Statement

## References

[1] Farrell M, Martin NK, Stockings E, . Responding to global stimulant use: challenges and opportunities. Lancet. 2019;394(10209):1652-1667. doi:10.1016/S0140-6736(19)32230-5 31668409 PMC6924572

[2] Han B, Compton WM, Jones CM, Einstein EB, Volkow ND. Methamphetamine use, methamphetamine use disorder, and associated overdose deaths among US adults. JAMA Psychiatry. 2021;78(12):1329-1342. doi:10.1001/jamapsychiatry.2021.2588 34550301 PMC8459304

[3] Jones CM, Houry D, Han B, Baldwin G, Vivolo-Kantor A, Compton WM. Methamphetamine use in the United States: epidemiological update and implications for prevention, treatment, and harm reduction. Ann N Y Acad Sci. 2022;1508(1):3-22. doi:10.1111/nyas.14688 34561865 PMC9097961

[4] Anderer S. More than half of US overdose deaths now involve stimulants. JAMA. 2025;334(15):1321. doi:10.1001/jama.2025.13881 40971161

[5] Chan B, Freeman M, Kondo K, . Pharmacotherapy for methamphetamine/amphetamine use disorder-a systematic review and meta-analysis. Addiction. 2019;114(12):2122-2136. doi:10.1111/add.14755 31328345

[6] Trivedi MH, Walker R, Ling W, . Bupropion and naltrexone in methamphetamine use disorder. N Engl J Med. 2021;384(2):140-153. doi:10.1056/NEJMoa2020214 33497547 PMC8111570

[7] Naji L, Dennis B, Rosic T, . Mirtazapine for the treatment of amphetamine and methamphetamine use disorder: a systematic review and meta-analysis. Drug Alcohol Depend. 2022;232:109295. doi:10.1016/j.drugalcdep.2022.109295 35066460

[8] Rush CR, Santos GM, McMahan VM, . Mirtazapine reduces hypothetical methamphetamine demand in humans. Drug Alcohol Depend. 2025;274:112769. doi:10.1016/j.drugalcdep.2025.112769 40580890 PMC12279006

[9] Graves SM, Rafeyan R, Watts J, Napier TC. Mirtazapine, and mirtazapine-like compounds as possible pharmacotherapy for substance abuse disorders: evidence from the bench and the bedside. Pharmacol Ther. 2012;136(3):343-353. doi:10.1016/j.pharmthera.2012.08.013 22960395 PMC3483434

[10] Nishio M, Kanda Y, Mizuno K, Watanabe Y. Methamphetamine increases the hippocampal α(2A)-adrenergic receptor and Galpha(o) in mice. Neurosci Lett. 2002;334(3):145-148. doi:10.1016/S0304-3940(02)01033-9 12453616

[11] Vrajová M, Šlamberová R, Hoschl C, Ovsepian SV. Methamphetamine and sleep impairments: neurobehavioral correlates and molecular mechanisms. Sleep. 2021;44(6):zsab001. doi:10.1093/sleep/zsab001 33406259

[12] Colfax GN, Santos GM, Das M, . Mirtazapine to reduce methamphetamine use: a randomized controlled trial. Arch Gen Psychiatry. 2011;68(11):1168-1175. doi:10.1001/archgenpsychiatry.2011.124 22065532 PMC3437988

[13] Coffin PO, Santos GM, Hern J, . Effects of mirtazapine for methamphetamine use disorder among cisgender men and transgender women who have sex with men: a placebo-controlled randomized clinical trial. JAMA Psychiatry. 2020;77(3):246-255. doi:10.1001/jamapsychiatry.2019.3655 31825466 PMC6990973

[14] McKetin R, Degan TJ, Saunders L, . A phase 3 randomised double-blind placebo-controlled trial of mirtazapine as a pharmacotherapy for methamphetamine use disorder: a study protocol for the Tina Trial. Trials. 2024;25(1):408. doi:10.1186/s13063-024-08238-y 38907288 PMC11193254

[15] American Psychiatric Association. Diagnostic and Statistical Manual of Mental Disorders. 5th ed. American Psychiatric Association; 2013.

[16] Harris PA, Taylor R, Minor BL, ; REDCap Consortium. The REDCap consortium: building an international community of software platform partners. J Biomed Inform. 2019;95:103208. doi:10.1016/j.jbi.2019.103208 31078660 PMC7254481

[17] Fals-Stewart W, O’Farrell TJ, Freitas TT, McFarlin SK, Rutigliano P. The timeline followback reports of psychoactive substance use by drug-abusing patients: psychometric properties. J Consult Clin Psychol. 2000;68(1):134-144. doi:10.1037/0022-006X.68.1.134 10710848

[18] Kroenke K, Spitzer RL, Williams JB. The PHQ-9: validity of a brief depression severity measure. J Gen Intern Med. 2001;16(9):606-613. doi:10.1046/j.1525-1497.2001.016009606.x 11556941 PMC1495268

[19] Soldatos CR, Dikeos DG, Paparrigopoulos TJ. Athens Insomnia Scale: validation of an instrument based on ICD-10 criteria. J Psychosom Res. 2000;48(6):555-560. doi:10.1016/S0022-3999(00)00095-7 11033374

[20] Darke S, Hall W, Wodak A, Heather N, Ward J. Development and validation of a multi-dimensional instrument for assessing outcome of treatment among opiate users: the Opiate Treatment Index. Br J Addict. 1992;87(5):733-742. doi:10.1111/j.1360-0443.1992.tb02719.x 1591524

[21] Herdman M, Gudex C, Lloyd A, . Development and preliminary testing of the new five-level version of EQ-5D (EQ-5D-5L). Qual Life Res. 2011;20(10):1727-1736. doi:10.1007/s11136-011-9903-x 21479777 PMC3220807

[22] Posner K, Brent D, Lucas C, . Columbia-Suicide Severity Rating Scale (C-SSRS) Version 6*. *The Research Foundation for Mental Hygeine; 2008.

[23] Bjureberg J, Dahlin M, Carlborg A, Edberg H, Haglund A, Runeson B. Columbia-Suicide Severity Rating Scale screen version: initial screening for suicide risk in a psychiatric emergency department. Psychol Med. 2021;52(16):1-9. 33766155 10.1017/S0033291721000751 PMC9811343

[24] Brown EG, Wood L, Wood S. The Medical Dictionary for Regulatory Activities (MedDRA). Drug Saf. 1999;20(2):109-117. doi:10.2165/00002018-199920020-00002 10082069

[25] Ezard N, Clifford B, Siefried KJ, ; LiMA Investigator Group. Lisdexamfetamine in the treatment of methamphetamine dependence: a randomised, placebo-controlled trial. Addiction. 2025;120(7):1345-1359. doi:10.1111/add.16730 39701142 PMC12128569

[26] Amin-Esmaeili M, Farokhnia M, Susukida R, . Reduced drug use as an alternative valid outcome in individuals with stimulant use disorders: findings from 13 multisite randomized clinical trials. Addiction. 2024;119(5):833-843. doi:10.1111/add.16409 38197836 PMC11009085

[27] McKetin R, Lubman DI, Baker AL, Dawe S, Ali RL. Dose-related psychotic symptoms in chronic methamphetamine users: evidence from a prospective longitudinal study. JAMA Psychiatry. 2013;70(3):319-324. doi:10.1001/jamapsychiatry.2013.283 23303471

[28] McKetin R, Lubman DI, Najman JM, Dawe S, Butterworth P, Baker AL. Does methamphetamine use increase violent behaviour? evidence from a prospective longitudinal study. Addiction. 2014;109(5):798-806. doi:10.1111/add.12474 24400972

[29] Madden JT, Reyna NC, Pentkowski NS. Antagonizing serotonin 2A (5-HT_2A_) receptors attenuates methamphetamine-induced reward and blocks methamphetamine-induced anxiety-like behaviors in adult male rats. Drug Alcohol Depend. 2020;215:108178. doi:10.1016/j.drugalcdep.2020.108178 32739601

[30] Madden JT, Reyna NC, Goranson EV, Gonzalez TA, Zavala AR, Pentkowski NS. Blocking serotonin 2A (5-HT_2A_) receptors attenuates the acquisition of methamphetamine-induced conditioned place preference in adult female rats. Behav Brain Res. 2021;415:113521. doi:10.1016/j.bbr.2021.113521 34391796

[31] McKetin R, Lubman DI, Lee NM, Ross JE, Slade TN. Major depression among methamphetamine users entering drug treatment programs. Med J Aust. 2011;195(3):S51-S55. doi:10.5694/j.1326-5377.2011.tb03266.x 21806520

[32] Duncan Z, Kippen R, Sutton K, . Anxiety and depression among a community-recruited cohort of people who use methamphetamine: a longitudinal analysis. Addiction. 2025;120(4):697-710. doi:10.1111/add.16714 39545452

[33] Kiluk BD, Carroll KM, Duhig A, . Measures of outcome for stimulant trials: ACTTION recommendations and research agenda. Drug Alcohol Depend. 2016;158:1-7. doi:10.1016/j.drugalcdep.2015.11.004 26652899 PMC4698050

[34] Bharat C, Webb P, Wilkinson Z, . Agreement between self-reported illicit drug use and biological samples: a systematic review and meta-analysis. Addiction. 2023;118(9):1624-1648. doi:10.1111/add.16200 37005867

[35] Carter G, Spittal MJ, Glowacki L, . Diagnostic accuracy for self-reported methamphetamine use versus oral fluid test as the reference standard in a methamphetamine-dependent intervention trial population. Addiction. 2023;118(3):470-479. doi:10.1111/add.16085 36367075 PMC10952224

