# Supplementary material for: Mirtazapine for Methamphetamine Use Disorder: A Randomized Clinical Trial
Source: JAMA Psychiatry. 2026 Apr 1;83(6):581–9. doi: 10.1001/jamapsychiatry.2026.0159 (PMC13044789; doi:10.1001/jamapsychiatry.2026.0159)
Supplement: Supplement 1. — Trial Protocol [file jamapsychiatry-e260159-s001.pdf]

The TINA trial:
A Phase 3 randomised placebo-controlled trial of mirtazapine as a pharmacotherapy for methamphetamine (“Ice”) dependence

Short title: The Tina Trial

Protocol ID: Tina Trial

ANZCTR Number: ACTRN12622000235707

The Universal Trial Number (UTN): U1111-1271-8220

Coordinating Principal Investigator: Rebecca McKetin

Coordinating Sponsor (referred to as the Sponsor): University of New South Wales

This research is being conducted under a co-sponsor arrangement involving the University of New South Wales, Deakin University, the University of Wollongong, Monash University, and the University of Sydney

Funded by: Medical Research Future Fund (Grant No. 2007155)

Version Number: 13.0

13th August 2025

Contents

1 STATEMENT OF COMPLIANCE 5
2 General information 6
3 PROTOCOL SUMMARY 14
4 INTRODUCTION 16
4.1 Background and study rationale 16
4.2 Aims and hypotheses 17
5 STUDY POPULATION 18
5.1 Target sample 18
5.2 Inclusion criteria 18
5.3 Exclusion criteria 18
5.4 Lifestyle considerations 18
5.5 Screen failures 19
6 STUDY METHODS 20
6.1 Design 20
6.2 Schema 20
6.3 Schedule of activities 21
6.4 Measures 23
6.5 Randomisation and blinding 26
6.6 End of study definition 26
6.7 Treatment at the end of the study 27
6.8 Reporting study outcomes to participants 27
7 STUDY INTERVENTION 28
7.1 Description 28
7.2 Justification 28
7.3 Dosing and administration 28
7.4 Medication acquisition, storage and handling 29
7.5 Medication adherence 32
7.6 Rescue medicine 32
8 SAFETY 33
8.1 General requirements 33
8.2 Medical review 33
8.3 Definitions and coding of Adverse Events 33
8.4 Assessing, reporting and reviewing adverse events 36
8.5 Significant safety issues and urgent safety measures 38
8.6 Protocol deviations 39
8.7 Serious breaches of good clinical practice 39
8.8 Unanticipated Problems 41
8.9 Events of special interest 42
8.10 Monitoring concomitant medications 42

8.11 Medication discontinuation and participant withdrawal ... 42
8.12 Study discontinuation ... 44
9 RISK/BENEFIT ASSESSMENT ... 45
9.1 Known potential risks ... 45
9.2 Known potential benefits ... 52
10 STATISTICAL CONSIDERATIONS ... 53
10.1 Sample size determination ... 53
10.2 Populations for analyses ... 53
10.3 Statistical Analyses ... 53
11 GOVERNANCE ... 56
11.1 Administration ... 56
11.2 Monitoring ... 58
11.3 Data safety and monitoring board ... 59
11.4 Data management ... 59
11.5 Publication and data sharing policy ... 61
11.6 Conflict of interest policy ... 62
12 STUDY PROCEDURES ... 62
12.1 Informed Consent process ... 62
12.2 Recruitment ... 63
12.3 Retention ... 64
12.4 Reimbursement for participation ... 65
12.5 Managing workflow and assessment workload ... 65
12.6 Field interviewing procedures ... 65
12.7 Assignment of study identifiers ... 67
12.8 Activation and stopping of MEMS Smartcaps ... 70
12.9 Saliva collection and handling procedures ... 71
12.10 Electronic data management and REDCap ... 72
12.11 COVID related procedures ... 73
13 DETAILS OF STUDY VISITS AND OTHER ASSESSMENTS ... 74
13.1 Overview ... 74
13.2 Phone screening ... 74
13.3 Eligibility assessment (Visit 1) ... 74
13.4 Medical screening (Visit 2) ... 75
13.5 Run-in period (confirmation of eligibility and randomisation) ... 75
13.6 Baseline assessment (Visit 3) ... 76
13.7 Week 2 phone review (Visit 4) ... 77
13.8 Week 4, 8 and 12 assessments (Visits 5-7) ... 77
13.9 Week 16 phone assessment (Visit 8) ... 77
13.10 Final medical assessment (Visit 9) ... 77
13.11 Week 20 assessment (Visit 10) ... 78
13.12 Meetings between the Trial Researcher and Trial Physician ... 78

14 RELATED DOCUMENTS 79
15 ABBREVIATIONS 80
16 PROTOCOL AMENDMENT HISTORY 81
17 REFERENCES 87
18 APPENDICES 90
18.1 Appendix 1: Adaptation of the HIV Risk Behaviour Scale 90
18.2 Appendix 2: UNSW Clinical Trial Delegation Log 95

List of Tables

Table 1. Schedule of activities 22
Table 2. List of study endpoints and how they are measured 23
Table 3. Risks associated with mirtazapine and actions used in the trial to mitigate each of these risks 47
Table 4. Cautions with regard to specific conditions and populations, and actions taken in the trial to mitigate related risk 50

List of Figures

Figure 1. Trial flow diagram 21
Figure 2. Example of medication bottles with tear-off tab and space for recording participant information 31
Figure 3. Example of MEMS® calendar view 32
Figure 4. Reporting cycle used to monitor trial activity 58
Figure 5. System for allocating Screening ID 68
Figure 6. MEMS® SmartCap with LCD display and reader. 70
Figure 7. StatSure Saliva Sampler device 71
Figure 8. Instructions for the StatSure Saliva Sampler Device™ 72

1 STATEMENT OF COMPLIANCE

I agree to implement and conduct this study diligently and in strict compliance with the protocol. The clinical trial will be conducted in compliance with the following guidelines and documentation:

- ICH Guidelines for Good Clinical Practice (GCP).
- National Statement on Ethical Conduct in Human Research (National Statement).
- As approved by the Human Research Ethics Committee (HREC), the clinical trial protocol is responsible for monitoring the trial's conduct.
- The responsibilities set out by the UNSW Sponsors Delegate.
- The onsite or remote monitoring standard operating procedures as put in place by the clinical trial sponsor.

___________________________________	_______________________	___________
Site Lead Investigator<br>Printed Name	Signature	Date

___________________________________	_______________________	___________
Sponsor Representative<br>Printed Name and Title	Signature	Date

2 GENERAL INFORMATION

**Protocol Title**				
The Tina Trial: A phase 3 randomised placebo-controlled trial of mirtazapine as a pharmacotherapy for methamphetamine ("ice") dependence				
**Protocol identifying number**	The Universal Trial Number (UTN): U1111-1271-8220<br>HREC Approval #: 2021/ETH12037			
**Version Number**	13.0	**Version date**	13th August 2025	
**Amendment History**	See Section 16			
**Clinical Trial Sponsor**				
**Sponsor Name**	UNSW			
**Sponsor Contact**	Samela Husakovic, UNSW Sponsor's Delegate.			
**Email**	s.husakovic@unsw.edu.au			
**Address**	University of New South Wales Sydney NSW 2052			
**Coordinating Principal Investigator**				
**Name**	Professor Rebecca McKetin			
**Telephone**	02 9385 0294 Mobile: 0406538259			
**Email**	r.mcketin@unsw.edu.au			
**Type of Appointment with UNSW**	☒ UNSW Employee<br>☐ UNSW Conjoint<br>☐ Other (Please describe)			
**Trial personnel**				
**Chief Investigator A**				
**Name**	Prof Rebecca McKetin			
**Contact**	**Email**	r.mcketin@unsw.edu.au	**Telephone**	02 9385 0294
**Address**	National Drug and Alcohol Research Centre, University of New South Wales Sydney NSW 2052			
**Role**	Coordinating Principal Investigator			
**Chief Investigator B**				
**Name**	Prof Michael Farrell			
**Contact**	**Email**	Michael.farrell@unsw.edu.au	**Telephone**	02 9385 0333
**Address**	National Drug and Alcohol Research Centre, University of New South Wales Sydney NSW 2052			
**Chief Investigator C**				
**Name**	Prof Louisa Degenhardt			
**Contact**	**Email**	l.degenhardt@unsw.edu.au	**Telephone**	02 9385 0333
**Address**	National Drug and Alcohol Research Centre, University of New South Wales Sydney NSW 2052			
**Chief Investigator D**				
**Name**	Prof Gregory Dore			
**Contact**	**Email**	gdore@kirby.unsw.edu.au	**Telephone**	02 9385 0900
**Address**	Kirby Institute, Level 6, Wallace Wurth Building, High Street, UNSW, NSW 2052			

Chief Investigator E				
**Name**	Prof Steven Shoptaw			
**Contact**	**Email**	sshoptaw@mednet.ucla.edu	**Telephone**	+1 3107940619
**Address**	Department of Family Medicine, University of California, 10880 Wilshire Blvd, Suite 1800, Los Angeles, CA 90024			
**Chief Investigator F**				
**Name**	Prof Peter Kelly			
**Contact**	**Email**	pkelly@uow.edu.au	**Telephone**	02 4239 2382
**Site**	Wollongong			
**Role**	Site Lead			
**Address**	School of Psychology, Faculty of Social Sciences, Building 41, room 128. University of Wollongong NSW 2522			
**Chief Investigator G**				
**Name**	Dr Alyna Turner			
**Contact**	**Email**	a.turner@deakin.edu.au	**Telephone**	03 4215 3313
**Site**	Geelong			
**Role**	Site Lead			
**Address**	IMPACT Trials, School of Medicine, Deakin University. HERB Building Barwon Health, PO Box 281 Geelong 3220			
**Chief Investigator H**				
**Name**	Dr Philip J Clare			
**Role**	Statistician			
**Contact**	**Email**	Philip.clare@sydney.edu.au	**Telephone**	02 8627 8668
**Address**	Prevention Research Collaboration, The University of Sydney, Sydney, NSW, 2006			
**Chief Investigator I**				
**Name**	Dr Shalini Arunogiri			
**Contact**	**Email**	Shalini.arunogiri@monash.edu	**Telephone**	+61 390766552
**Address**	Alfred Psychiatry Research Centre (MAPrc), Central Clinical School, Monash University. C/-Medicine, Nursing and Health Sciences, Level 4, 607 St Kilda Rd, Melbourne VIC 3004			
**Chief Investigator J**				
**Name**	Ms Samantha Colledge			
**Contact**	**Email**	s.colledge@student.unsw.edu.au	**Telephone**	02 9385 0333
**Address**	National Drug and Alcohol Research Centre, University of New South Wales Sydney NSW 2052			
**Associate Investigator 1**				
**Name**	Prof Michael Berk			
**Contact**	**Email**	Michael.berk@deakin.edu.au	**Telephone**	03 4215 3320
**Site**	Geelong			
**Role**	Study medical expert			
**Address**	IMPACT Trials, School of Medicine, Deakin University. HERB Building Barwon Health, PO Box 281 Geelong 3220			

**Associate Investigator 2**				
**Name**	Prof Olivia Dean			
**Contact**	**Email**	o.dean@deakin.edu.au	**Telephone**	03 4215 3300
**Site**	Geelong			
**Address**	IMPACT Trials, School of Medicine, Deakin University. HERB Building Barwon Health, PO Box 281 Geelong 3220			
**Associate Investigator 3**				
**Name**	A/Prof David Goodman-Reza			
**Contact**	**Email**	dgoodman@kirby.unsw.edu.au	**Telephone**	N/A
**Address**	Kirby Institute, Level 6, Wallace Wurth Building, High Street,<br>UNSW Australia, Kensington NSW 2052			
**Associate Investigator 4**				
**Name**	Dr Barbara Sinclair			
**Contact**	**Email**	Barbara.Sinclair@health.nsw.gov.au	**Telephone**	02 4254 2700
**Site**	Wollongong			
**Role**	Trial Physician			
**Address**	Illawarra Drug and Alcohol Service, Illawarra Shoalhaven Local Health District, NSW Health. The Orana Centre, 2 Rawson St, Wollongong NSW 2500			
**Associate Investigator 5**				
**Name**	David Reid			
**Contact**	**Email**	david.reid1@health.nsw.gov.au	**Telephone**	02 4254 2700
**Site**	Wollongong			
**Address**	Illawarra Drug and Alcohol Service, Illawarra Shoalhaven Local Health District, NSW Health. The Orana Centre, 2 Rawson St, Wollongong NSW 2500			
**Associate Investigator 6**				
**Name**	Dr Harry Hill			
**Contact**	**Email**	h.hill@deakin.edu.au	**Telephone**	03 4215 8792
**Address**	IMPACT Trials, School of Medicine, Deakin University. HERB Building Barwon Health, PO Box 281 Geelong 3220			
**Associate Investigator 7**				
**Name**	Dr Jeremy Hayllar			
**Contact**	**Email**	Jeremy.Hayllar@health.qld.gov.au	**Telephone**	07 3138375764
**Site**	Brisbane			
**Role**	Site Lead and Trial Physician			
**Address**	Biala, Metro North Health; GPO Box 8161, Brisbane QLD 4001. Street address: 270 Roma St, Brisbane QLD 4000			
**Associate Investigator 8**				
**Name**	Dr Michael Christmass			
**Contact**	**Email**	Michael.Christmass@mhc.wa.gov.au	**Telephone**	08 9430 5966
**Site**	Perth			
**Role**	Site Lead and Trial Physician			
**Address**	Next Step Community Alcohol and Drug Service East Perth. 32 Moore Street, East Perth WA 6004			
**Associate Investigator 9**				
**Name**	Ms Juanita Koeijers (Community representative)			

**Contact**	**Email**	j.koeijers@unsw.edu.au	**Telephone**	0414 603 043
**Role**	Community Representative			
**Address**	C/- National Drug and Alcohol Research Centre, University of New South Wales Sydney NSW 2052			

**Personnel authorised to sign the protocol and the protocol amendment(s) for the Sponsor (ICH GCP 6.1.3)**	
**Name**	Professor Rebecca McKetin
**Telephone**	02 9585 0333
**Email**	r.mcketin@unsw.edu.au
**Address**	National Drug and Alcohol Research Centre, University of New South Wales Sydney NSW 2052

**Human Research Ethics Committees**		
**Human Research Ethics Committee #1**		
**Name**	St Vincents Hospital Sydney Human Research Ethics Committee	
**Status of ethical review**	☒ **Approved** ☐ **In progress** ☐ **To be submitted**	
**Trial Sites**	All sites (primary HREC), including<br>Illawarra Shoalhaven Local Health district Drug and Alcohol Services and the University of Wollongong<br>Barwon Health and Deakin University<br>Metro North Health, Brisbane<br>Next Step Alcohol and Drug Services, Perth<br>North Metropolitan Health Service Mental Health Research Ethics and Governance Office (NMHSMH REGO) additionally requested their own approval to cover Next Step Alcohol and Drug Services, Perth<br>Drug and Alcohol Services South Australia (DASSA), Adelaide<br>Townsville Hospital and Health Service, Townsville	
**Human Research Ethics Committee #2**		
**Name**	WA Health Central Human Research Ethics Committee	
**Status of ethical review**	☒ **Approved** ☐ **In progress** ☐ **To be submitted**	
**Trial Sites**	Perth	
**Human Research Ethics Committee #3**		
**Name**	UNSW Human Research Ethics Committee	
**Status of ethical review**	☒ **Approved** ☐ **In progress** ☐ **To be submitted**	
**Trial Sites**	Coordinating Sponsor (External approval of the Joint University of Wollongong and Illawarra Shoalhaven LHD Health and Medical HREC approval)	
**Funding for the Clinical Trial**		
**Funding Body Name**	Medical Research Future Fund	
**Amount of Funding**	$4,899,579.86	
**Regulatory Requirements**		
**Therapeutic Goods Administration Clinical Trial Notification**		☒ **Yes**<br>☐ **No**
**Insurance for Clinical Trial#**		
**UNSW**		
**Insurer**	AON Newline Australia Insurance Pty Ltd	

**Type of Insurance**	Clinical Trial Insurance

**Wollongong**	
**Insurer**	UniMutual
**Type of Insurance**	Clinical Trials Protection
**Geelong**	
**Insurer**	AON
**Type of Insurance**	Medical Malpractice Clinical Trials, Medical Malpractice, Professional Indemnity, Public and Products Liability
**Brisbane**	
**Insurer**	Queensland Government Insurance Fund
**Type of Insurance**	Property, General Liability (includes Public and Products Liability), Professional Indemnity, Medical Indemnity, Personal Accident & Illness (Volunteers, Board Members & Committee Members)
**Perth**	
**Insurer**	RiskCover
**Type of Insurance**	General Liability (including Products Liability), Medical Treatment Liability, Professional Liability
**Adelaide**	
Insurer	SA Health's Self-Insured Program
Type of Insurance	Professional Indemnity (including medical malpractice), Public and Products Liability, Directors and Officers Liability, Personal Accident/volunteers
**Townsville**	
**Insurer**	Queensland Government Insurance Fund
**Type of Insurance**	Property, General Liability (includes Public and Products Liability), Professional Indemnity, Medical Indemnity, Personal Accident & Illness (Volunteers, Board Members & Committee Members)
# Insurance policies can be obtained by the Sponsor or relevant clinical trial site. Insurance policies will be monitored for currency throughout the trial by the Sponsor.	

Safety and Monitoring Contacts	
**Sponsor's Medical Expert†**	
**Name**	Professor Michael Berk
**Telephone**	03 4215 3320
**Email**	Michael.berk@deakin.edu.au
**Address**	IMPACT Trials, School of Medicine, Deakin University, HERB Building Barwon Health, PO Box 281 Geelong 3220
**Site Qualified Physicians**	
**Wollongong:**	
**Name**	Dr Barbara Sinclair
**Telephone**	02 4254 2700
**Email**	Barbara.Sinclair@health.nsw.gov.au
**Address**	The Orana Centre, 2 Rawson St, Wollongong, NSW, 2500
**Name**	Dr Frank Cordaro
**Telephone**	02 42542700
**Email**	Frank.Cordaro@health.nsw.gov.au
**Address**	The Orana Centre, 2 Rawson St, Wollongong, NSW, 2500
**Geelong:**	
**Name**	Dr Robert Lundin
**Telephone**	robert.lundin@deakin.edu.au
**Email**	0478691460
**Address**	Drug and Alcohol Services, Barwon Health, Level 1, 126 Little Malop St Geelong, VIC, 3220
**Name**	Dr Sarangi Nanayakkara
**Telephone**	0483827954
**Email**	Sarangi.Nanayakkara@barwonhealth.org.au
**Address**	Barwon Health. Ryrie Street, Geelong 3220 , VIC
**Name**	Dr Antigone Branchflower
**Telephone**	0431170952
**Email**	Antigone.Branchflower@barwonhealth.org.au
**Address**	Drug and Alcohol Services, Barwon Health, Level 1, 126 Little Malop St Geelong, VIC, 3220
**Brisbane:**	
**Name**	Dr Jeremy Hayllar
**Telephone**	(07) 31 3837 5764
**Email**	Jeremy.Hayllar@health.qld.gov.au
**Address**	Biala, Metro North Health. GPO Box 8161, Brisbane QLD 4001 (Street address: 270 Roma St, Brisbane QLD 4000)
**Name**	Dr Catherine Llewellyn
**Telephone**	(07) 31 3837 5666
**Email**	Catherine.Llewellyn@health.qld.gov.au
**Address**	Biala, Metro North Health. GPO Box 8161, Brisbane QLD 4001 (Street address: 270 Roma St, Brisbane QLD 4000)
**Name**	Dr Nikola Ognyenovits
**Telephone**	0435 082 626
**Email**	nikola.ognyenovits@health.qld.gov.au

**Address**	Biala, Metro North Health. GPO Box 8161, Brisbane QLD 4001 (Street address: 270 Roma St, Brisbane QLD 4000)
**Perth:**	
**Name**	Dr Michael Christmass
**Telephone**	(08) 9430 5966
**Email**	Michael.Christmass@mhc.wa.gov.au
**Address**	Next Step Community Alcohol and Drug Service East Perth, 32 Moore St, East Perth WA, 6004
**Adelaide:**	
**Name**	Dr Will Liaw
**Telephone**	08 7485 5168
**Email**	Willy.Liaw@sa.gov.au
**Address**	Drug and Alcohol Services SA, 91 Magill Road, Stepney, SA, 5069
**Name**	Dr Amelia Woods
**Telephone**	0434841910
**Email**	Amelia.Woods@sa.gov.au
**Address**	Drug and Alcohol Services SA, 91 Magill Road, Stepney, SA, 5069
**Name**	Dr Blaire Brewerton
**Telephone**	08 7485 4600
**Email**	blaire.brewerton@sa.gov.au
**Address**	Northern DASSA, 22 Langford Dr, Elizabeth SA, 5112
**Townsville:**	
**Name**	Dr Ellie Holyoak
**Telephone**	07 4433 3000
**Email**	Ellie.Holyoak@health.qld.gov.au
**Address**	ATODS, 190 Palmerston Street, Vincent, QLD, 4814
**Name**	Dr Brian Wu
**Telephone**	0426832311
**Email**	Brian.Wu@health.qld.gov.au
**Address**	ATODS, 190 Palmerston Street, Vincent, QLD, 4814
**Name**	Dr Hayley Maher
**Telephone**	0493 152 254
**Email**	Hayley.Maher@health.qld.gov.au
**Address**	ATODS, 35-43 Gregory St, North Ward QLD 4810.

**Pharmacy contacts**	
**Wollongong:**	
**Name**	Melissa Inskip
**Telephone**	02 4222 5342
**Email**	Melissa.Inskip@health.nsw.gov.au, islhd-twh-pharmacy-trials@health.nsw.gov.au
**Address**	Pharmacy Department Level 4, Wollongong Hospital, Loftus Street, Wollongong, NSW, 2500

Geelong	
**Name**	Tracey Shields & Alicia Neels
**Telephone**	03 4215 1587
**Email**	trialsuhg@barwonhealth.org.au
**Address**	Barwon Health Pharmacy Department, University Hospital Geelong, PO Box 281, Geelong Victoria 3220
**Brisbane**	
**Name**	Michael Rayner
**Telephone**	(07) 364 70468
**Email**	Michael.rayner3@health.qld.gov.au
**Address**	Clinical Trials Pharmacy Department; Level 1, Ned Hanlon Building; Royal Brisbane and Women's Hospital, Butterfield St, Herston Qld 4029
**Perth**	
**Name**	Amelia Arandiga
**Telephone**	(08) 6159 6400
**Email**	amelia.arandiga@health.wa.gov.au
**Address**	Graylands Hospital Pharmacy Department 1 Brockway Road Mount Claremont WA 6010
**Adelaide**	
**Name**	Mariana Nasr
**Telephone**	(08) 7425 5070
**Email**	Mariana.Nasr@sa.gov.au
**Address**	DASSA Pharmacies Drug and Alcohol Services SA/ SA Pharmacy, 91 Magill Road, Stepney, SA, 5069
**Townsville**	
**Name**	Jenny Walsh and Steven Capell
**Telephone**	(07) 4433 4117
**Email**	jenny.walsh2@health.qld.gov.au
**Address**	Townsville Hospital and Health Service, 100 Angus Smith Drive, Douglas, QLD 4814

Data Safety and Monitoring Board members	
Prof Jason White (Chair)	Jason.White@unisa.edu.au
Prof Madhukar Trivedi	madhukar.trivedi@utsouthwestern.edu
A/Prof Timothy Schlub (Statistician)	tim.schlub@sydney.edu.au
Mr Matthew Ryan	mattyr469@gmail.com

Trial Management Group
The Trial Management Group is comprised of the Investigator Team or delegates thereof (see section 11.1.3 of protocol).

†Additional independent medical expertise and oversight will be provided through the Data and Safety Monitoring Board.

3 PROTOCOL SUMMARY

Title: A phase 3 randomised placebo-controlled trial of mirtazapine as a pharmacotherapy for methamphetamine (“ice”) dependence

Short title: The Tina Trial

Study Description: This is a multi-site Phase 3 randomised (1:1), double-blind, placebo-controlled parallel trial (N = 340) designed to confirm whether take-home mirtazapine (30 mg/day) is a safe and effective pharmacotherapy for methamphetamine dependence when delivered in routine clinical practice in Australia.

Objectives:

Primary Objective: To test whether daily oral mirtazapine (30 mg/day) delivered as a take home medication will reduce self-reported days of methamphetamine use relative to placebo.

Secondary Objectives: To test whether daily oral mirtazapine (30 mg/day) increases methamphetamine negative oral fluid samples; reduces depressive symptoms; improves sleep; reduces HIV risk behaviour; improves quality of life, has an acceptable adverse event profile; and, does not significantly increase the use of other substances.

Endpoints:

Primary Endpoint:

Self-reported days of methamphetamine use in the 28 days prior to the week 12 assessment, assessed using the Timeline Followback (TLFB).

Secondary Endpoints:

Methamphetamine negative oral fluid tests (< 25 ng/mL methamphetamine in oral fluid sample) taken at weeks 4, 8 and 12.

Depressive symptoms: Total score on the Patient Health Questionnaire -9 (PHQ-9).[2]

Sleep Quality: Total score on the Athens Insomnia Scale.[3]

HIV risk behaviour: Total score on a modified version of the HIV Risk Behaviour Scale from the Opiate Treatment Index.[4]

Quality of life: Utility score on the EuroQol-5D-5L (EQ-5D)[5]

Safety Endpoints:

Adverse events: Percentage of participants who report Adverse Events (AEs) and Serious Adverse Events (SAEs) during the 12-week active medication phase.

Tertiary/Exploratory endpoints:

Suicidality: Score of 3+ on the Columbia Suicide Severity Risk Scale – Screener (CSSRS-S) [6,7] at any time in during the 12-week active medication phase.

Other substance use: Total number of reported days use for all other major drug classes (tobacco, alcohol, cannabis, cocaine, ecstasy, hallucinogens, inhalants, heroin) in the past 4 weeks at week 12 of the active medication phase.

Concomitant medications: All medication received by participants will be recorded on a template adapted from the National Institute of Health Concomitant Medications Form.[8]

Health Economics data will be collected to facilitate any subsequent economic evaluation, including the EuroQol V2.1,[5] the Work Productivity and Activity Impairment Questionnaire – General Health V2,[9] and contact with health services and the criminal justice system.

The *participant's impression* will be assessed using the Patient Global Impression (PGI) scale.[10]

Treatment satisfaction will be assessed with both the Treatment Satisfaction Questionnaire for Medication II (TSQM-II)[11] * and the unpublished Medication Tolerability Scale.

Anxiety will be assessed using the Generalised Anxiety Disorder 7 Item (GAD – 7) Scale.

Study Population: 340 people who are dependent on methamphetamine in the past year and currently using methamphetamine (at least twice weekly use in the past 4 weeks) and aged 18-65 years.

Phase: Phase 3

Description of Sites/Facilities Enrolling Participants:

The trial will recruit 240 of 340 participants through four main trial sites (aiming for 60 participants recruited through each site):
(1) Deakin University via Barwon Health in Geelong (CIs Turner, AIs Berk, Dean and Hill),
(2) Wollongong University via the Illawarra Drug and Alcohol Services at the Illawarra and Shoalhaven Local Health District (CI Kelly, AIs Sinclair and Reid),
(3) Metro North Health, Brisbane, QLD (AI Hayllar),
(4) Next Step Community Alcohol and Drug Service; Perth WA (AI Christmass).

The balance of participants will be recruited through two additional trial sites (below) and an extension of recruitment at Metro North Health, Brisbane, QLD (AI Hayllar) and other primary sites where feasible.
- Drug and Alcohol Services South Australia (DASSA) in Adelaide (PI Will Liaw),
- Townsville Hospital and Health Service, QLD (PI Ellie Holyoak)

Description of Study Intervention: 30mg oral of Mirtazapine (or matched placebo) daily for 12 weeks. All participants will receive a minimal intervention (the self-help booklet, *On Ice*).

Study Duration: 4 years

Participant Duration: 24 weeks

4 INTRODUCTION

4.1 BACKGROUND AND STUDY RATIONALE

Crystalline methamphetamine (aka "ice") is a significant and growing public health concern in Australia, with ~300,000 Australians now dependent on the drug[12] costing the Australian government $5 billion per annum[13] and having widespread personal and social impacts.[14]

Dependence on methamphetamine is a chronic relapsing condition[15] associated with significantly elevated mortality, increased incidence of HIV and hepatitis C infection, poor mental health (suicidality, psychosis, depression, and violence), and increased risk of cardiovascular events.[14] This has a substantial impact on clinical health care services. In Australia: methamphetamine use accounts for between 28,400 and 80,900 additional psychiatric hospital admissions per year, and between 29,700 and 151,800 additional emergency department presentations per year.[16]

The Australian alcohol and drug treatment sector is unable to cope with growing demand for methamphetamine treatment (with episodes of care increasing from 10,027 in 2009/10 to 58,176 in 2018/19*)*.[17] Fewer than one in ten people receive minimally adequate treatment.[18,19] Available treatment options (e.g., long-stay residential rehabilitation) carry substantial access, cost and scale capacity constraints,[14] yet do not convey lasting benefit.[15] Pharmacotherapy provides a potentially scalable cost-effective treatment option that could dramatically increase treatment coverage and respond to the fundamental nature of methamphetamine use as a chronic relapsing condition.[20] Currently there are no TGA approved pharmacotherapies[20,21] to treat methamphetamine use. Recent efforts favour a combination of injectable naloxone and bupropion (single trial, N = 403[22]) and there are ongoing efforts looking at various approaches (e.g., long-acting slow-release amphetamine preparations[23]). However, currently there is insufficient evidence to support any pharmacotherapy option for methamphetamine use.

Box 1. Mirtazapine's mechanism of action on methamphetamine use

Preclinical research has found that mirtazapine reduces drug-seeking behaviour in rodent addiction models, while chronic exposure reduces the neuroplastic changes that develop with methamphetamine addiction.[1] This research points toward a specific role of mirtazapine on serotonin (5-HT) receptors which are upstream of dopamine reward pathways in the brain and which modulate the signalling in these pathways.[1] Specifically, mirtazapine has high affinity for 5-H_{2A}, 5-HT_{2C} and 5-HT_3 receptor subtypes. 5-HT_{2A} receptors are involved in the development of sensitisation to methamphetamine, which underpins addiction, via the upregulation of dopamine function. 5-HT_{2C} receptors indirectly inhibit dopamine function (via their localisation in GABAergic neurons throughout the dopamine mesolimbic and mesocortical pathways) and 5-HT_{2C} agonists reduce drug-seeking in rodents. Mirtazapine antagonises 5-HT_3 receptors, which reduces methamphetamine's effects and reduces relapse to drug taking in rodents. Chronic antidepressant administration also modulates the mRNA editing of the metabotropic 5-HT_{2A} receptors and contributes to reduced drug seeking.

Our systematic review identified mirtazapine, an approved generic antidepressant medication, as a promising candidate,[14] with positive results from two Phase 2 trials.[24,25] The first trial (N = 60) found 12 weeks of mirtazapine (30 mg/day) significantly reduced methamphetamine use relative to placebo.[25] These positive results were recently replicated on a larger sample (N = 120) with 24 weeks of mirtazapine treatment.[24] Mirtazapine (30 mg/day) reduced methamphetamine positive urine test results at 24 weeks relative to placebo (63% vs. 74%; RR 0.75, 95% CI 0.56 – 1.00, p = 0.04), with additional improvements seen in depressive symptoms (Centre for Epidemiologic Studies Depression Scale score; b = -6.2, p = 0.01) and insomnia (Athens Insomnia Scale; b = -1.4, p = 0.04). Mirtazapine was well-tolerated and there were no serious adverse reactions. The possible mechanisms underpinning these benefits of mirtazapine in methamphetamine dependence are discussed in Box 1.

Mirtazapine reduced methamphetamine use by 15-18%,[24,25] which should produce clinically meaningful health benefits. The number of people who need to be treated with mirtazapine to get 1 person to respond to the treatment is 8, which is similar to medications for other addictive substances.[26] Being a generic and available medication, mirtazapine could be readily integrated into general practice as a pharmacotherapy for methamphetamine dependence. Mirtazapine's antidepressant properties present an additional benefit (~ 84% of methamphetamine treatment entrants have clinically significant symptoms of depression[27]). Treatment with popular selective serotonin re-uptake inhibitors is contraindicated for people who use methamphetamine.[28] In contrast, mirtazapine has been found to reduce depressive symptoms and is well-tolerated.[24]

No Phase 3 trials of mirtazapine for methamphetamine dependence have been registered in Australia or elsewhere. Demonstration of efficacy in previous Phase 2 trials indicates a need for a large-scale definitive Phase 3 trial to demonstrate effectiveness and safety in routine clinical practice. A larger Phase 3 trial is also needed to show benefits generalise to the broader population of people dependent on methamphetamine: previous trials were limited to small samples of men and excluded comorbidities (including major depression). Replication of benefits in a broad sample will allow real-world generalisability and uptake.

The Tina Trial is a multisite Phase 3 trial that will determine whether mirtazapine has a clinically relevant benefit on methamphetamine use when delivered in routine clinical practice. It is a double-blind randomised placebo-controlled study. Participants will be dependent on methamphetamine (N = 340) and will be randomised (1:1) to receive either 12 weeks of mirtazapine (30 mg/day) or matched placebo. The primary endpoint is days of methamphetamine use; secondary endpoints are abstinence from methamphetamine use, depression, sleep, HIV risk behaviour and quality of life. Safety and tolerability will also be assessed. A pragmatic and inclusive trial design will be used to establish realistic treatment effects and facilitate translation into practice.

4.2 AIMS AND HYPOTHESES

Aim: To establish the effectiveness and safety of mirtazapine as an outpatient pharmacotherapy for methamphetamine dependence in routine clinical practice using a double-blind randomised placebo-controlled controlled trial design.

Primary hypothesis: Oral mirtazapine (30 mg/day) for 12 weeks will, compared to placebo, reduce self-reported days of methamphetamine use.

Secondary hypotheses: Oral mirtazapine (30 mg/day) for 12 weeks will, compared to placebo: increase periods of abstinence from methamphetamine use (i.e., methamphetamine negative oral fluid samples); reduce depressive symptoms; improve sleep; reduce HIV risk behaviour, improve quality of life; have an acceptable adverse event profile; and, not significantly increase the use of other substances.

5 STUDY POPULATION

5.1 TARGET SAMPLE

The target study sample is 340 people who are dependent on methamphetamine.

5.2 INCLUSION CRITERIA

- Aged between 18 and 65 years
- Moderate to severe methamphetamine use disorder in the past year (DSM-5 past year diagnosis confirmed at Eligibility Assessment using modified version of the Composite International Diagnostic Interview)
- Current methamphetamine use (defined as using at least twice weekly in past 4 weeks based on the participant's self-reported use, and a positive drug screening test for methamphetamine)
- Willing to use effective contraception (for women only)
- Willing to provide contact details for their treating physician
- Willing to provide contact details for follow-up
- Able to provide informed consent and able to comply with both the requirements of the informed consent and the treatment protocol

5.3 EXCLUSION CRITERIA

- In need of acute care (e.g., suicidality or acute psychosis, unstable psychiatric condition; medical detoxification)
- Pregnant or lactating
- Incarceration or current inpatient treatment (including residential rehabilitation, inpatient detoxification); this applies to the status of the participant at trial enrolment and does not preclude the participant from entering treatment or receiving usual care during the trial
- Currently taking prescribed antidepressant medication
- Any use of monoamine oxidase inhibitors in the 14 days prior to starting the trial medication
- Contraindications for mirtazapine, including:
    - Known hypersensitivity to mirtazapine
    - Use of antidepressant medication (including monoamine oxidase inhibitors, St. John's Wort, or SSRIs) other serotonergic drugs.
    - Galactose intolerance, Lapp lactase deficiency or glucose-galactose malabsorption (lactose is an excipient in the trial medication)
- High risk of adverse reactions to mirtazapine including suicide, overdose, sudden cardiac death, risk of agranulocytosis, or accidents and injuries from motor impairment
- Past year suicide attempt
- Unable or unwilling to avoid pregnancy during the trial (for both men and women)
- Participation in another clinical trial

5.4 LIFESTYLE CONSIDERATIONS

During this study, participants are asked to:

- Refrain from becoming pregnant (i.e., use effective contraceptive)
- Refrain from consuming other products containing mirtazapine and other antidepressant medication (including MAO inhibitors, SSRIs, St. John's Wort)
- Avoid driving or operating machinery if they are affected by drowsiness caused by the trial medication
- Avoid consuming depressant drugs that may increase the risk of drug overdose (e.g., opioids, sedatives, alcohol, antipsychotics)
- Inform the trial researchers if they become aware that they have a systemic medical disorder or other medical condition

5.5 SCREEN FAILURES

Screen failures are defined as participants who consent to participate in the clinical trial but are not subsequently randomly assigned to the study intervention or entered in the study because they were ineligible.

Individuals who do not meet the criteria for participation in this trial (screen failure) may be rescreened later (e.g., if the reason for ineligibility is temporary/reversible). If rescreening takes place within 28 days, data from the original eligibility assessment data can be retained, else if more than 28 days have elapsed this form needs to be redone. In all cases the medical screening and pregnancy test need to be redone.

6 STUDY METHODS

6.1 DESIGN

A multi-site Phase 3 randomised, double-blind, placebo-controlled parallel trial. 340 participants will be recruited across the sites. Participants will be randomly allocated (1:1) to receive either oral Mirtazapine 30mg daily, or matched placebo, for 12 weeks.

6.2 SCHEMA

The trial flow diagram is shown in Figure 1.

After phone screening, participants will be consented before undergoing an eligibility assessment and medical screening. Eligible participants will be randomly assigned to receive either mirtazapine or placebo (1:1 ratio) for 12 weeks. If participants are not randomised within 28 days of completing the eligibility assessment, the eligibility assessment needs to be redone.

The intervention phase of the study runs from the baseline assessment to the week 12 assessment. The first bottle of trial medication is provided at the baseline assessment. A phone review of AEs is conducted at around 2 weeks later to check for adverse reactions to the trial medication that require follow-up with the Trial Physician. Assessments at weeks 4, 8 and 12 are used to collect outcomes data. Medication bottles are replaced at weeks 4 and 8. The intervention medication bottles will be fitted with MEMS® Smartcaps to monitor medication adherence.

A post-intervention tapering dose of the trial medication will be provided at the week 12 assessment (15 mg/day for 28 days). At the end of this tapering dose, a phone assessment will be conducted to review AEs (week 16) followed by a final medical assessment (week 18). At the final medical assessment, the trial physician will discuss with the participant options for ongoing care, including other substance use treatment options and ongoing mirtazapine prescription, and provide referral as needed.

All medication bottles are to be retrieved from the patient and returned to pharmacy. A reply-paid envelope will be provided to participants to return any unused medication after the study period.

A final phone assessment at 20 weeks will assess the durability of treatment effects and treatment satisfaction.

Further details on each assessment can be found in Section 13.

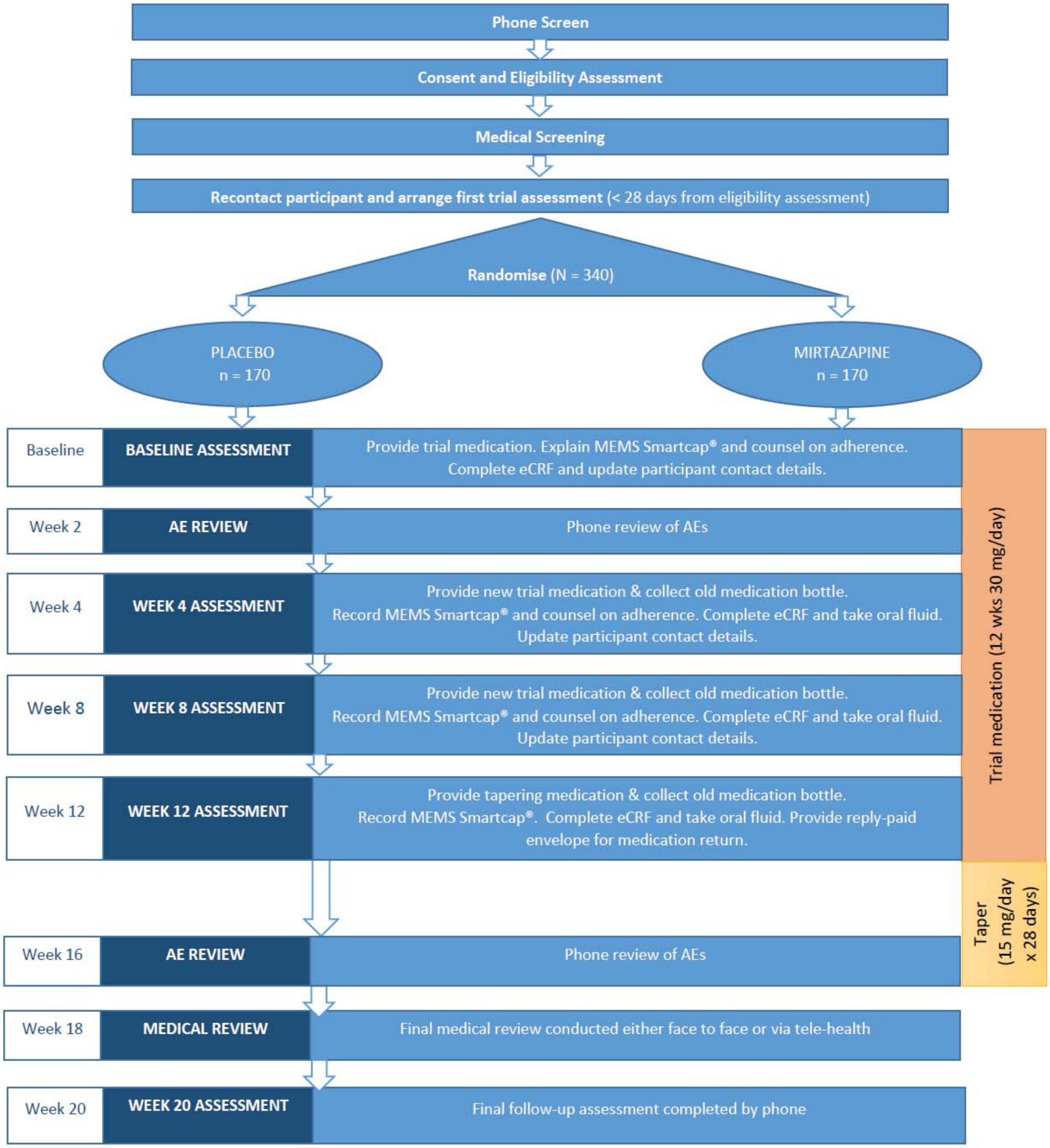

Figure 1. Trial flow diagram

6.3 SCHEDULE OF ACTIVITIES

The schedule of activities is shown in Table 1.

Table 1. Schedule of activities

	Screening & eligibility†				Intervention					Tapering	Post-medication f/up	
**Visit Number:**		**1**	**2**		**3**	**4**	**5**	**6**	**7**	**8**	**9**	**10**
**Name of assessment**	**Phone screen**	**Eligibility**	**Medical screen**	**Run-in**	**Baseline**	**AE review**	**Week 4**	**Week 8**	**Week 12**	**AE review§**	**Final medical#**	**Week 20**
**Week:**	**-3**	**-2**	**-2**	**-1**	**0**	**2**	**4**	**8**	**12**	**16**	**18**	**20**
**Day:**					**Day 0**	**Day 14 (-7 to +7)**	**Day 28 (-7 to +21)**	**Day 56 (-7 to +21)**	**Day 84 (-7 to +21)**	**Day 112 (-21 to +21)#**	**Day 126 (-7 to +35)**	**Day 140 (-7 to +21)**
**Phone Screen**	x											
**Participant Consent Form**		x										
**Participant Contact Form**		x			x		x	x	x			
**Drug Swipe Test**		x										
**Pregnancy Test**		x										
**Medical Review**			x								x	
**Eligibility Form**				x								
**Randomization**				x								
**Adverse Events Form**						x	x	x	x	x		x
**Assessment forms**												
Demographics and drug use history		x										
Methamphetamine use disorder (CIDI)		x										
Days of methamphetamine use (TLFB)		x			x		x	x	x			x
Days of other substance use		x			x		x	x	x			
Depression (PHQ-9)		x			x		x	x	x			x
Anxiety (GAD – 7)					x		x	x	x			
Sleep (AIS-5)					x		x	x	x			
HIV Risk (HRBS)					x		x	x	x			
Quality of life (EQ-5D)					x		x	x	x			
Concomitant medications		x			x		x	x	x			x
Suicidality screener (CSSRS-S)		x			x		x	x	x			
Patient centred assessment (PGI)							x	x	x			
Tolerability/satisfaction (TSQM II)*							x	x	x			x
Work productivity (WPA-GH)					x				x			
Health service use					x				x			x
**Oral fluid sample**							x	x	x			
**MEMS® reading**					x∞		x	x	x			

Notes. Timeline Followback (TLFB) , Patient Health Questionnaire–9 (PHQ-9), Athens Insomnia Scale (AIS), HIV Risk Behaviour Scale (HRBS), Euroqol 5D (EQ-5D), Columbia Suicide Severity Risk Scale Screener (CSSRSS), Patient Global Impression (PGI), Composite International Diagnostic Interview (CIDI) modified to capture a DSM 5 methamphetamine use disorder, the Generalised Anxiety Disorder 7 item scale (GAD-7), Work Productivity and Activity Impairment Questionnaire – General Health V2 (WPAI-GH) Treatment Satisfaction Questionnaire for Medication Version II (TSQM II). * The TSQM will be substituted with the unpublished Medication Tolerability Scale at weeks 4 and 8. †28 days are provided for screening and eligibility assessments prior to randomisation. Timing suggested is indicative only. §Should be conducted prior to final medical assessment so Trial Physician can review AEs #Needs to be at least 4 days after medication discontinuation. ∞Initiation of MEMS® SmartCap only. # Should be scheduled at least 7 days after the week 12 assessment. All assessments should be scheduled no sooner than 7 days after the previous assessment.

6.4 MEASURES

6.4.1 LIST OF STUDY ENDPOINTS

Table 2 contains a list of study endpoints, along with the measures used to assess each endpoint and the definition of the endpoint used in the statistical analysis.

Table 2. List of study endpoints and how they are measured

ENDPOINT	MEASURE	DEFINITION FOR ANALYSIS
**Primary**		
Days of methamphetamine use	Timeline Followback (TLFB)[29]	Self-reported days of methamphetamine use in the past 28 days at 12 weeks.<br><br>We have chosen 12 weeks as the primary endpoint because mirtazapine takes several weeks to take effect. We expect any benefits should be apparent within 12 weeks.<br><br>Self-reported methamphetamine use will be validated against methamphetamine positive oral fluid tests, which will be taken as a secondary outcome measure.
**Secondary**		
Abstinence from methamphetamine use	Methamphetamine negative oral fluid samples (< 25 mg / L)	Repeated methamphetamine negative oral fluid samples (< 25 mg / L) taken at weeks 4, 8 and 12
Depressive symptoms	Patient Health Questionnaire -9 (PHQ-9)[2]	Total score on the PHQ-9 at week 12
Sleep quality	Athens Insomnia Scale (AIS)[52]	Total score on the Athens Insomnia Scale at week 12
HIV risk behaviour	Modified version of the HIV Risk-taking Behaviour Scale from the Opiate Treatment Index[4] (see Appendix for details)	Total score on the modified HIV Risk-taking Behaviour Scale at week 12
Quality of Life	Utility score on the EuroQol-5D-5L[5]	Utility score on the EQ-5D-5L at week 12

**Tertiary/Exploratory**		
Other substance use	Self-reported days of other substance use in the past 4 weeks. (The TLFB calendar can be used to assist with recall as needed.)	Total days use for other drug classes (tobacco, alcohol, cannabis, cocaine, ecstasy, hallucinogens, inhalants, and heroin) in 28 days prior to the baseline and week 12 assessments.
Suicidality	Columbia Suicide Severity Rating Scale Screener (CSSRS-S)[6,7]	
Patient Impression	Patient Global Impression (PGI) scale.[10]	Score based on this single item scale at week 12
Treatment satisfaction	Treatment Satisfaction Questionnaire for Medication Version (TQSM II)[11] will be used to assess tolerability at weeks 12 and 20. The unpublished Medication Tolerability Scale will be used instead at weeks 4 and 8.	Scores on the TSQM II at week 12.[11]
Medication adherence	MEMS adherence data from MEMS® SmartCap readings.	% of non-missed doses, where a non-missed dose is taking at least one dose within a 24-hour period (from 3 am) during the 12-week intervention phase.
Anxiety	Generalised anxiety disorder 7 Item (GAD-7) Scale score	Total score on the GAD-7 at week 12
**Costing data**		
Quality of life	EQ-5D-5L[5] version 2.1	Utility score on the EQ-5D at week 12
Work productivity	Work Productivity and Activity Impairment Questionnaire – General Health V2 (WPAI-GH)[9]	Score on the WPAI-GH at week 12
Health service use	Self-reported health service use assessed retrospectively using a structured questionnaire at the week 12 assessment.	Self-reported number of attendances to the following health services since starting the trial medication:<br>- Drug treatment, including specialist alcohol and other drug treatment services, other support (mutual aid/narcotics anonymous, SMART recovery) and opioid agonist therapy<br>- General or psychiatric hospital (incl. length of stay)<br>- Emergency department visit (incl. if admitted to hospital)<br>- Ambulance (incl. whether taken to hospital)<br>- General practitioner<br>- Counsellors/psychologist<br>- Dentists<br>- Psychiatrists<br>- Other health services
Criminal justice contact	Self-reported contact with the criminal justice system assessed retrospectively using a structured questionnaire at the week 12 assessment.	Number of arrests, court appearances and days detained since starting the trial medication.
**Safety analysis**		
Adverse event profile	Self-report solicited using semi-structured form	The percentage of participants reporting adverse events (AEs), and serious adverse events (SAEs), by System Organ Classification, coded according to the Medical Dictionary for Regulatory Activities (MedDRA). AEs will be counted once only for a given participant.

6.4.2 DETAILS OF SCALES USED

Following are details of the published scales used in the study.

Timeline Followback (TLFB)[29] The TLFB is a validated self-report measure of psychoactive substance use and the recommended outcome measure for stimulant trials.[30] The TLFB shows 88% sensitivity, 96% specificity, and a 95% hit-rate and 0.77 test-retest agreement, for the use of amphetamines in the past 30 days.[31]

The Patient Health Questionnaire -9 (PHQ-9)[2] is a brief 9-item questionnaire that has excellent internal consistency (Cronbach's alpha of 0.86 & 0.89) and test-retest reliability, and which has good construct validity against other measures, with a score of >=10 having 88% sensitivity and 88% specificity against a diagnosis of major depression.

The Athens Insomnia Scale (AIS)[52] is an 8-item self-report measure which is reliable (0.9 test-retest reliability) and has been validated against other measures of sleep quality.[52] The AIS is sensitive to sleep improvements induced by mirtazapine.[21]

The HIV Risk-taking Behaviour Scale (HRBS)[4] is a validated and reliable scale which provides a composite risk index for injecting and sexual behaviour. To ensure the relevance and brevity of the scale, we have removed out-dated items on cleaning needles with bleach, and retained only the most informative items on HIV risk taking. Details of this modification can be found in the appendix.

Columbia Suicide Severity Rating Scale Screener (CSSRS-S)[6,7] The CSSRS-S is a brief validated scale that is used to assess suicide risk. [5] A score of 3 or more on the CSSRS-S predicts a significant increase in the risk of subsequent suicide attempts.[6]

Treatment Satisfaction Questionnaire for Medication Version II (TSQM II)[11] assesses the participants perspective on whether they find the medication convenient, tolerable (in terms of side effects), effective, and it includes a global satisfaction summary score.

Medication Tolerability Scale (MTS). This unpublished scale has been designed as an alternative to the TSQM II. Data from the trial will be used to validate the scale.

Work Productivity and Activity Impairment Questionnaire – General Health V2 (WPAI-GH) is a quantitative measure of health-related work productivity loss designed for use in clinical trials.[9] The generic questionnaire can be adapted for specific health problems and has been used widely for other mental health conditions, but has not been validated for methamphetamine use specifically.

The Generalised Anxiety Disorder 7 Item (GAD-7) Scale is a valid and efficient tool for screening for GAD and assessing its severity in clinical practice and research.[32]

The Patient Global Impression (PGI) scale[10] is a single item scale that asks the patient whether their health status has improved since the start of the trial.

6.5 RANDOMISATION AND BLINDING

6.5.1 RANDOMISATION

Allocation to group: Participants will be randomly assigned to receive either the placebo or mirtazapine.

Randomisation sequence: The randomisation sequence is based on a 1:1 (treatment : placebo) permutated block randomisation, with variable block sizes, stratified by site (Geelong, Wollongong, Perth, Brisbane, Other site), sex (male vs. female) and PHQ-9 depression score (≥10 based on the eligibility assessment) – factors known to affect methamphetamine treatment trial outcomes. The PHQ-9 cut-point reflects a DSM-IV diagnosis of major depression.[2] There is no target sample size for the stratification groups of sex and depression. It is expected that around 60% of participants will be male and the majority will exceed the PHQ-9 cut-off for depression.

Preparation of the randomisation schedule: The randomisation schedule will be generated by the independent DSMB statistician using a randomisation program provided by the Sponsor. The DSMB statistician will insert a random seed number to the program code, and forward the randomisation schedule direct to each of the trial site pharmacies, to ensure that the Sponsor and other investigators and trial staff are blind to group allocation.

Replacement: Participants who are randomised, but who do not receive the study intervention, may not be replaced. Participants who are randomised, and receive the study intervention, and subsequently withdraw, or are withdrawn from the study, will not be replaced.

6.5.2 BLINDING

Packaging and the appearance of the medication in each group will be identical to conceal treatment allocation. All trial staff, the trial statistician and investigators will be blind to the group allocation. Participants will be asked what group they think that they are in (mirtazapine or placebo). A statistical analysis plan will be finalized prior to unblinding. The analysis will be blind to condition.

6.5.3 UNBLINDING

Unblinding can be done via an online portal by entering the participant's unique Study ID. This process will automatically notify the Sponsor of the unblinding via email. Unblinding can also be done via the randomisation schedule held by pharmacy.

Unblinding will be done at the discretion of either the DSMB, the Site Lead Investigator, or the Trial Physician. It can also be done by a treating medical physician in a medical emergency where the treatment of a patient requires knowledge of the medication being taken (e.g., because the event is thought to be related to the trial medication, or because it may affect treatment responses).

Group allocation can be shared with the DSMB for the purposes of monitoring adverse events if the DSMB deem this to be necessary. This will be done by the DSMB statistician.

Further details on unblinding procedures are provided in the Pharmacy Manual.

6.6 END OF STUDY DEFINITION

A participant is considered to have completed the study if he or she has completed the final assessment or the last possible scheduled date for the final assessment has lapsed (e.g., in the event that the participant cannot be contacted for follow-up visits) as shown in the Schedule of Activities in Section 6.3.

6.7 TREATMENT AT THE END OF THE STUDY

All participants will be provided with a Referral Sheet, which will include details of relevant local agencies through which they can receive further support for their substance use or related issues (see Section 13.8).

The trial physician will discuss options for ongoing treatment for substance use and provide appropriate referrals. If the participant wishes to continue taking the study drug, the trial physician will discuss this possibility with them, and provide a referral to their treating physician to facilitate this ongoing off-label prescribing of mirtazapine (see Section 13.10).

6.8 REPORTING STUDY OUTCOMES TO PARTICIPANTS

Participants will be informed about the study results via two mechanisms.

(i) Aggregate results will be made available in summary form on the study website (hosted by the Sponsor)

(ii) Where individuals consented to have their results posted to them, we will provide summary measures of the primary study endpoints for that participant, in addition to a summary of the aggregate results.

7 STUDY INTERVENTION

7.1 DESCRIPTION

Daily oral mirtazapine 30 mg per day (nocte), or matched placebo, supplied by Syntro Pty Ltd, for 12 weeks.

All participants will be provided with a self-help booklet (*On Ice*) at the initiation of the active medication phase.

Both the trial medication and the *On Ice* brochure will be provided by the Sponsor.

7.2 JUSTIFICATION

Rationale for dosing regimen: The effective dose range for mirtazapine is 15 mg to 45 mg per day with 30 mg being considered the optimal dose. Higher dose ranges increasing dropout due to adverse effects.[33] Phase 2 trials for methamphetamine dependence found benefits with 30 mg/day at 12 weeks; effects on depression are evident by 8 weeks, with this being the median length of efficacy trials for depression.[33] The dose delivered at bedtime was well-tolerated and incurred no serious adverse reactions.

Justification for the control condition: There are no currently approved medications for methamphetamine use disorder, and therefore we have chosen a placebo control condition.

Complementary treatment: All participants will be provided with a minimal intervention (the self-help booklet, *On Ice*). This has been chosen rather than a treatment as usual (or other psychosocial treatment) treatment, due to the lack of readily available standardised evidence-based treatment options for methamphetamine use.

7.3 DOSING AND ADMINISTRATION

Intervention:

One tablet is to be swallowed each evening for a duration of 12 weeks (tablets are not to be chewed or crushed or injected). No more than one tablet is to be taken in one day.

Medication will be provided in three bottles of 35 tablets (28 days of medication + overage of 7 tablets to accommodate late assessments). A single bottle will be provided to the participant at the baseline assessment, at the week 4 assessment and at the week 8 assessment. These bottles are fitted with MEMS® Smartcaps to monitor medication adherence. The window period for adherence will be 24 hours starting from 3 am. A missed dose can be taken up until 3 am the next morning, but not thereafter.

Tapering dose:

At week 12, the tapering medication will be provided to the participant. This smaller bottle contains 28 yellow tablets (15 mg mirtazapine/placebo) and does not have a MEMS® Smartcap.

One tablet is to be swallowed each evening for 28 days (tablets are not to be chewed or crushed or injected). No more than one tablet is to be taken in one day.

Medication beyond the trial: The trial medication will not be provided beyond the duration of the trial. If participants wish to continue taking the trial medication, they can discuss this with their trial physician at the end of the study. Their trial physician can provide a referral to their treating physician to facilitate ongoing off-label prescribing of mirtazapine.

7.4 MEDICATION ACQUISITION, STORAGE AND HANDLING

Refer to the Pharmacy Manual for detailed instructions on pharmacy procedures.

7.4.1 FORMULATION, APPEARANCE, PACKAGING, AND LABELING

Intervention:

Appearance: Light pink, oval shaped, scored, film coated tablets[ǂ]

Formulation:

Mirtazapine: 30 mg mirtazapine + excipients[§] (including lactose monohydrate).

Placebo: Excipients[§] (including lactose monohydrate)

Packaging: 90 cc white square high density polyethylene (HDPE) square, with 38 mm neck (38-400 finish), 5.2 cm long (6.6 cm including neck) fitted with MEMS® Smartcaps.

Tapering dose:

Appearance: 15 mg mirtazapine/placebo: Pale yellow, oval shaped, scored, film coated tablets[ǂ]

Formulation:

Mirtazapine: 15 mg mirtazapine[ǂ] + excipients[§] (including lactose monohydrate).

Placebo: Excipients[§] (including lactose monohydrate)

Packaging: 20 cc HDPE bottles with regular lids.

Manufacturer:

Renaclinical Limited, United Kingdom. (Import, storage and distribution in Australia via Syntro Ptd Ltd.)

Labelling:

Pill bottles will be labelled with TGA compliant information. Pharmacy will add study ID number as per the randomisation schedule provided.

ǂ Dimensions, detailed information on markings and images can be found in the Investigator Brochure.

§See the Investigator's Brochure for full list of excipients.

7.4.2 PRODUCT STORAGE AND STABILITY

Store below 30° C. Store in original container. Refer to expiry date on label. Storage should be as per requirements for a Schedule 4 medication.

7.4.3 PRESCRIBING, DISPENSING AND RETURN OF UNUSED MEDICATION

Medication will be dispensed by the pharmacy on receipt of a script from the Trial Physician. This can be provided electronically.

The prescription should be for 30 mg of mirtazapine/placebo, 1 tablet daily for 35 days, and it needs to include two repeats (as medication is replenished at weeks 4 and 8, and an additional script for 15 mg mirtazapine/placebo, 1 tablet daily for 28 days.

Lost medication bottles will be replaced. An additional repeat for the 30 mg formulation may be included to cover lost medication bottles.

The Trial Researcher will be responsible for obtaining the trial medication from pharmacy and providing it to the participant. Alternatively, the medication may be couriered direct from pharmacy, or from the trial site, to the participant. Local procedures should be developed at each trial site that comply with local regulations and pharmacy requirements.

The Trial Researcher will be responsible for retrieving used medication bottles from participants and returning them to pharmacy. Medication bottles can be retrieved at study visits. Participants will be supplied with a prepaid envelope to return any unused medication at the end of the trial. Participants are reimbursed $10 per returned bottle.

7.4.4 ACQUISITION AND ACCOUNTABILITY

Medication will be shipped from Syntro Pty Ltd in Melbourne Australia to each of the trial site pharmacies. Contact information for pharmacy can be found in the appendix.

The trial site pharmacy will be responsible for the storage of the medication, dispensing of the trial medication, and allocating the condition (mirtazapine versus placebo) based on the study ID (noted on the trial medication prescription) according to the randomisation schedule provided by the Sponsor.

The medication bottles have a tear off tab which indicates whether they contain mirtazapine or placebo (see example in Figure 5). This tab is removed by the pharmacist prior to dispensing and placed in the Individual Log Sheet for the participant (appended to the Pharmacy Manual). The pharmacist fills the information required on both the tab and the bottle (i.e., bottle No. 1-4 and the patient ID).

Pharmacies must keep a log of all stock received, dispensed, returned and destroyed. Individual study drug dispensing logs can be done on the forms provided. Other pharmacy accountability logs can be done according to the pharmacy's standard operating procedures for disposal of medication.

At the end of the trial, unused trial medication will be disposed of according to the pharmacy's standard operating procedures for disposal of medication. Medication destruction should only be done on the instruction of the Sponsor and once all medication has been accounted for. MEMS® Smartcaps will be returned to the Sponsor.

A copy of all pharmacy log records maintained for the study should be provided to the research term at the close of the study. These pharmacy documents should be stored with the original trial records.

7.4.5 AUDITING RETURNED MEDICATION (PILL COUNT)

Pharmacy will undertake a pill count for returned medication bottles (recorded on the participant's Individual Log Sheet). The pill count must be completed prior to destruction of the medication.

Only the front side of the participant's Individual Log Sheet is to be viewed by the trial study personnel. This front sheet can be provided to the trial researchers/investigators for medication auditing purposes. Once the study has been completed and unblinding has occurred, the full file containing the Individual Log Sheets can be returned to the study investigators. The Sponsor will notify the pharmacy when this can occur.

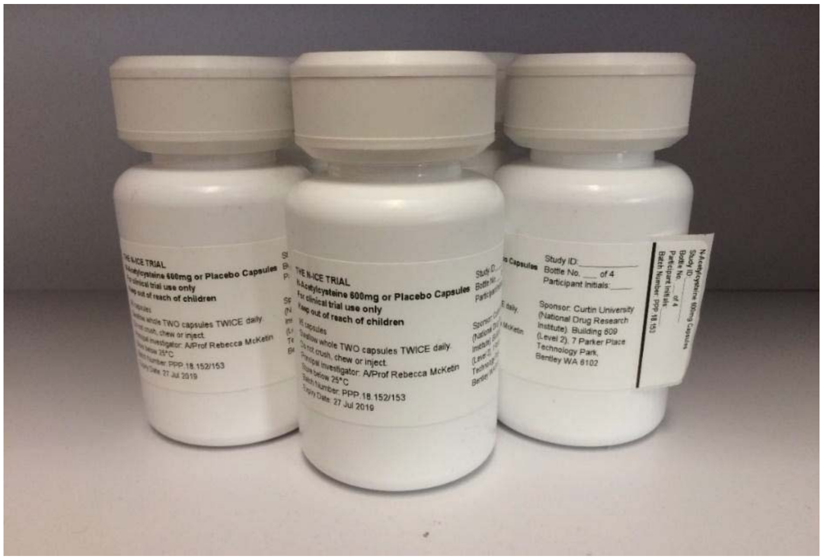

Figure 2. Example of medication bottles with tear-off tab and space for recording participant information

7.5 MEDICATION ADHERENCE

MEMS® will be used to obtain an objective measure of medication adherence. MEMS® data will also be used to counsel participants on adherence.

MEMS® Smartcaps will be fitted to the medication bottles used for the 12-week intervention medication phase. These Smartcaps record the time of bottle opening. Adherence is whether the pill bottle was opened once per day (with the day ending at 3 am), with no constraints on the time of opening within the 24 hour period, and no penalty for multiple openings (i.e., opening the bottle twice in one day would still be counted as adherent).

Activation of the MEMS SmartCap must be done on a desk-top computer using the MEMS® Reader prior to providing the medication bottle to the participant.

Once activated, field readings can be taken from the MEMS® Smartcaps using an NFC-enabled or OTG device (e.g., smartphone or tablet; compliant device to be provided by the Sponsor). This is done at assessments. Summary adherence data can be displayed in a calendar (Figure 2) which can be used by the Trial Researcher to counsel the participant on adherence.

The MEMS® Smartcaps also have an LCD display that shows the how many times the bottle has been opened each day (resets at 3 am daily). This helps participant to not to take more than one dose of medication per day.

Further details on the use of MEMS Smartcaps® can be found in Section 12.8.

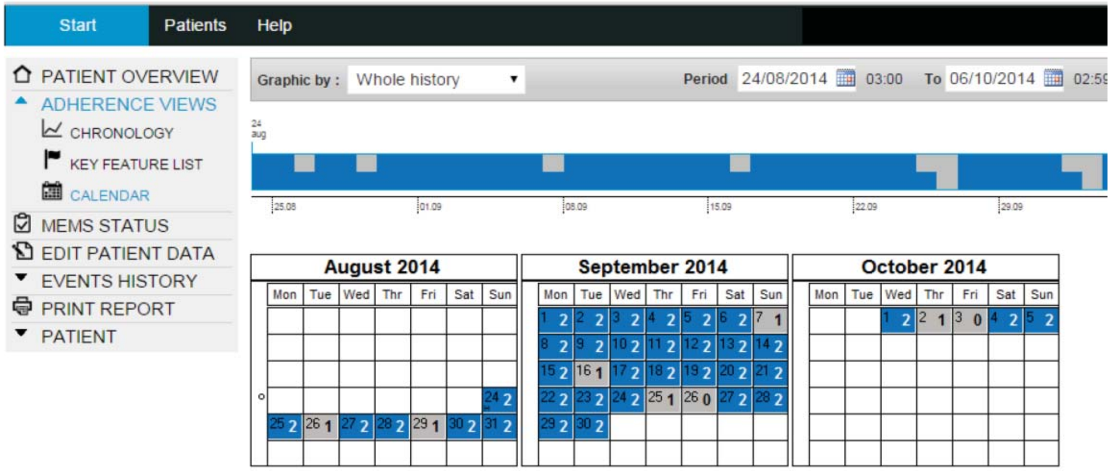

Figure 3. Example of MEMS® calendar view

7.6 RESCUE MEDICINE

Participants who report tolerability/adverse reactions may be reduced to a 15 mg dose by taking half of one tablet daily. No other rescue medications will be used.

8 SAFETY

8.1 GENERAL REQUIREMENTS

The safety procedures implemented in this trial are based on an assessment of the study risks (detailed in Section 9).

Risk associated with the medication will be managed by screening out participants who have contraindications for the trial medication or who, for other reasons, are at a high risk of experiencing adverse reactions. These risks have been listed in the Risk Assessment (Section 9) and have been incorporated into the inclusion and exclusion criteria. They are to be reviewed by the Trial Physician as part of the eligibility assessment.

Adverse events will be monitored and reported throughout the trial according to the guidelines in Section 8.3 and Section 8.4. Additionally, suicide risk is monitored at each assessment using the Columbia Suicide Severity Rating Scale Screener (CSSRS-S)[6,7] (see Section 6.4.2 for details).

The study will be overseen by an independent Data Safety and Monitoring Board who will review events and advise on safety.

Wherever possible, the Participant Information and Consent Form should be included in the patient medical record.

8.2 MEDICAL REVIEW

The medical care of participants will be provided by the trial site Trial Physician, who will be a licensed medical practitioner who is affiliated with the trial site health service.

All participants will undergo Medical Screening by the Trial Physician to confirm eligibility prior to being prescribed the trial medication.

A Final Medical Assessment will be conducted by the Trial Physician with all participants at least 4 days after the end of the tapering dose phase, and prior to the end of the study (see Section 6.6 for end of study definition).

Details of the Medical Screening can be found in Section 13.4 and details of the Final Medical Assessment can be found in section 13.10.

All AEs recoded on the eCRF at each assessment will be reviewed and signed off by the Trial Physician (see Section 8.4 for details of the medical review of AEs).

8.3 DEFINITIONS AND CODING OF ADVERSE EVENTS

8.3.1 DEFINITION OF ADVERSE EVENTS (AE)

An AE is any unfavourable and unintended sign (including an abnormal laboratory finding), symptom, or disease temporally associated with the use of the trial medication, whether or not considered to be related to the trial medication.

AEs should include only medical conditions that started after the participant initiated the trial medication. Medical conditions/diseases present before starting the trial medication are only considered AEs if they worsen after starting the trial medication.

Symptoms related to substance use intoxication or substance dependence (including withdrawal) should only be considered AEs if they exceed what would ordinarily be expected in this population (e.g., an unexpected exacerbation of existing dependence of drugs that requires referral for acute inpatient management).

8.3.2 DEFINITION OF SERIOUS ADVERSE EVENTS (SAE)

An SAE is any AE that:

- Results in death
- Is life threatening#
- Requires inpatient hospitalisation or results in prolongation of existing hospitalization (where hospitalization means a hospital admission, not an emergency department visit or outpatient visit),
- Results in persistent or significant disability/incapacity,
- Is a congenital anomaly/birth defect
- Is a medically important event or reaction

NOTE: The term “life-threatening” in the definition of “serious” refers to an event/reaction in which the patient was at risk of death at the time of the event/reaction; it does not refer to an event/reaction which hypothetically might have caused death if it were more severe.

Medical and scientific judgement should be exercised in deciding whether other situations should be considered serious, such as important medical events that might not be immediately life-threatening or result in death or hospitalisation, but might jeopardise the patient or might require intervention to prevent one of the other outcomes listed in the definition above. Examples of such events are intensive treatment in an emergency room or at home for allergic bronchospasm, blood dyscrasias or convulsions that do not result in hospitalisation (as outlined in the Guidelines on the reporting of adverse drug reactions by drug sponsors – Therapeutic Goods Administration -http://www.tga.gov.au/docs/html/adrguide.htm).

<u>An event will not be considered to be an SAE</u> if

- It involves hospitalisation for elective or pre-planned treatment for a pre-existing condition that has not worsened since the start of trial medication.
- It involves hospitalisation for treatment of drug dependence, unless that treatment is a result of a significant worsening of drug use since the start of the trial medication.

Should an event be considered so serious as to require unblinding of the participant, the Site Lead Investigator and site Trial Physician, and the Trial Coordinator and Coordinating Principal Investigator should be notified immediately so that adequate medical care can be provided to the participant. If unblinding is necessary (for any reason, including an SAE) the participant will be discontinued from the trial immediately. If this occurs all trial medication should be returned to the study team as promptly as possible.

In all other instances of an SAE, if the participant wants to continue on the trial the team will discuss the participants’ ongoing participation in the study. This may include discussion with the participants treating physician.

8.3.3 RATING THE SEVERITY OF THE ADVERSE EVENT

The severity of AEs will be graded according to the following guidelines.

Grade 1 Mild; asymptomatic or mild symptoms; clinical or diagnostic observations only; intervention not indicated.

Grade 2 Moderate; minimal, local or non-invasive intervention indicated; limiting age-appropriate instrumental activities for daily living*.

Grade 3 Severe or medically significant but not immediately life-threatening; hospitalization or prolongation of hospitalization indicated; disabling; limiting self-care activities**.

Grade 4 Life-threatening consequences; urgent intervention indicated.

Grade 5 Death related to AE.

A Semi-colon indicates 'or' within the description of the grade.

*Instrumental activities refer to preparing meals, shopping for groceries or clothes, using the telephone, managing money, etc.

**Self-care refers to bathing, dressing and undressing, feeding self, using the toilet, taking medications, and not bedridden.

8.3.4 CAUSALITY: RELATIONSHIP OF THE ADVERSE EVENT TO THE TRIAL MEDICATION

All AEs must have their relationship to study intervention reviewed by the Trial Physician, who examines and evaluates the AE based on temporal relationship and his/her clinical judgment. This will be recorded in the adverse event log in the eCRF. The degree of certainty about causality will be graded using the categories below.

Definitely Related – There is clear evidence to suggest a causal relationship, and other possible contributing factors can be ruled out. The clinical event, including an abnormal laboratory test result, occurs in a plausible time relationship to study intervention administration and cannot be explained by concurrent disease or other drugs or chemicals. The response to withdrawal of the study intervention (dechallenge) should be clinically plausible. The event must be pharmacologically or phenomenologically definitive, with use of a satisfactory rechallenge procedure if necessary.

Probably Related – There is evidence to suggest a causal relationship, and the influence of other factors is unlikely. The clinical event, including an abnormal laboratory test result, occurs within a reasonable time after administration of the study intervention, is unlikely to be attributed to concurrent disease or other drugs or chemicals, and follows a clinically reasonable response on withdrawal (dechallenge). Rechallenge information is not required to fulfil this definition.

Possibly Related – There is some evidence to suggest a causal relationship (e.g., the event occurred within a reasonable time after administration of the trial medication). However, other factors may have contributed to the event (e.g., the participant's clinical condition, other concomitant events). Although an AE may rate only as "possibly related" soon after discovery, it can be flagged as requiring more information and later be upgraded to "probably related" or "definitely related", as appropriate.

Unrelated – A clinical event, including an abnormal laboratory test result, whose temporal relationship to study intervention administration makes a causal relationship improbable (e.g., the event did not occur within a reasonable time after administration of the study intervention), and in which other drugs or chemicals or underlying disease provides plausible explanations (e.g., the participant's clinical condition, other concomitant treatments), or where the AE is completely independent of study intervention administration, and/or evidence exists that the event is definitely related to another etiology. The alternative probable etiology should be noted in the eCRF.

Where AEs are deemed probably or definitely related to the trial medication, they will be considered to be an adverse drug reaction (ADR).

8.3.5 ADVERSE REACTION

An adverse reaction is defined as an adverse event where there is at least a reasonable possibility that the trial medication caused the adverse event (i.e., either the Sponsor, investigator or reporter suspect a causal relationship between the trial medication and the adverse event).

8.3.6 EXPECTEDNESS OF THE ADVERSE REACTION

The study site Trial Physician, if necessary, in consultation with the trial investigators, will be responsible for determining whether an adverse reaction is expected or unexpected.

An expected adverse reaction is an adverse reaction known to be associated with the use of the medicine, as reflected in the Investigators Brochure or in this protocol.

An adverse reaction will be considered unexpected if the nature, severity, or frequency of the event is either not identified, or not consistent with the risk information for the trial medication provided in the Investigator's Brochure or in this protocol.

8.3.7 UNEXPECTED SERIOUS ADVERSE REACTION

An unexpected serious adverse reaction is a serious adverse event (see Section 8.3.2) which meets the definition for an adverse reaction (Section 8.3.5) and which is unexpected (Section 8.3.6), where unexpected means that its nature (that is, its specificity or outcome), severity or frequency is either not identified, or not consistent with the term or description used, in the product information or Investigator Brochure or in this protocol. Unexpected serious adverse reactions are also referred to as 'suspected unexpected serious adverse reactions' or SUSARs.

8.4 ASSESSING, REPORTING AND REVIEWING ADVERSE EVENTS

8.4.1 ASSESSMENT AND RECORDING OF ADVERSE EVENTS

The occurrence of AEs will be assessed at each assessment during the active medication phase (weeks 4, 8 and 12). Additional phone reviews of AEs will take place in week 2 and week 16. Assessments of AEs will be done by the Trial Researcher using the open-ended questions in the eCRF (i.e., unsolicited). AEs and SAEs may also include any additional events that come to the attention of trial staff during the trial.

All AEs will be recorded on the AE log in the participant's Case Report Form. Information to be collected includes event description, date of onset, severity, relationship with trial medication and whether or not the AE is a SAE. Each AE should be described and documented separately. Recurring/intermittent AEs require documentation of onset and duration of each episode.

8.4.2 MEDICAL REVIEW AND MONITORING OF ADVERSE EVENTS

<u>Review of AEs by the Trial Physician:</u>
All AEs recorded in the eCRF will be reviewed by the trial physician as soon as practical after the trial assessment. The Trial Physician will be responsible for:

- confirming the severity of the adverse event
- confirming relatedness to the trial medication
- confirming expectedness for adverse drug reactions

The Trial Physician may, at their discretion, request a medical assessment with the participant to review any AE. All possible SAEs and AEs that are rated as critical (grades 4 and 5) should be followed up with a medical assessment.

<u>Ongoing AEs during the trial:</u> Participants with an ongoing adverse event that is concerning them will be directed to contact the Trial Physician (or their treating physician) and not wait until the follow-up assessment. If a participant withdraws from the study early, the Trial Researcher will request that they attend a final medical assessment within the next week and to return unused trial medication. If the participant is experiencing a current AE at that time, he/she will be phoned one week later to follow up on that event.

Monitoring AEs at the end of the trial: AEs that are ongoing at the end of the trial medication phase, or after discontinuation of the trial medication (e.g., if the participant is withdrawn from the study), will be followed for at least 7 days. All AEs will be followed for outcome information until resolution or stabilisation.

All SAEs that are ongoing at the end of the trial medication phase, or after discontinuation of the trial medication (e.g., if the participant is withdrawn from the study), will be followed for at least 30 days. All SAEs will be followed until satisfactory resolution or until the site investigator deems the event to be chronic or the participant is stable.

8.4.3 ADVERSE EVENT REPORTING

A summary of AEs will be collated and reviewed at the monthly investigator meeting. All AEs will be reported at least biannually to the to the DSMB.

8.4.4 SERIOUS ADVERSE EVENT REPORTING

Reporting of SAEs within the trial team:

All potential SAEs should be reported immediately to the Trial Physician, the Lead Site Investigator, and the Principal Coordinating Investigator. This should be done regardless of whether the SAE is related to the trial medication and or whether the event was expected (i.e., including those listed on the Investigator's Brochure). **SAEs must be reported to the Sponsor within 24 hours of the SAE being reported to the trial team.**

In instances where the participant is unable to undergo an in-person medical review (e.g., hospitalised), the SAE form should be completed based on the information available.

Updates of the SAE form can and should be made promptly as further information becomes available, until the SAE is resolved. For reported deaths, terminal medical reports and any available autopsy reports should be provided to the Site Lead Investigator and the Coordinating Principal Investigator.

Both the immediate and follow-up reports should identify the participant by their unique Study ID.

Reporting of SAEs to the DSMB:

The Sponsor will notify the DSMB of all SAEs within 72 hours. The SAE Forms will be provided to the DSMB as they become available.

Reporting of SAEs to the HREC:

The Lead Site Investigator will notify the governing HREC of all SAEs and provide them with copies of the SAE Form(s) and related DSMB communication as required.

Reporting of Unexpected Serious Adverse Reactions to the TGA:

All unexpected serious adverse reactions must be reported to the TGA. The TGA must be notified if an adverse reaction is serious and unexpected, where there is a reasonable possibility that the event is related to the trial medication.

Unexpected serious adverse drug reactions can be reported to the TGA using the TGA's Australian Adverse Drugs Reactions System "blue card" (Report of suspected adverse reaction to medicines or vaccines). This notification is to be made by the Site Lead Investigator or the site's Trial Physician, with a copy forwarded to the Coordinating Principal Investigator. Alternatively, notification can be made by the Sponsor.

Fatal or life-threatening unexpected serious adverse reactions must be reported to the TGA within 7 days after being made aware of the event. Case Follow-up information should be reported within a further 8 calendar days (i.e., within 15 days of the trial team becoming aware of the event).

All other (i.e., non-fatal, non-life threatening) unexpected serious adverse reactions must be reported to the TGA within 15 days of the trial team being made aware of the event.

All unexpected serious adverse reactions must also be reported to the Coordinating Principal Investigator and Sponsor (via the Sponsor's delegate) as per the requirements for SAEs, and to the DSMB and HREC as per the requirements for SAEs.

All unexpected serious adverse reactions will be recoded on the UNSW Safety Monitoring Register.

Reporting AEs to participants:

Where SAEs are unexpected and related to the trial medication (i.e., unexpected serious adverse reactions), all participants will be notified either at their next assessment or, where they have completed the study, in writing using their preferred contact email/address. If required by the governing HREC, participants will be asked to consent to any additional procedures/risks or protocol changes that are made in response to the SAE.

8.5 SIGNIFICANT SAFETY ISSUES AND URGENT SAFETY MEASURES

8.5.1 SIGNIFICANT SAFETY ISSUES

A Significant Safety Issue (SSI) is a safety issue that could adversely affect participants' safety or materially impact the continued ethical acceptability or conduct of the trial. It is a new safety issue or validated signal considered by the Sponsor in relation to the trial medication that requires urgent attention of the TGA.

The Sponsor must notify the Therapeutic Goods Administration, Human Research Ethics Committee and all investigators of all significant safety issues within 72 hours calendar days of the sponsor instigating or being made aware of the issue.

Investigators must report all SSIs to the Sponsor's delegate within 24 hours. Follow-up details of the event must be provided to the Sponsor within 72 hours. Significant safety Issue reports must be recorded in the UNSW Safety Monitoring Register.

8.5.2 URGENT SAFETY MEASURES

An Urgent Safety Measure (USM) is a measure that is taken to eliminate an immediate hazard to a participant's health or safety. Significant safety issues that meet the definition of an urgent safety measure (i.e., where an urgent safety measure is required to be taken to eliminate an immediate hazard) must be classified as a significant safety issue requiring an urgent safety measure. The Therapeutic Goods Administration, Human Research Ethics Committee and all Investigators must be notified of any significant safety issues that meet the definition of an urgent safety measure within 72 hours. Examples include:

- a serious adverse event that could be associated with the trial procedures and that requires modification of the conduct of the trial.
- a patient population hazard, such as lack of efficacy of an intervention used for the treatment of a life-threatening disease.
- A temporary halting or termination of the trial for safety reasons

Investigators must report all USMs to the Sponsor's delegate within 24 hours. Follow-up details of the event must be provided to the Sponsor within 72 hours. Urgent Safety Measure reports must be recorded in the UNSW Safety Monitoring Register.

8.6 PROTOCOL DEVIATIONS

A protocol deviation is any noncompliance with the clinical trial protocol, the approved HREC protocol, or the guidelines for Good Clinical Practice (GCP) in Australia. The noncompliance may be either on the part of the participant, the investigator, or the study site staff. As a result of deviations, corrective actions are to be developed by the site and implemented promptly.

It is the responsibility of the Site Lead Investigator to use continuous vigilance to identify and report deviations to the Sponsor within no more than 30 working days of identification of the protocol deviation. All deviations must be documented (e.g., in the Incident Reporting Sheet) and reported to the approving HREC.

8.7 SERIOUS BREACHES OF GOOD CLINICAL PRACTICE

8.7.1 DFINITION OF A SERIOUS BREACH

A serious breach is defined as a breach of Good Clinical Practice, the clinical trial protocol, the clinical trial standard operating procedures, or the human ethics approval that is likely to affect to a significant degree the safety or rights of participants or the reliability and robustness of the data generated in the clinical trial. Examples of serious breaches include but are not limited to:

- Persistent or systematic non-compliance with the instructions for completing consent forms, safety monitoring forms and registers, case report forms or data collection tools that result in continued missed or incomplete data collection.
- Failure to record or report adverse events, serious adverse events, suspected unexpected serious adverse reactions, significant safety issues where urgent safety measures were implemented.
- Failure to conduct clinical trial procedures following the clinical trial delegation log.
- Widespread and uncontrolled use of protocol waivers affecting eligibility criteria, which leads to harm to trial subjects.
- Failure to report investigational medical product or device defects to the clinical trial sponsor or any relevant regulatory body.
- Failure to conduct research following the issued approvals, permits or licences by required laws, regulations, disciplinary standards, and UNSW policies relating to the responsible or safe conduct of research.
- Concealing or facilitating breaches (or potential breaches) of the Research Code by others.
- Researching without the requisite approvals, permits or licences required by laws, regulations, disciplinary standards, and UNSW policies related to the responsible or safe conduct of research.
- Failure to conduct research as approved by an ethics review body where that conduct leads to (or has the potential to) results in participant harms.
- Researching without ethics approval as required by the National Statement on Ethical Conduct in Human Research where that conduct leads to (or has the potential to) result in participant harms.
- Any breaches as outlined in the UNSW Research Misconduct Procedure or the Australian Code for responsible conduct of research that leads to (or has the potential to) result in participant harms.

8.7.2 REPORTING OF A SERIOUS BREACH

- A serious breach occurring at a participating site must be reported by the site Principal Investigator to the Coordinating Principal Investigator and Sponsor's Delegate within 7 days of the trial team becoming aware of the breach.
- The Coordinating Principal Investigator must review the serious breach, along with the clinical trial protocol, to develop a Corrective and Preventive Action (CAPA) that defines the steps to prevent the serious breach from reoccurring.

- The serious breach report and the CAPA must be provided to the approving HREC, and the UNSW sponsors delegate for review and approval.

8.7.3 REPORTING OF A SERIOUS BREACH BY THIRD PARTIES

- A Suspected Breach is a report judged by the reporter as a possible serious breach but has yet to be formally confirmed as a serious breach by the sponsor.
- A Suspected Breach form must be completed when a third party (e.g., individual/institution) wishes to report a suspected breach of Good Clinical Practice or the protocol and should be reported directly to the reviewing HREC without reporting through the sponsor.
- Recording of Protocol Deviation and Serious Breach Reports
- A register of protocol deviation and serious breach reports must be recorded. Written records and copies of documentation sent to the sponsor must be retained in the Investigator Site File.
- Copies of protocol deviation and serious breach reports must be recorded, written records and copies of documentation sent to the sponsor, referrals made to the HREC or establishing whether a breach of the Australian Code for Responsible conduct of research must be retained in the Master Site File.

8.7.4 REVIEW OF A PROTOCOL DEVIATION OR SERIOUS BREACH

- The UNSW Sponsor's Delegate will review reports to establish whether the event meets the definition of a protocol deviation or serious breach, to establish whether the proposed CAPA is appropriate and establish whether there is or will be an ongoing impact on the reliability and robustness of the data generated.
- The UNSW Sponsor's Delegate will seek advice from the approving HREC on the corrective and preventive actions.
- Protocol deviation or serious breach reports where a UNSW researcher, staff or student is responsible for the protocol deviation or the serious breach will be reviewed as per the UNSW Research Misconduct Procedure to establish whether a breach of the UNSW Research Code of Conduct has occurred.
- Protocol deviation or serious breach reports where the UNSW Sponsor's Delegate determines that site personnel are responsible for a protocol deviation or the serious breach will be referred onto their responsible institution for review under their Research Misconduct procedures to establish whether a breach of the Australian Research Code for the Responsible Conduct of Research has occurred.

8.8 UNANTICIPATED PROBLEMS

8.8.1 DEFINITION OF UNANTICIPATED PROBLEMS (UP)

Unanticipated problems include any incident, experience or outcome that involve risks to participants or others which meets all of the following criteria:

1. Unexpected in terms of nature, severity, or frequency given (a) the research procedures that are described in the protocol-related documents (including the Information Sheet and the approved Human Research Ethics proposal); and (b) the characteristics of the participant population being studied;
2. Related to participation in the research ("related" means there is a reasonable possibility that the incident, experience, or outcome may have been caused by the procedures involved in the research); and
3. Suggests that the research places participants or others at a greater risk of harm (including physical, psychological, economic, or social harm) than was previously known or recognised.

8.8.2 UNANTICIPATED PROBLEM (UP) REPORTING

The investigator will report UPs to the approving Human Research Ethics Committee (HREC), to Trial Coordinator and the Coordinating Principal Investigator. The report will include the following information:

(a) Protocol identifying information: protocol title and number, trial site, and the HREC project number;
(b) A detailed description of the event, incident, experience, or outcome;
(c) An explanation of the basis for determining that the event, incident, experience, or outcome represents an UP;
(d) A description of proposed changes to the protocol or other corrective actions that have been taken or are proposed in response to the UP.

To satisfy the requirement for prompt reporting, UPs will be reported using the following timeline:

- UPs that are SAEs will be reported to the HREC and to the Sponsor (Trial Coordinator and Coordinating Principal Investigator) within the time frame outlined for SAE reporting (see Section 8.4)
- Any other UP will be reported initially to the Sponsor (Trial Coordinator and Coordinating Principal Investigator) within 15 calendar days of the investigator becoming aware of the problem (e.g., by email, phone). This may include only points (a)-(c) above, together with proposed changes to the protocol or other corrective action if appropriate. A full report, with agreed changes to the protocol and/or other corrective action, will be submitted to the Sponsor and to the approving HREC within 30 days of the investigator becoming aware of the problem.
- All UPs should be logged in the local Incident Reporting Sheet for the study, and reported to the appropriate institutional officials at the study site (as required by an institution's reporting procedures for incidents) within 30 days of the investigator becoming aware of the incident.

8.8.3 REPORTING UNANTICIPATED PROBLEMS TO PARTICIPANTS

All study participants will be informed about any changes to the protocol, or risks associated with their participation, that arise from the UPs and may affect their participation in the study at their next assessment. If required by the approving HREC, participants will be asked to consent to additional procedures/risks or protocol changes.

Where the UP does not substantially change the risks involved with participation, or the study procedures relating to participation (e.g. if the UP is related to safety protocols for interviewers) the participant will not be actively informed of the UP.

8.9 EVENTS OF SPECIAL INTEREST

Other events that should be reported to the Sponsor (via the Coordinating Principal Investigator and the Trial Coordinator) include any reports of taking the medication via routes of administration other than by swallowing (e.g., injecting), participants taking the trial medication above the prescribed dose, diversion of medication (i.e., intentionally selling/distributing the medication to others), or unintended consumption of the medication by people other than the participant, including children.

8.10 MONITORING CONCOMITANT MEDICATIONS

Data on concomitant medications needs to be recorded in the Case Report Form (eCRF) at the eligibility assessment, the baseline assessment and at the week 4, 8, 12, and 20 assessments. Concomitant medications include all prescribed medications (prescribed by a licensed physician), over-the counter medications and supplements.

Concomitant medications must be reviewed each month to assess the ongoing eligibility of participants (e.g., ensure that they are not taking medications that increases the risk of adverse events), and to help understand the etiology of adverse events.

Concomitant medications should be reviewed in conjunction with data from the eCRF on illicit use of prescribed drugs. Consideration should also be given the use of other illicit drugs when determining the aetiology of adverse events.

8.11 MEDICATION DISCONTINUATION AND PARTICIPANT WITHDRAWAL

8.11.1 DISCONTINUATION OF THE TRIAL MEDICATION

The decision to discontinue a participant from medication will be made by the trial site Trial Physician in consultation with the study team.

Discontinuation from trial medication may occur for a specific period during the trial if this is necessary to monitor an adverse event or because the participant is unable to take trial medication (e.g., incarcerated, hospitalised, suspected pregnancy). Restarting trial medication will be on the recommendation of the Trial Physician.

Participants may be permanently discontinued from the trial medication if they:

- become pregnant during the trial;
- develop contraindications for mirtazapine that significantly increase their risk of adverse events (see Investigator Brochure for further details);
- are diagnosed with a medical condition during the trial that places them at significantly increased risk of adverse events; or,
- are unable to comply with the study protocol to an extent that significantly compromises their safety (e.g., unable to attend assessments and medical appointments).

The date of medication discontinuation and recommencement recorded on the eCRF are that nominated by the Trial Physician (irrespective of medication adherence by the trial participant).

Participants may choose to discontinue the trial medication (e.g., if they dislike side-effects). This is recorded on the eCRF under other reasons for discontinuation.

Participants who are permanently discontinued from the trial medication, or who choose to permanently discontinue the trial medication, must be referred for their final medical assessment at the time trial medication is ceased (rather than waiting until the end of the trial assessment period), with the final medical assessment being scheduled at least 4 days after and within 30 days of ceasing the trial medication. Ongoing AEs are to be monitored as per Section 8.4.

Discontinuation from trial medication does not necessarily mean discontinuation from the study. For participants who remain consented in the trial (i.e., have not withdrawn their consent to participate), remaining study procedures are to be completed as indicated by the study protocol.

8.11.2 DISCONTINUATION OF TRIAL MEDICATION DUE TO PREGNANCY

If a participant becomes pregnant during the study, or suspect that they are pregnant, trial medication should be discontinued, and the participant offered referral to other alcohol and other drug treatment services. In the case of suspected pregnancy, if it is confirmed within a reasonable period that the participant is not pregnant, trial medication can be resumed.

The procedure for medication discontinuation: As soon as the Trial Researcher becomes aware of the pregnancy, they should advise the participant that use of mirtazapine is contraindicated during pregnancy, and, that under the protocol, they should stop taking the medication. They will additionally inform the participant that they will seek the advice of the Trial Physician and notify to the participant as soon as practical to confirm the medication discontinuation and organise a follow-up medical appointment with the Trial Physician.

The Trial Researcher will seek immediate advice from the Trial Physician (within 24 hours, e.g., by phone call) to confirm the discontinuation of the medication and obtain advice regarding following up the participant (e.g., making a medical appointment to see the Trial Physician). The participant will then be recontacted by the study team (Trial Researcher or Trial Physician) to confirm the medication discontinuation and to schedule their final medical assessment. This confirmation of discontinuation should ideally be done on the same day, prior to their next scheduled dose of the trial medication.

Pregnant participants should be referred for their final medical assessment after medication discontinuation. All participants who become pregnant should be unblinded prior to their final medical assessment and informed of their condition allocation by the Trial Physician. Participants who were receiving mirtazapine (not placebo) should be counselled by the Trial Physician on the risks of mirtazapine during pregnancy. All participants who fall pregnant during the trial should be provided with referral for appropriate ongoing care. Ongoing AEs are to be monitored as per Section 8.4.

The Pregnancy Reporting Form should be completed with all information available at the time of the assessment. For participants who are receiving mirtazapine, consent should be sought from the participant to follow them up until the birth of the child (or termination) to obtain data on the pregnancy and neonatal outcomes.

Pregnancies must be reported to the Sponsor delegate within 24 hours and to the DSMB within 72 hours. The presiding ethics committee and governance bodies should be notified as required.

Participants who fall pregnant during the drug will be asked to contact the Sponsor if they experience any adverse outcomes related to their pregnancy (even if they withdraw their consent to continue participation).

8.11.3 PARTICIPANT WITHDRAWAL FROM THE STUDY

Participants are free to withdraw from participation in the study at any time.

An investigator may withdraw a participant from the study for the following reasons:

- The participant unable to complete study procedures (e.g., incarcerated, moved out of area)
- Significant study intervention non-compliance that compromises the participant's safety (e.g., refusing medical assessments, taking trial medication in a way that increases the risk of adverse events)
- Behaviour that poses a risk to the safety and well-being of either the participant or the trial personnel (e.g., harassment or intimidation)
- If the participant meets an exclusion criterion (either newly developed or not previously recognised) that precludes further study participation (e.g., pregnancy, development of a disease or condition that substantially increases the risk of adverse drug reactions).

The decision to withdraw a participant is at the discretion of the Lead Site Investigator and should be made in consultation with the Trial Physician, the Trial Researcher(s) and the Sponsor. The reason for withdrawal must be recorded on the eCRF.

Participants who withdraw from the study will not be followed up for further trial assessments. However, participants who are withdrawn from the study should still be followed up for their final medical assessment and to follow up on outstanding AEs, in so far as the participant consents for this to occur.

Participants who are withdrawn from the study will be referred to local alcohol and other drug treatment services. A summary of the participant's study results are to be sent to the participant at their designated address/email, if they have provided consent for this to occur.

Any participants who withdraw from the study will be asked to return their trial medication to the study team. They will be provided with a reply paid satchel for return of trial medication.

Withdrawal due to loss-to-follow-up: A participant will be considered withdrawn from the study due to loss to follow-up only if they fail to attend all study assessments after being randomised. The date of loss to follow-up should be recorded as the date of the participant's last assessment.

8.12 STUDY DISCONTINUATION

This study may be temporarily suspended or prematurely terminated if there is sufficient reasonable cause.

If the study is prematurely terminated or suspended, the Coordinating Principal Investigator will promptly inform investigators, study participants, the approving HRECs, the funder (NHMRC), and other relevant governance bodies, and will provide the reason(s) for the termination or suspension. Study participants will be contacted, as applicable, and be informed of changes to the study visit schedule.

Circumstances that may warrant termination or suspension include, but are not limited to:

- Determination of unexpected, significant, or unacceptable risk to participants
- Insufficient compliance to protocol requirements
- Data that are not sufficiently complete and/or evaluable
- Determination that the primary endpoint has been met

The study may resume once concerns about safety, protocol compliance, and data quality are addressed, and satisfy the Sponsor, investigators and the approving HRECs.

9 RISK/BENEFIT ASSESSMENT

9.1 KNOWN POTENTIAL RISKS

The two major risks to participants are breaches of participant confidentiality and medication risks, which are detailed below.

9.1.1 CONFIDENTIALITY OF PARTICIPANTS

Unauthorised disclosure of their confidential trial data, including the identity of trial participants, may harm the participant (e.g. affect their reputation, employment opportunities, child custody).

The following measures must be used to reduce the risk of unauthorized disclosure of confidential trial data:

- The personal details of trial participants must be kept confidential and only disclosed to a third party either with the participant's consent, or without the participant's consent as required by law.
- All study assessment data must be de-identified, and only linked to the participant's personal information by a unique study ID.
- The documentation linking the study ID to a participant's personal information must be stored securely and separately from deidentified study data, and must only be accessible to authorized trial researchers (i.e., those listed as having access to this data on the ethics approval).
- Confidential trial data must be transported and stored securely.
- Study unit record file data will be shared with third parties for the purpose of data analysis and research only in a de-identified format that ensures that no individual study participant can be identified.

In addition, trial assessments and all study procedures should be conducted in a manner that is discrete and respectful of the participant's privacy. See Sections 12 and 13 for more detail on study procedures.

9.1.2 MEDICATION RISKS

The information presented in this section is based on current product information for mirtazapine's use as an antidepressant. The adverse reactions observed in clinical trials are listed below. Further information on risks, cautions and special populations can be found in the Investigator Brochure.

- Common symptoms (occurring in more than 1/100 and fewer than 1/10) include increased appetite and weight gain, drowsiness/sedation (esp. during first few weeks of medication), amnesia, and generalized or local oedema.
- Less common symptoms (≤ 1 in 100) include dizziness and headache, elevations in serum transaminase activity.
- Rare events (≤ 1 in 1000) include granulocytopenia and agranulocytosis, mania, nightmares/vivid dreams, epileptic seizures, tremors, convulsions, myoclonus, paraesthesia, restless legs (hyperkinesia), orthostatic hypotension, exanthema, arthralgia and myalgia.

Note that it is difficult to know whether adverse outcomes are due to mirtazapine or the depression which it is being used to treat.

A detailed description of medication risks, and actions taken in the Tina trial to reduce these risks, can be found in Table 3.

Caution is needed in certain populations and in the context of certain comorbidities (see Table 4 for details).

Details regarding preclinical safety data and post marketing reports can be found in the Investigator Brochure.

Other considerations:

Discontinuation from medication: Abrupt discontinuation of mirtazapine can cause symptoms (e.g., dizziness, abnormal dreams, sensory disturbances [including paraesthesia and electric shock sensations], agitation, anxiety, fatigue, confusion, headache, tremor, nausea, vomiting and sweating or other symptoms) which may be of clinical significance. To reduce the occurrence and severity of any discontinuation symptoms, a tapering dose will be provided to participants (15 mg/day for 28 days) at the end of the medication intervention. Participants will undergo a final medical assessment after this tapering dose. If discontinuation symptoms are intolerable, dose titration will be managed by the Trial Physician on the basis of the patient's clinical response.

Dependence/Tolerance: Clinical trials have not revealed potential for dependence in terms of drug-seeking or misuse (which may result in extra-medical use or diversion). Data from MEMS® Smartcaps can be used to monitor overuse and study teams should be vigilant for signs of diversion.

Hostility and self-harm: The use of selective serotonin reuptake inhibitors (SSRIs) and other newer antidepressants has been accompanied by reports of agitation-type symptoms, including self-harm or harm to others (akathisia, agitation, disinhibition, emotional lability, hostility, aggression and depersonalization). These behaviours are also related to methamphetamine use and occur commonly amongst people dependent on the drug. Symptoms will be monitored through the trials adverse event reporting system, with specific monitoring of suicidality using the CSSRS-S (see Table 2). Medication may be discontinued if these symptoms arise or worsen during the trial.

Data specifically on mirtazapine use in methamphetamine use disorder
Only two randomised controlled trials have examined mirtazapine as a pharmacotherapy for methamphetamine.[24,25] Both reported expected adverse reactions to mirtazapine in a minority of participants (drowsiness and weight gain). Neither reported that other adverse events were elevated compared to placebo. Neither reported any serious adverse events related to mirtazapine. Data reported on medication discontinuation and study dropout was limited but did not suggest remarkable differences between mirtazapine and placebo. Further detail can be found in the Investigator Brochure.

Table 3. Risks associated with mirtazapine and actions used in the trial to mitigate each of these risks

Risk/contraindication	Description of risk	Action to mitigate risk in Tina Trial
Hypersensitivity to mirtazapine	Mirtazapine is contraindicated in patients who are known to be hypersensitive to the drug or any of its components.	1. People who have a history of adverse reactions to mirtazapine will be excluded.
Suicidality	Mirtazapine has been associated with increased risk of suicidal behaviour in people with depression. Meta-analyses have found this risk occurs in people under 25 years of age. People with a history of suicide-related events or those exhibiting a significant degree of suicidal ideation prior to commencement of treatment are known to be at greater risk of suicidal thoughts or suicide attempts.	1. People who have attempted suicide in the past year will be excluded.<br>2. We will monitor suicidality at each assessment using the Columbia Suicide Severity Risk Scale Screener (CSSRS-S). Participants who score 3 or more will be referred for a suicide assessment (see Suicide Protocol).<br>3. Participants will be advised of the risk of suicidality and to notify trial staff (and seek medical support) if they experience suicidal ideation.
Toxicity with MAOIs	Mirtazapine has been associated with serious, sometimes fatal, consequences if combined with mono-amino-oxidase inhibitors (MAOIs). This syndrome sometimes resembles serotonin toxicity. Therefore, REMERON RD® should not be used in combination with MAOIs (including the antibiotic linezolid, and the thiazine dye methylthioninium blue [methylene blue], which are less well-known examples of MAOIs) or within a minimum of 2 weeks of terminating treatment with MAOIs.	1. Exclude people who have taken MAOIs (including the antibiotic linezolid and the thiazine dye methylthioninium chloride, methylene blue) in the two weeks prior to starting the trial.<br>2. Participants who initiate MAOIs during the trial will be discontinued from the trial medication.<br>3. MAOIs will not be prescribed during the trial or within 2 weeks of ceasing the trial medication.<br>4. Participants will be advised not to take MAOIs during the trial or within two weeks after the participant has ceased the trial medication.
Serotonin Toxicity/Neuroleptic Malignant Syndrome	There is a rare risk of serotonin toxicity, particularly if mirtazapine is taken in combination with other serotonergic and/or neuroleptic/antipsychotic drugs. Serotonin toxicity is a potentially life-threatening condition characterized by clonus (spontaneous or inducible or ocular) with agitation or diaphoresis, tremor and hyperreflexia, hypertonia and body temperature > 38°C. The clinical manifestations of neuroleptic malignant syndrome often overlap with those of serotonin toxicity, including hyperthermia, hypertonia, altered mental status, and autonomic instability. In contrast to serotonin toxicity, patients with neuroleptic malignant syndrome may present with "lead pipe" muscle rigidity as well as hyporeflexia.	1. Exclude people who are currently being prescribed serotonergic antidepressants (tricyclic antidepressants, SSRIs, lithium) Ad-hoc extra-medical use will not result in exclusion.<br>2. We will advise participants against the use of serotonergic antidepressants (tricyclic antidepressants, lithium) or other serotonin-precursors (such as L-tryptophan, oxitriptan) and serotonergic drugs (triptans, tramadol, St. John's Wort) during the trial.<br>3. The use of antidepressant medication and antipsychotic medication (both prescribed and non-prescribed) will be monitored and recorded in the eCRF at each monthly assessment.

		4. Participants who initiate serotonergic antidepressants during the trial will be discontinued from the trial medication.<br>5. Participants who engage in ad-hoc use of these or other serotonergic agents will be counselled against their use, and may be discontinued from the trial medication (at the discretion of the trial physician) if non-compliant.<br>6. Trial physicians will use caution when prescribing antipsychotic agents and monitor for symptoms of serotonin toxicity/neuroleptic malignant syndrome.
Neutropenia, agranulocytosis	Bone marrow depression, usually presenting as granulocytopenia or agranulocytosis, occurs rarely. Symptoms mostly appear after 2-6 weeks of treatment. The bone marrow depression is, in general, reversible after termination of treatment, however, in very rare cases agranulocytosis can be fatal. For post-marketing experience of blood dyscrasias see: https://www.tga.gov.au/publication-issue/australian-adverse-drug-reactions-bulletin-vol-22-no-5	1. Participants will be warned about the symptoms of agranulocytosis and told to contact the trial staff or their physician if they experience any indication of infection, such as fever, chills, sore throat, or mucous membrane ulceration.<br>2. If a participant develops a sore throat, fever, stomatitis or other signs of infection, along with a low white blood cell count, the trial medication will be discontinued.
QT Prolongation / Torsade de Pointes	There have been cases of QT prolongation, torsades de pointes (TdeP), ventricular tachycardia, ventricular fibrillation, cardiac arrest, and sudden death, reported amongst people taking mirtazapine. The majority of cases involve concurrent overdose or occur in people with other risk factors for QT prolongation. Torsade de pointes may be asymptomatic or experienced by the patient as dizziness, palpitations, syncope, or seizures. If sustained, torsade de pointes can progress to ventricular fibrillation and sudden cardiac death. Caution should be exercised in people with known cardiovascular disease or family history of QT prolongation, or when used with other medicinal products thought to prolong the QTc interval.	1. Exclude participants with known cardiovascular disease or a family history of QT prolongation. Where indicated, a cardiogram may be performed to identify or exclude risk.
Overdose	Symptoms of overdose are usually mild and include depression of the central nervous system with disorientation and prolonged sedation, together with and mild hyper- or hypotension. However, there is a possibility of fatal overdose at doses beyond the therapeutic range, especially when other drugs are involved in the overdose. In these cases, QT prolongation and Torsade de Pointes have also been reported	1. Medication bottles contain only 35 tablets (28 doses + 7 days overage to cover for late appointments)<br>2. Participants will be told to only take one tablet per day, and the MEMS® Smartcaps have an LCD display that indicates how many doses have been taken that day, to avoid people taking more than one dose in a day.

		3. Adherence to medication will be monitored using the MEMS® Smartcaps, and data from the reading will show how many tablets the participant has taken in a day. If the participant has taken more than one tablet per day, they will be counselled on the risk of overdose. Information on significant non-adherence be referred to the Trial Physician and study team for review.<br>4. Participants will be warned of the increased risk of overdose if the prescribed study medication is combined with alcohol or other sedating drugs (benzodiazepines, opioids).
<u>Somnolence, sedative effects and impairment in mental and motor function</u>	Mirtazapine causes sleepiness (e.g., 54% of patients cf. 18% on placebo reported somnolence) and this is an important reason why people cease taking the medication. This sedation can lead to mental or motor impairment, which may in turn case accidents and injuries. This impairment is additive with alcohol and diazepam.	1. The trial medication will be taken prior to bedtime to reduce the impact of sedation on day-time activities<br>2. Participants will be warned about the potential sedating effects of the trial medication, including mental and motor impairment.<br>3. Participants will be cautioned about driving or operating machinery until they are reasonably certain that mirtazapine does not adversely affect their ability to engage in such activities.<br>4. Participants will be asked to avoid consumption of alcohol and other sedating drugs (including diazepam) while on the trial.
<u>Severe cutaneous adverse reactions</u>	Severe cutaneous adverse reactions (SCARs) including Stevens-Johnson syndrome (SJS), toxic epidermal necrolysis (TEN), drug reaction with eosinophilia and systemic symptoms (DRESS), bullous dermatitis and erythema multiforme, which can be life-threatening or fatal, have been reported in association with mirtazapine treatment. If signs and symptoms suggestive of these reactions appear, mirtazapine should be withdrawn immediately. If the patient has developed one of these reactions with the use of mirtazapine, treatment with mirtazapine must not be restarted in this patient at any time.	1. Any cutaneous symptoms reported by participants will be followed up by the Trial Physician.<br>2. If cutaneous reactions emerge, the participant will be discontinued from the trial medication

Table 4. Cautions with regard to specific conditions and populations, and actions taken in the trial to mitigate related risk

Population/condition	Description of risk	Action to mitigate risk in Tina Trial
Older adults and children	Mirtazapine is not indicated for people under 18 years of age; for people 65 years of age it may be associated with different safety and efficacy.	Exclude people aged under 18 years of age or over 65 years of age
Pregnancy or nursing	Safe use of mirtazapine during pregnancy or lactation has not been established.	1. Exclude participants who are pregnant or lactating<br>2. Exclude female participants who are unwilling or unable to avoid pregnancy<br>3. All female participants will do a pregnancy test prior to starting the trial to confirm they are not pregnant.<br>4. The Trial Physician will discuss effective contraceptive options with the participant and offer to prescribe contraception where indicated.<br>5. Participants who become pregnant during the trial will be discontinued from the trial medication.
Renal and hepatic impairment:	Increased plasma concentrations of mirtazapine can occur in patients with moderate and severe renal impairment and, to a lesser extent, in patients with hepatic impairment. Clinically significant alanine aminotransferase (ALT) elevations have occurred during short-term treatments in 2% of patients.	1. To mitigate this risk, people with moderately or severely compromised renal or liver function will be excluded.<br>2. Tests will be conducted to examine/monitor renal and liver function where indicated.
Lactose intolerance	Excipients include lactose. The medication should not be taken by people who have hereditary problems of galactose intolerance, the Lapp lactase deficiency or glucose-galactose malabsorption should not take the trial medication.	1. Exclude people with galactose intolerance, Lapp lactase deficiency or glucose-galactose malabsorption
Mania/hypomania	Caution should be used when prescribing mirtazapine to people with a history of mania/hypomania or other psychotic disorders because mirtazapine can exacerbate mania (reported in 0.2% of patients taking mirtazapine).	1. All participants will undergo a medical screen and prospective participants with a diagnosis of bipolar disorder will only be eligible if they are medically stable (e.g., receiving a mood stabiliser such as valproate or an atypical antipsychotic).<br>2. Symptom exacerbation will be monitored via routine adverse event reporting.
**Other conditions that require special consideration/caution:**		
Seizures	One case has been reported in clinical trials; no controlled studies have been done on patients with a history of seizures. There have been more cases of seizures reported post-marketing surveillance: https://www.tga.gov.au/publication-issue/australian-adverse-drug-reactions-bulletin-vol-22-no-5	1. Screening will identify participants who have a history of these conditions.<br>2. The trial medication will be discontinued if the condition develops or worsens during the trial.
Diabetes	Mirtazapine may alter glycaemia control.	

Hyponatremia	Hyponatremia has been reported to occur in people taking mirtazapine	
Acute narrow-angle glaucoma	Caution should be used in people with acute narrow-angle glaucoma and increased intra-ocular pressure	
Conditions that affect metabolism or hemodynamic	Clinical experience in this context is limited.	
Cardiovascular or cerebrovascular disease or people predisposed to hypotension	Mirtazapine has been associated with orthostatic hypotension and therefore should be used with caution in conditions that could be exacerbated by hypotension (history of myocardial infarction, angina or ischemic stroke) or conditions that would predispose people to hypotension (e.g., dehydration, hypovolemia and treatment with antihypertensive medication).	

9.2 KNOWN POTENTIAL BENEFITS

The potential benefit of mirtazapine is in reducing methamphetamine use amongst people who are dependent on the drug.

People who use methamphetamine have elevated mortality (all-cause standardised mortality ratio of 6·8, 95% CI 5·3 –8·8), this being related to suicide, drug poisoning, accidental injury, homicide and cardiovascular events.[34] They also experience a range of non-fatal harms, including elevated risk of cardiovascular and cerebrovascular events, mental health conditions (depression, psychosis and hostility), blood-borne viruses, and adverse neo-natal outcomes. Significant social harms include unemployment, crime, and social dislocation (e.g., relationship breakdowns, loss of child custody). Reductions in methamphetamine use, even in terms of reduced frequency of use, is associated with significant reductions in a range of health harms and adverse social outcomes.[35-37]

Two Phase 2 trials have been conducted of mirtazapine for methamphetamine use, both showing reduced methamphetamine use,[24,25] and one showing a reduction in depressive symptoms.[24] These trial provide preliminary evidence that mirtazapine may reduce significant harms attached to heavy methamphetamine use. However, trial participants were all male (mostly men who have sex with men), both trials were conducted in the USA, and participants were provided with concurrent psychological therapy.

It cannot be confirmed that mirtazapine will produce these same benefits when delivered in routine clinical care in Australia. We are conducting the Tina Trial to confirm whether these benefits occur when mirtazapine is delivered as part of routine clinical care in Australia, and to document safety when mirtazapine is used in this context.

10 STATISTICAL CONSIDERATIONS

10.1 SAMPLE SIZE DETERMINATION

Our sample size (N = 340) will detect a minimum rate ratio of 0.75 on our primary outcome (equivalent to a reduction from 24 days methamphetamine use in the past month to 18 days use) with 90% power (two-tailed test, p = 0.05). Reductions in methamphetamine use of this magnitude have produced clinically meaningful reductions in the risk of crime, violence and psychosis.[35-37] The sample size calculation was based on from the most recent Phase 2 randomised controlled trial of mirtazapine for methamphetamine dependence[24] and allows for up to 25% attrition.

10.2 POPULATIONS FOR ANALYSES

The intention to treat (ITT) dataset will include all participants who took at least one dose of the trial medication.

The modified ITT analysis dataset will include participants in the ITT dataset who had follow-up data at the relevant follow-up assessment for the nominated analysis.

The safety analysis dataset will include participants who took at least one dose of trial medication and also have safety data available (i.e., were not lost to follow-up after receiving their trial medication).

Per-protocol analysis dataset will include participants in the modified ITT analysis dataset and will exclude participants who were withdrawn from the study or discontinued the trial medication for reasons unrelated to related to adverse events. Any decisions about whether a level of protocol compliance and/or medication adherence is necessary to meet per protocol requirements will be agreed on by the study investigators prior to unblinding and documented in the statistical analysis plan.

10.3 STATISTICAL ANALYSES

10.3.1 GENERAL APPROACH

A Statistical Analysis Plan will be written prior to unblinding of the data which will confirm the final analysis strategy. This will include details of each planned analysis and methods of imputation.

Details of the endpoints to be used in each analysis can be found in Section 6.4. All tests will be two-sided with significance set at $p < .05$.

Descriptive statistics will be presented as the mean (standard deviation) for continuous parametric measures and median (inter-quartile range) for highly skewed measures. Categorical variables will be presented as a percentage per category.

For statistical analyses, the distribution of each outcome measure will be inspected, and a judgement will be made regarding whether the data conform to model assumptions (e.g. normality) and a decision will be made on the likely best model fit for analyses of each outcome prior to the unbinding of the data. Heterogeneity of the treatment effect between sites will be examined and considered in the model specification. The most parsimonious model option will be adopted for the final analysis.

10.3.2 ANALYSIS OF THE PRIMARY AND SECONDARY EFFICACY ENDPOINT(S)

The main analysis of both the primary and secondary endpoints will be based on unimputed data. Sensitivity analyses will use multiple imputations using chained equations to impute missing data. All tests will be two-sided with $p < 0.05$.

Primary endpoint: The analyses of the primary endpoint will be based on the ITT dataset, with the primary timepoint being week 12. The main effect of medication on methamphetamine use days will be tested using a mixed model with a group (placebo vs. mirtazapine) by time (baseline, week 4, week 8, week 12) interaction effect, with time entered as a factor variable, producing individual effect estimates for each time point and making no assumptions about linear changes over time.

Secondary endpoints:

The analyses of the secondary endpoints will be based on the modified ITT dataset.

The analyses of the secondary endpoint of methamphetamine negative oral fluid samples will be tested using a mixed model with a group contrast (placebo vs. mirtazapine) across all follow-up time points (weeks 4, 8 and 12) to obtain an average treatment effect. The outcome will be repeated measures of whether the participant had a negative oral fluid sample (no [0], yes [1]) at each timepoint.

The effect of mirtazapine on other secondary endpoints (depression, sleep, quality of life and HIV risk) will be tested with a mixed model using a group (placebo vs. mirtazapine) by time (baseline vs. follow-up [weeks 4, 8 and 12]) with time entered as a factor variable, producing individual effect estimates for each time point and making no assumptions about linear changes over time. The primary timepoint for these endpoints will be week 12.

Model choice, including distribution of the data (binomial, Poisson, negative binomial, linear etc.); covariates to be included in the analysis; procedures for imputation, estimation, and adjustment; and, the software used for the analysis, will be confirmed in the final Statistical Analysis Plan.

10.3.3 SAFETY ANALYSES

Data on AEs will be coded according to the Medical Dictionary for Regulatory Activities (MedDRA). AEs will be counted once only for a given participant. The event counted will be the event with the highest severity.

Safety analyses will report the number and percentage of participants reporting AEs and SAEs in each treatment condition, by System Organ Class (SOC); treatment conditions will be compared using a Pearson's Chi-Square test.

10.3.4 BASELINE DESCRIPTIVE STATISTICS

Baseline descriptive statistics will include the demographics of the sample (e.g., age, sex, employment status, education, prison history and drug treatment history), methamphetamine use history (e.g., days of use in the past 4 weeks, duration of use, route of administration), level of other drug use, and baseline data on all primary and secondary endpoints. These descriptive statistics will be compared between the treatment conditions using appropriate inferential statistics.

10.3.5 INTERIM ANALYSES

Interim analysis of the data is not planned.

10.3.6 SUBGROUP ANALYSES

Subgroup analyses may be undertaken for (a) men vs. women, and (b) co-occurring depression (based on PHQ-9 score of ≤ 10 vs. 10 or greater). Post-hoc power analysis will guide the interpretation of these analyses and whether they are viable.

10.3.7 TABULATION OF INDIVIDUAL PARTICIPANT DATA

Individual participant data will not be reported.

10.3.8 EXPLORATORY ANALYSES

Exploratory analyses (e.g., time-trends, per-protocol analysis) will be detailed in the Statistical Analysis Plan prior to unblinding. These will include a per-protocol analysis and analysis of time trends.

11 GOVERNANCE

11.1 ADMINISTRATION

11.1.1 COMPLIANCE

The clinical trial will be conducted in compliance with the following guidelines and documentation:

- ICH Guidelines for Good Clinical Practice (GCP)
- National Statement on Ethical Conduct in Human Research (National Statement)
- As approved by the Human Research Ethics Committee (HREC), the clinical trial protocol is responsible for monitoring the trial's conduct.
- The responsibilities set out by the UNSW Sponsors Delegate.
- The onsite or remote monitoring standard operating procedures as put in place by the clinical trial sponsor.

All sites will operate under the agreed Clinical Trial Research Agreements (or equivalent), which will be entered into with all trial sites prior to the start of the trial.

Each clinical site will develop local clinical procedures that comply with these requirements and perform internal quality management of study conduct, data and biological specimen collection, documentation and completion.

All trial site staff will be trained in Good Clinical Practice and all relevant aspects of the trial protocol and assessment procedures. All trial site staff will have appropriate qualifications for their role and be trained in trial procedures relevant to their role.

- The trial protocol will be in compliance with the "Standard Protocol Items: Recommendations for Interventions Trials" (SPIRIT) guidelines and will be registered prior to recruiting the first participant.

11.1.2 SPONSORSHIP ARRANGEMENT

The study operates under a co-Sponsor arrangement between organizations listed in the Multi-Institutional Agreement:

- University of New South Wales
- University of Wollongong
- Deakin University
- Monash University
- University of Sydney

The coordinating Sponsor (referred to as the Sponsor) is the University of New South Wales. Responsibilities for the conduct and oversight for the trial have been delegated from the University of New South Wales to the trial's Coordinating Principal Investigator (CPI; Rebecca McKetin). The CPI may delegate trial related responsibilities to the below listed Chief Investigator(s), including Chief and Associate Investigators, and any trial-related personnel.

All trial-related duties delegated by the Coordinating Principal Investigator, or the study Investigator(s) and trial-related personnel must only be delegated to those that are qualified by experience and training. Delegated responsibilities must be retained in the UNSW Clinical Trial Delegation Log. The UNSW Sponsor's Delegate is to be notified of the following:

- Protocol deviation reports outlined in the UNSW Research Misconduct Procedure.

- Any serious breach of Good Clinical Practice, the clinical trial protocol, the clinical trial standard operating procedures, or the human ethics approval that is likely to affect to a significant degree the safety or rights of participants or the reliability and robustness of the data generated in the clinical trial.
- Significant safety issues that are likely to (or have the potential to) affect to a significant degree the safety or rights of participants or the reliability and robustness of the data generated in the clinical trial.
- Urgent safety measures implemented to remove or prevent a significant safety issue.
- Safety reports relating to the continuation, suspension, or discontinuation of the clinical trial for safety reasons.
- Non-compliance with the protocol, SOPs, GCP, and applicable regulatory requirement(s) significantly affects or has the potential to affect human subject protection or reliability of trial results significantly.
- Participant complaints or concerns received concerning the conduct of the research.
- Significant modifications to the clinical trial that is likely to affect to a significant degree the safety or rights of participants or the reliability and robustness of the data generated in the clinical trial.
- Addition of participating trial sites, contractual arrangements at participating sites or modifications to legal agreements.
- The intention to conduct the trial in other countries.

All other trial sites operate under a Clinical Trial Research Agreement with the Sponsor or an equivalent arrangement.

11.1.3 STUDY PERSONNEL AND ROLES

Trial personnel, role, affiliations, and contact information for study personnel can be found in the 'General Information' table Section 2.

A Trial Management Group (TMG) may be appointed to oversee the operation of the trial as needed. This will include representation from the study investigator team.

Each site will maintain a delegation of authority outlining roles and responsibilities of all trial staff using the UNSW Clinical Trial Delegation and Responsibility Log (see Appendix) or an equivalent format approved by the Sponsor. A completed and signed delegation log must be provided to the Sponsor, and signed by the Sponsor's delegate, prior to initiating the recruitment of participants into the trial, and at any point during the trial when there are changes to the delegation of responsibility.

11.1.4 ETHICS

The study must be approved by the trial site Human Research Ethics Committee (HREC) and have received site specific governance approval prior to trial initiation. The implementation of all trial procedures must comply with the trial protocol, the approved HREC protocol, and with the guidelines for Good Clinical Practice (GCP) in Australia (*https://www.australianclinicaltrials.gov.au/researchers/good-clinical-practice-gcp-australia*). The study drug will be purchased from a provider with Good Manufacturing Practice certification.

11.1.5 CONSENT

All participants must provide informed consent prior to undertaking any of the trial procedures. Consent forms (or recordings of verbal consent) must be stored securely for all participants and be available for auditing at site monitoring visits. Consent may be obtained via any medium (e.g., written, verbal, online) that is approved by the governing HREC.

11.1.6 CONFIDENTIALITY AND PRIVACY

Participant confidentiality and privacy is strictly held in trust by the participating investigators, their staff, and the Sponsor and their interventions. This confidentiality is extended to cover testing of biological samples in addition to the clinical information relating to participants.

11.2 MONITORING

All trial sites will partake in clinical monitoring procedures to review safety, performance on key indicators, and compliance with the study protocol.

All aspects of the trial will be monitored by the Sponsor (UNSW) throughout the trial. Trial data will be monitored via the online REDCap database and weekly communication with trial sites. Data summaries will be reported to the investigator team monthly, to the DSMB 6-monthly, and to HRECs annually (Figure 4).

. Key indicators that will be documented and monitored as part of this reporting cycle include:

- Recruitment rates
- Follow-up rates
- AEs and SAEs
- Protocol deviations
- Medication discontinuations and withdrawal
- Unanticipated problems

All trial data will be reviewed for accuracy and completeness on a regular (e.g., monthly) basis. Any missing data or data anomalies will be communicated to the trial sites for clarification/resolution.

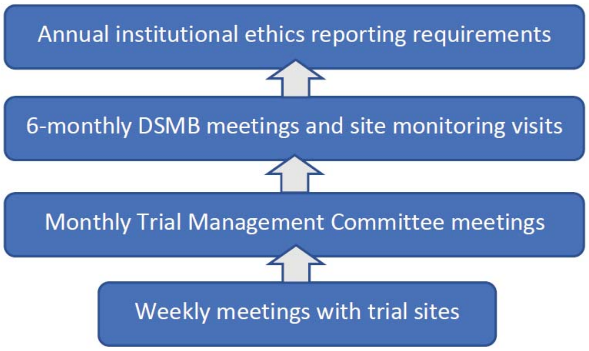

Figure 4. Reporting cycle used to monitor trial activity

11.2.1 PERFORMANCE TARGETS

Performance on three indicators will be monitored by the Sponsor (UNSW) throughout the trial via the online REDCap database.

- Recruitment of participants into the study (i.e., number of people randomised; see Section 12.2 for definition)
- Retention in the study (i.e., % of follow-up assessments completed for randomised participants; see Section 12.3 for definition)
- Protocol deviations (see Section 8.6 for definition)

Targets:

Recruitment: The target recruitment rate is approximately 1 participant per week at each site (for approximately 60 weeks over two years). For additional sites, the recruitment rate will be agreed in negotiation with the Sponsor. It is expected that

recruitment will be limited by staff taking leave and the close-down of facilities during holiday periods. Strategies to enhance recruitment can be found under study procedures in Section 12.2.

Retention: The target retention rate is 80%. Below 60% is less than adequate. Strategies to enhance follow-up can be found under study procedures in Section 12.3.

Protocol deviations: No more than 0.1 per participant (i.e., 1 per 10 participants).

Remedial actions:

Systems analysis will be used to identify the reasons for poor recruitment, retention or protocol deviations. Strategies will be implemented to improve recruitment, retention and/or reduce protocol deviations. Where recruitment and retention fail to recover to adequate levels, resources may be reallocated to other sites to maintain the overall study recruitment and retention targets. Where the investigator team decide that either the nature or the frequency of protocol deviations is not acceptable (e.g., poses a safety risk to participants, or compromises the integrity of the data) sites will be closed, and resources re-allocated to other sites.

11.2.2 SITE MONITORING

Site initiation visits will be conducted prior to recruitment to review the adequacy of site infrastructure and procedures and review all operating procedures.

Site monitoring visits will be conducted bi-annually during the trial by the Sponsor (UNSW). These will involve the review the original records to ensure completeness and compliance with the study protocol (e.g., completeness of consent forms and case report forms, and compliance with ALCOA; currency of GCP certification, HREC/governance approvals, insurance, trial master files; appropriate and secure storage of the trial site medication, patient data; budget and resources).

Site close-out visits will be conducted at the end of the trial to ensure that all trial activities have been completed, that trial materials and data are stored/returned/destroyed as appropriate, and that there is a management plan in place to handle post-trial matters (e.g., data access, complaints).

11.3 DATA SAFETY AND MONITORING BOARD

Safety oversight will be under the direction of an independent Data Safety and Monitoring Board (DSMB) composed of individuals with the appropriate expertise, including:

- Clinical experience in the alcohol and other drugs field
- Relevant clinical trial expertise
- Consumer and/or community representation
- Statistical expertise

Members of the DSMB will be independent from the study conduct and free of conflict of interest, and where this is not practical, measures will be taken to minimize the perceived conflict of interest. The DSMB will meet by teleconference at least semi-annually to assess safety and efficacy data on each arm of the study.

The DMSB will operate under the rules of an approved charter that will be written and reviewed by the DSMB. Each data element that the DSMB needs to assess will be clearly defined in the DSMB charter. The DSMB will provide its input to the study Sponsor.

11.4 DATA MANAGEMENT

11.4.1 DATA COLLECTION AND MANAGEMENT RESPONSIBILITIES

Data collection is the responsibility of the trial staff at the site under the supervision of the Site Lead Investigator.

The Site Lead Investigator is responsible for ensuring the accuracy, completeness, legibility, and timeliness of the data reported.

Trial data should be maintained electronically or in hard copy using the data templates provided. The Tina Trial will be using an electronic eCRF and data will be stored electronically on a REDCap database (built and maintained by the Sponsor). Further details on REDCap can be found in Section 12.10.

All hardcopy source documents should be completed in a neat, legible manner to ensure accurate interpretation of data.

The participant's Participant Information Sheet and Informed Consent Sheet should be stored on the participant's medical record, so that there is an independent record of their participation in the trial.

11.4.2 DATA STORAGE

All trial data, including the identity of participants in the trial, is to be treated confidentially, stored securely, and only disclosed to a third party with the participant's consent or as required by law.

- All identifiable participant information (e.g. consent forms, Participant Contact Form, Eligibility Form) is to be kept confidential and marked as such, stored securely (i.e. locked filing cabinet in a secure building), and only be accessible to authorised trial staff (i.e., those nominated on the HREC approval).
- All electronic files containing identifiable participant information must be password protected and stored on a secure computer directory (not on personal hard-drives, laptops or other portable devices) that is only accessible to the trial site investigators and other nominated trial staff.
- All trial assessment data (paper interview schedules and electronic databases, biological specimens) will be de-identified, marked clearly with the study ID, and re-identifiable only via the study ID. They will be stored in a separate secure location to protect the participants' identifiable information.
- When interviewing in the field, assessment materials should be kept on the interviewer's person at all times and kept in the lockable satchel provided by the Sponsor when not in use.
- All interview materials must be returned to the trial site filed as soon as practical after the interview
- Trial data will be reported in a way that does not identify any individual participant in the study.

11.4.3 DATA ACCESS

Access to confidential study data (e.g., participant names, dates of birth) is limited to study personnel who are listed on relevant ethics approvals as having access to that data (i.e., usually only local trial site personnel).

De-identified trial data will be accessible electronically by the Sponsor via REDCap. The REDCap database will include firewalls to restrict data access in accordance with institutional ethics requirements.

De-identified data may be provided to third parties for the purposes of research, provided that this is in accordance with ethics approvals.

The Sponsor, or other authorised representatives of the Sponsor, may inspect all documents and records required to be maintained by the investigator, including but not limited to, medical records (office, clinic, or hospital) and pharmacy records for the participants in this study. The clinical study site will permit access to such records.

11.4.4 FUTURE USE OF STORED SPECIMENS AND DATA

Biological specimens (oral fluid samples) collected during the study will be destroyed by the Victorian Institute of Forensic Medicine 3 months after a results report has been provided to the Sponsor.

Other trial data will be stored at the trial site where the research was conducted. De-identified data will be transmitted to the sponsor for the analysis of data.

The use of de-identified trial data by third parties will be permitted provided that written permission has been obtained by the Sponsor and that all relevant ethics approvals are upheld (e.g., maintaining the confidentiality of the participants, reporting the data in such a way that individuals and agencies are not identified) and that the source of the data and funding body is acknowledged.

Sharing of identifiable data requires the written permission of the sponsor and appropriate local ethics and governance approvals.

11.4.4.1 STUDY RECORDS RETENTION AND DESTRUCTION

Upon completion of the trial, all trial-related records will be stored securely for a period of 15 years. Electronic files and paper files will be destroyed in accordance with the trial site HREC requirements for confidential waste management. This will be the responsibility of the Coordinating Principal Investigator. Details can be found in the Research Data Management Plan (see Section 14, Related Documents).

11.5 PUBLICATION AND DATA SHARING POLICY

All publications will acknowledge the Medical Research Futures Fund grant No. 2007155 as the funder for the study.

Every attempt will be made to comply with the NHMRC's Policy on the Dissemination of Research Findings: *https://www.nhmrc.gov.au/grants-funding/policy/nhmrc-open-access-policy/*. Under this policy, the NHMRC requires that any publication arising from NHMRC supported research must be deposited into an open access institutional repository and/or made available in another open access format within a twelve month period from the date of publication.

No funding will be provided by the Sponsor, or under the associated Multi-Institutional Agreement, to support open access publication.

All publications will acknowledge the name of the trial (the Tina Trial) as the source of the study data.

The Sponsor will be notified of all publications arising from the project, and provided with an electronic copy of final published version of those papers.

Authorship on publications arising from the project will be decided using the guidelines provided by the International Committee of Medical Journal Editors: *http://www.icmje.org/recommendations/browse/roles-and-responsibilities/defining-the-role-of-authors-and-contributors.html*

Authors will provide accurate declarations of interest, as required for publication, in a timely manner.

The decision on papers written from the study will be jointly made by the trial investigators.

The Sponsor will be informed of the use of the data for additional analyses by individuals (including additional research being conducted by individuals other than the trial investigators). Access by third parties must comply with relevant institutional ethics approvals and this protocol.

11.6 CONFLICT OF INTEREST POLICY

The independence of this study from any actual or perceived influence, such as by the pharmaceutical industry, is critical. Therefore, any actual conflict of interest of persons who have a role in the design, conduct, analysis, publication, or any aspect of this trial must be disclosed and managed.

Furthermore, persons who have a perceived conflict of interest will be required to have such conflicts managed in a way that is appropriate to their participation in the design and conduct of this trial. The study Sponsor has established policies and procedures for all study group members to disclose all conflicts of interest and will establish a mechanism for the management of all reported dualities of interest.

12 STUDY PROCEDURES

12.1 INFORMED CONSENT PROCESS

All participants must be consented into the study prior to undertaking the eligibility assessment or any medical assessments. Written consent is required unless a procedure for verbal consent has been approved by the governing ethics committee.

Participants should be directed to the study website (*www.tinatrial.info*) to familiarize themselves with the study procedures prior to the consent process. Where possible, the participant should be emailed the consent form prior to the eligibility assessment. The consent form can also be placed on the local study website so prospective participants can download it.

The consent process must involve the following:

- Participants will have the consent form explained to them verbally, including that participation is voluntary and they are free to withdraw from the study at any time without prejudice
- Participants will be given time to read over the Participant Information Sheet and ask questions before providing informed consent
- Participants may choose to discuss the study with their family or friends prior to consenting (in which case the Eligibility Assessment should be rescheduled).
- Participants will be given a copy of the Participant Information Sheet and their signed Informed Consent Form for their records.

If individuals are intoxicated, or otherwise unfit to consent (e.g., acutely psychotic), the consent process and Eligibility Assessment should be postponed.

If, subsequent to the consent process, the Trial Physician deems the person incapable of providing informed consent (i.e., after they have signed the consent form) then the patient will be deemed ineligible.

At the initial medical assessment, the Trial Physician will answer any questions the participant has about the Participant Information Sheet, particularly the medical procedures involved in the trial. The Trial Physician will sign a declaration that this discussion has taken place. The participant will also sign a declaration that they have had the opportunity to discuss the trial procedures with the Trial Physician and have their questions answered.

Consent forms (or recordings of verbal consent) must be stored in a secure location separate to the de-identified study data.

Participants entering the trial may not have the capacity to readily appreciate the demands of the trial procedures. Participants should be questioned key study requirements to ensure their comprehension. For example, how often they need to come for assessments, who they should call if they experience any problems related to the trial medication, the voluntary nature of their participation, and what they should do if they wish to withdraw from the study.

If participants do not comprehend the study procedures, or are suspected to be intoxicated or cognitively impaired, this information should be noted in the Eligibility Form and flagged with the Trial Physician.

12.2 RECRUITMENT

12.2.1 DEFINITION OF RECRUITMENT

Recruitment is defined as the number of participants randomised, irrespective of whether they complete follow-up assessments.

12.2.2 TARGET RECRUITMENT RATE

The planned recruitment rate is 1 new participant per week at each site over two years (note this applies to the primary trial sites of Geelong, Wollongong, Brisbane and Perth; recruitment rates for any additional sites will be negotiated with the Sponsor). Recruitment should be tapered over the end-of-year break (December/January) to reduce the workflow during this period. Allowance has been made for and breaks in recruitment due to staff leave and illness. The planned cut-off date for participant recruitment is November 30, 2024, with study visits ending by June 1 2025.

12.2.3 RECRUITMENT METHODS

Participants will be recruited primarily by advertisements (placed in local newspapers, free press and social media), flyers placed in out-patient health clinics (e.g., needle and syringe programs, community health care centres, general practice) and other relevant locations (e.g., shopping bulletin boards, bus terminals), flyers distributed by existing participants and word-of-mouth. See “Participant advertisement.doc” for example text. Participants can also be referred from helplines, local primary health care services and other research studies.

Participants can also be referred from drug treatment services, however, people who are seeking treatment for their substance use from that service, should first be offered the treatment they are seeking, or referral to an alternative specialist alcohol and other drug treatment[1]. If the participant does not want to engage with available services, then they can be referred into the study.

Participants may be enrolled in other treatments for their substance use disorder, or for any other health condition, provided that they meet trial eligibility criteria. However, the following recruitment settings be avoided or because they are likely to conflict with the eligibility requirements for the trial:

- Inpatient settings: Because eligible participants must be actively using methamphetamine, recruitment from inpatient settings and abstinence-based drug treatments (e.g., residential rehabilitation) should be avoided.

---

[1] Drug treatment includes government and non-government agencies and private facilities providing alcohol and/or drug treatment services, including in-patient hospital facilities (e.g., withdrawal management services), residential and non-residential rehabilitation (e.g., therapeutic communities), community-based ambulatory services, and outpatient services and opioid agonist therapy. The following services are not included: services based in prisons and other correctional institutions; agencies that provide primarily accommodation or overnight stays such as ‘sobering-up shelters’ and ‘halfway houses’; agencies that provide services concerned primarily with health promotion; needle and syringe programs; agencies whose sole function is to provide prescribing and/or dosing of methadone; acute care and psychiatric hospitals, pharmacies, allied health professionals (including counsellors, psychologists and psychiatrists) providing general or non-alcohol and/or drug specialist treatment services.

- Psychiatric settings: Because the use of contraindicated medications (e.g., SSRIs) will be common, recruitment of participants from psychiatric settings should be avoided.
- Opioid agonist therapy: Because there is an elevated risk of overdose from combining mirtazapine with sedative drugs, the recruitment of participants from opioid agonist therapies should be carefully considered.

12.3 RETENTION

12.3.1 DEFINITION OF FOLLOW-UP

A participant will be considered lost to follow-up for a particular assessment if they fail to attend the assessment visit within the designated timeframe for that assessment (see Schedule of Activities, Section 6.3).

12.3.2 TARGET FOLLOW-UP RATE

The target follow-up rate is 80%. Missed appointments are not considered protocol violations. However, follow-up rates will be monitored as a key performance indicator. Good follow-up is required to ensure that the study is adequately powered and to minimize attrition bias.

12.3.3 FOLLOW-UP PROCEDURES

All participants must be followed up for each assessment regardless of whether they have missed previous assessments. They should be followed until the end of study date (see Section 6.6) unless they have been withdrawn from the study (i.e., withdrawn their consent to participate or been withdrawn from the study by the study investigators).

The following actions must be taken if a participant fails to return to the clinic for a required study visit:

- The Trial Researcher will make every effort to regain contact with the participant and schedule the subsequent assessment visit (as per the Schedule of Activities, Section 6.3) and counsel the participant on the importance of attending assessments.
- Attempts to contact the participant are to be documented in the eCRF. There is no limit to the number of contact attempts. However, good judgement should be exercised in situations where contact has been made and communications are not being returned (e.g., participant is not returning phone calls), or where contact is with significant others (e.g., phoning partners or parents).

12.3.4 STRATEGIES TO IMPROVE FOLLOW-UP

The following strategies to improve follow-up:

Compensation to participants: Adequate reimbursement for participation has been shown to increase retention in research trials and it has also been shown not to increase illicit drug use.[38] Participants will be reimbursed $50 per assessment to cover travel and other expenses related to participating in the trial.

Maintaining up-to-date and comprehensive contact information for participants: Detailed contact information will be obtained at baseline on the participant's Contact Form (CF). The CF needs to be updated at each follow-up to ensure that participants can be easily contacted to arrange assessments. Particular attention should be directed toward any contact information that could not be used successfully to locate the participant. Notes on the CF may include the days/time(s) and methods that are most suitable to contact the participant.

Assessment reminders: Text/phone call reminders will be sent to participants 1-3 days prior to the scheduled assessment time to improve appointment attendance. It is recommended that, where practical, assessments are scheduled at a regular time and venue throughout the trial.

Rapport: Good rapport with trial participants is essential to maximize retention. An example of techniques to build rapport can be found at https://counsellingtutor.com/basic-counselling-skills/rapport/

It is reasonable to purchase beverages (e.g., coffee, tea, soft drink) if conducting the interview in food outlets. No food should be purchased for the participant during the interview. If the participant wishes to purchase food they may do so, but they must pay for this out of their own pocket.

12.4 REIMBURSEMENT FOR PARTICIPATION

Participants will be reimbursed $50 per assessment to cover travel and other expenses related to participating in the trial.

This reimbursement will be provided for all scheduled study visits: the eligibility assessment, baseline assessment, week 4, 8 and 12 assessments, the week 20 final follow-up assessment, the two scheduled medical reviews with the Trial Physician (medical screening conducted as part of the Eligibility Assessment and the final medical review) and the phone reviews of AEs conducted by the Trial Researcher at week 2 and 16. Participants will not routinely be reimbursed for any other medical appointments or contacts made during the trial (e.g., ad-hoc medical appointments to review AEs). If participants require reimbursement of out-of-pocket expenses relating to these additional study visits, this can be approved by the Lead Site Investigator on a case-by-case basis.

Reimbursement will be in cash (wherever possible) and provided in full irrespective of whether the participant successfully completes the assessment or withdraws their consent to participate during the assessment. Reimbursement can be provided via electronic funds transfer where possible, or money order or other delivery system, where face-to-face contact is not practical.

12.5 MANAGING WORKFLOW AND ASSESSMENT WORKLOAD

The number of participants recruited per week must be limited around 1 per week to ensure the total number of assessments per week does not exceed the workload capacity of the Trial Researcher (estimated at around 10 assessments per week). If too many people are recruited in a short period, this capacity will be exceeded, leading to missed follow-ups, and increased missing data. Overburdening the trial researchers will also reduce rapport which can in turn increase study dropout.

The Assessment Workload Calculator provided can assist in forecasting interview loads. This workload is based on one new participant per week (as planned at major sites). This is a full-time workload for the Trial Researcher. Substantial time is required to conduct assessments and undertake related research activities (organise interviews, travel to and from interviews, data entry and management).

12.6 FIELD INTERVIEWING PROCEDURES

Assessments can be done in the field, and this should be done where practical because it improves retention. Each trial site should develop their own local safety protocol based on this protocol and the guidelines developed by Day et al.[39] (*Interviewer Safety In the Field.* National Drug and Alcohol Research Centre Technical Report No. 138, 2002). Guidance is provided below.

Assessment location: Assessment locations must be in a public place that is mutually agreed on by the participant and research assistant, and where both parties feel safe and comfortable. They can be conducted at the trial site, another health service or

similar, or at a location convenient to the participant. No assessments are to be conducted in private homes (including the participant's car or elsewhere on their premises). Interview venues should be well lit and not isolated. Both the interviewer and participant should be able to leave the environment easily (e.g., not be trapped in a corner).

Selection of field locations: If interviewing at a location convenient to a participant, choose a well-lit venue that is not isolated. Busy locations, such as shopping malls and fast-food outlets, are ideal because they have public amenities and the background noise makes it difficult for by-passers to hear the interview. If interviewing in a smaller outlet (e.g., café) ensure that the proprietors do not mind the interviews occurring in their venue. Explain that you are doing a research survey, and if asked, say that it is a health survey, focused on mental health and wellbeing. This helps protect the confidentiality of the participants. Often proprietors are happy to keep a tab for the study, which makes it easier to order beverages and pay at the end of the day when interviewing is complete. Health services can also be used for interviewing, provided that the safety protocols of the health service are followed, and that they do not conflict with the study Safety Protocol.

Transporting and storage of field interview materials: Completed assessment materials must be kept on the interviewer's person at all times in a lockable document bag. At the completion of field interviewing for a particular day, all interview materials should be returned to the Trial Site and stored securely as per the trial protocol. Interview materials must not be taken to the interviewer's home or stored in motor vehicles.

Interview materials: An interview pack should be prepared in advance for each assessment, including participant reimbursement. This will ensure that all assessments are completed in an organised and timely fashion, and there is minimal handling of cash during the interview (cash can be placed in an unmarked envelope).

Procedure for handling distressed participants: If a participant becomes visibly upset or agitated during the interview (e.g., if a question in the assessment process has caused them distress), pause the interview, acknowledge that the participant appears upset, and ask them if they would like to take a break from the assessment. Once the participant is calm, ask whether they wish to continue with the assessment before proceeding. Otherwise, suggest that the assessment can be continued at another time, and proceed to terminate the assessment and provide the participant with reimbursement. Provide the participant with referral to mental health support (e.g., ask them if they have someone they can talk to, and whether they'd like you to help them make an appointment with someone, including at the trial site, and make sure they leave the interview with a follow-up plan and with the Referral Sheet).

Procedures for handling termination of assessments: If it is not possible to complete an assessment due to a participant being intoxicated, agitated or distressed, the interview should be terminated. The participant should be politely informed that all of the assessments have been completed for the day. Skipping to the end of the interview to complete a few final questions can be a non-confrontational way to terminate the interview if the participant is keen to complete the assessment regardless of their state. The participant is to be reimbursed regardless of whether they have completed all assessments. All distressed participants should be offered a referral to a mental health support service. If the interview is be rescheduled, this should not be done while the participant is in an intoxicated or distressed/agitated state. The participant can be recontacted later to arrange the appointment.

Safety in the field: Personal security is important when interviewing in the field.

- Do not carry personal valuables to field interviews. Either leave them in the office or lock them in the car.
- Do not display money other than that owing to the participant and small denominations needed to purchase beverages.
- Park in a secure cark park or a well-lit public area or use public transport.
- Avoid travel after dark.
- Do not schedule interviews on weekends.
- Avoid scheduling interviews after hours during the weekdays; all interviewing should be completed, and field surveys returned to the office, by no later than 7 pm in the evening.

- Wear comfortable, practical and appropriately modest clothing (e.g., runners or boots, jeans rather than skirts). Do not wear valuable jewellery.
- If travelling to and from interviews using your personal car, try to park out of sight of the interview venue and avoid being seen returning to your car.
- If possible, familiarize yourself with the location beforehand to assess the suitability of the meeting place.

Further guidelines on safety are provided in Day et al.[39]

Monitoring field interviewers: Each trial site should develop their own local system to monitor the whereabouts of research staff who are interviewing in the field. This must include a log system to register when interviewers are in the field, and when they return, with a procedure for follow-up in the event that a field interviewer is not contactable. The following example can be used as a guide:

Example: Each field interviewer has a nominated monitor (e.g. Site Lead Investigator) who tracks their whereabouts. Field interviewers can record interviews in Microsoft Outlook as a meeting, inviting the monitor to attend the meeting. The monitor accepts the meeting, registering that they are accepting responsibility as the interviewer's monitor. To monitor the field interview process, the field interviewer sends text messages to the monitor at the following times (by way of example only):

(a) on departure from the trial site
(b) on arrival at the assessment venue
(c) on arrival of the participant
(d) at the completion of the assessment (and departure of the participant)
(e) when they are departing the assessment venue
(f) when they arrive back at the trial site

The monitor acknowledges receipt of each text message. If the field interviewer does not text at the expected time, this triggers a sequence of events until the field interviewer is located. For example: the monitor will initially text message the interviewer to check progress. If no response is received within a reasonable time frame (e.g., allowing for the interview to have been completed, taking into account contextual factors such as possible reception problems), the monitor will phone the interviewer. If the interviewer cannot be located by the end of the day, the monitor will use the interviewer's list of additional contact points to try to locate them (e.g., home phone number, phone numbers of their partner/family, as provided by the interviewer). If the interviewer still cannot be located within a reasonable timeframe, police should be contacted.

12.6.1 PROTECTION OF THE TRIAL PARTICIPANT'S IDENTITY DURING ASSESSMENTS:

Trial assessments will be conducted in a manner that is discrete and respectful of the participant's privacy. Contact with the participant's significant others will be discrete (e.g., referring to participation in a health study, without reference to methamphetamine per se) and will only disclose the participant's participation in the trial where the participant has provided their consent for this to occur.

All email and postal correspondence will include only generic address information (e.g., *tinatrial@unsw.edu.au*) and not refer to methamphetamine or other illicit drug use.

All voice mail messages will be generic (e.g., referring to the first name of the trial researcher and a health study) and not refer to methamphetamine use.

12.7 ASSIGNMENT OF STUDY IDENTIFIERS

There are two study codes assigned during the trial to de-identify records. These are unique to the participant. The first is the Screening ID, which is used to identify all participants who screened into the study. This is the primary code used to identify

people throughout the study. The second is the Study ID which is assigned at the point of randomisation. It determines whether the participant is allocated to the mirtazapine or the placebo group.

12.7.1 ASSIGNMENT OF THE SCREENING ID

Each person who completes the Screening Form must be assigned a Screening ID even if they are not eligible. This should be done for everyone who starts the Screening Form, even if they do not finish completing the screening process. However, it does not need to be done for general enquiries about the study.

The Screening ID will be used to identify the participant throughout the study. The screening ID consists of a letter (representing the study site: W = Wollongong, G = Geelong, B = Brisbane, P = Perth, O = all other locations) and three digits, which reflect the chronological order of screening (Figure 5).

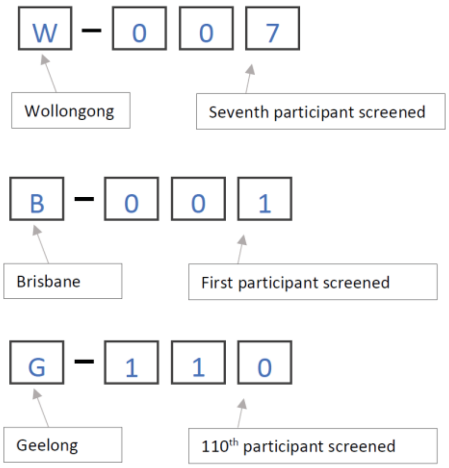

Figure 5. System for allocating Screening ID

12.7.2 ASSIGNMENT OF STUDY ID AND RANDOMISATION

The assignment of the Study ID will represent the point of randomisation.

Study IDs are to be derived by the Trial Researcher using the method below and recorded on the eCRF. The name of each participant is to be recorded against their Study ID in the password protected spreadsheet provided.

The Study ID is a 6 digit code. The first three digits reflect the randomisation strata, that being the

- study site (1 = Wollongong, 2 = Geelong, 3 = Brisbane, 4 = Perth , 5 = Other, 7 = Adelaide, 8 =Townsville)
- the sex of the participant (1 = male, 2 = female)
- whether they are depressed (1 = PHQ score of <10, 2 = PHQ score of 10+);

The second three digits is the sequence within each of these three strata. That is, sequence is allocated within each site, and numbered sequentially for (a) men who are depressed, (b) men who are not depressed, (c) women who are depressed, (d) women who are not depressed. For example, if a participant was the first participant to be randomised at Wollongong, they were male and depressed, their Study ID would be 111001. The second male who was recruited at Wollongong who was depressed would be 111002. If the third person to the randomised was a man who was not depressed, they would be 110001. If the fourth person to be randomised was a man who was depressed, they would be 111004. And so forth.

Below is a table that provides a series of examples for two sites. In this example there are only three participants allocated to each randomisation strata. The randomisation schedule allows for up to 60 participants to be allocated to each strata at each site.

**Site:**	**Gender:**	**Depression:**	**Sequence number in strata:**	**Study ID to be allocated:**
Wollongong	Male	PHQ-9 score < 10	1	111001
Wollongong	Male	PHQ-9 score < 10	2	111002
Wollongong	Male	PHQ-9 score < 10	3	111003
…				
Wollongong	Female	PHQ-9 score < 10	1	121001
Wollongong	Female	PHQ-9 score < 10	2	121002
Wollongong	Female	PHQ-9 score < 10	2	121003

Wollongong	Male	PHQ-9 score ≥10	1	112001
Wollongong	Male	PHQ-9 score ≥10	2	112002
Wollongong	Male	PHQ-9 score ≥10	3	112003
…				
Wollongong	Female	PHQ-9 score ≥10	1	1221001
Wollongong	Female	PHQ-9 score ≥10	2	1221002
Wollongong	Female	PHQ-9 score ≥10	3	1221003
…				
Geelong	Male	PHQ-9 score < 10	1	211001
…				
Geelong	Female	PHQ-9 score < 10	1	221001
Geelong	Female	PHQ-9 score < 10	2	221002
…				
Geelong	Female	PHQ-9 score ≥10	1	222003
…				
Brisbane	Male	PHQ-9 score < 10	1	311001
Brisbane	Male	PHQ-9 score < 10	2	311002
…				
Brisbane	Female	PHQ-9 score < 10	1	321001
Brisbane	Female	PHQ-9 score < 10	2	321002
…				
Brisbane	Male	PHQ-9 score ≥10	1	312001
Brisbane	Male	PHQ-9 score ≥10	2	312002
…				
Brisbane	Female	PHQ-9 score ≥10	1	322001
Brisbane	Female	PHQ-9 score ≥10	2	322002
Brisbane	Female	PHQ-9 score ≥10	3	322003
…				
Perth	Male	PHQ-9 score < 10	1	41001
Perth	Male	PHQ-9 score < 10	2	411002
…				
Perth	Female	PHQ-9 score < 10	1	421001
Perth	Female	PHQ-9 score < 10	2	421002
…				
Perth	Male	PHQ-9 score ≥10	1	412001
Perth	Male	PHQ-9 score ≥10	2	412002
…				
Perth	Female	PHQ-9 score ≥10	1	422001
Perth	Female	PHQ-9 score ≥10	2	422002
Perth	Female	PHQ-9 score ≥10	3	422003

12.8 ACTIVATION AND STOPPING OF MEMS SMARTCAPS

Each participant will receive three medication bottles that are fitted with a MEMS® SmartCap (to be provided to the participant at baseline, week 4 and week 8). The fourth bottle (provided at week 12) contains the tapering dose and does not have a MEMS® SmartCap.

Each medication bottle (MEMS® SmartCap) needs to be activated by the Trial Researcher before it is provided to the participant. Follow the MEMS instructions for assigning a new bottle to a participant (see MEMS Instructions provided by the Sponsor). Activation of the MEMS® SmartCap must be done on a desk-top computer or laptop using the MEMS® Reader (it cannot be done on a mobile device). The medication bottle must be linked to the participant in the MEMS software based on their study site and study ID. **Do not enter any personal identifiers into the MEMS software.**

Do not open the MEMS® SmartCap once it has been activated. This will register false recordings. Use the demonstration bottle provided to show participants how to use the bottle and to explain the LCD settings.

Once activated, field readings can be taken from the MEMS® Smartcaps using an NFC-enabled or OTG device (to be provided by the Sponsor). This should be done at assessments 4, 8 and 12 and on final bottle return.

At each assessment, the summary adherence data can be displayed in a calendar format by logging into the MEMS software (Figure 2). This information should be used by the Trial Researcher to counsel the participant on adherence. Review the information and discuss lapses in medication adherence. Emphasise the importance of taking the medication every day and help the participant come up with strategies to improve adherence (e.g., suggest setting text reminders, aligning medication taking with routine events, such as teeth brushing).

The participant should also be counselled on the LCD display on the MEMS® SmartCap (Figure 6) and to use this to make sure they only take one tablet per day.

After taking the final MEMS® recording, the MEMS® SmartCap needs to be stopped to prevent any false entries. This needs to be done by the Trial Researcher before returning the medication bottle to pharmacy.

Please follow the more detailed instructions on how to use MEMS® SmartCaps provided by the Sponsor.

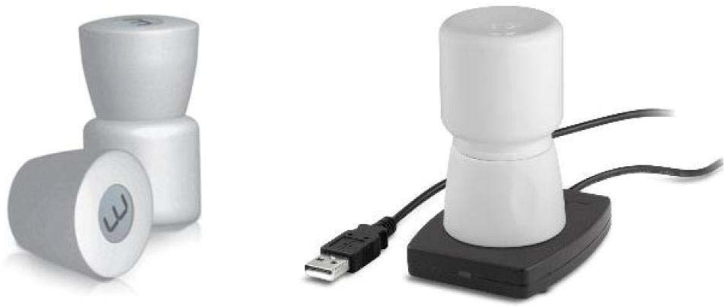

Figure 6. MEMS® SmartCap with LCD display and reader.

12.9 SALIVA COLLECTION AND HANDLING PROCEDURES

All trial researchers must be trained on the procedures for handling biological specimens in the trial. Trial researchers should also be familiarized with local site-based protocols for handling biological specimens. Local procedures should be developed that comply with both protocol requirements below and local site procedures.

Timing of the sample collection: The saliva sample needs to be taken at least 10 minutes after the participant has had anything to eat or drink. For this reason, it is best to take the saliva sample about 10 minutes into the interview, and prior to any beverages being provided to the participant. If the participant has recently drunk or eaten, then postpone the saliva sample until the end of the interview. The collection swab is not to be chewed or bitten. If the participant cannot produce sufficient saliva, ask them to drink a glass of water, wait 10 minutes, and try again.

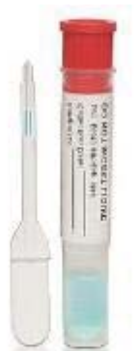

Figure 7. StatSure Saliva Sampler device

Procedure for collection: Disposable gloves must be worn when taking saliva samples. Saliva is collected using the StatSure Saliva Sampler Device (Figure 7). This will be provided by the Sponsor. Label the collection container with participant Study ID prior to testing. Take the saliva test according to the instructions provided (see Figure 8). The median collection time is 3.5 minutes. Once the saliva sample has been taken, the closed StatSure Saliva Sampler device must be placed in a padded biohazard bag (provided by the Sponsor), and this bag is to also be labelled with the participant's Study ID, the Trial Assessment Number and date.

Storage: Short-term storage at refrigerated temperature (4◦C) is recommended for saliva samples, or frozen (-20◦C or lower) during long-term storage (more than 2 weeks). Fridges must be reliable and backup options must be available in case of failure. The saliva tests need to be stored in the sealed, labelled biohazard bag.

Transport to testing facility: Saliva samples need to be transported to the Victorian Institute of Forensic Medicine (VIFM) for testing on a regular basis (e.g., batches of 50-100). Saliva samples need to be packed in the padded biohazard bags provided, and triple packed, as per the directions in the "Handling and Transport of Human Specimens". Transport packing boxes (that meet these triple packing requirements) will be provided by the Sponsor. The costs of testing will be covered by the Sponsor and the results will be delivered directly to the Sponsor.

For further details on the handling and transportation of the saliva samples, including courier addresses, refer to the document "Handling and Transport of Human Specimens".

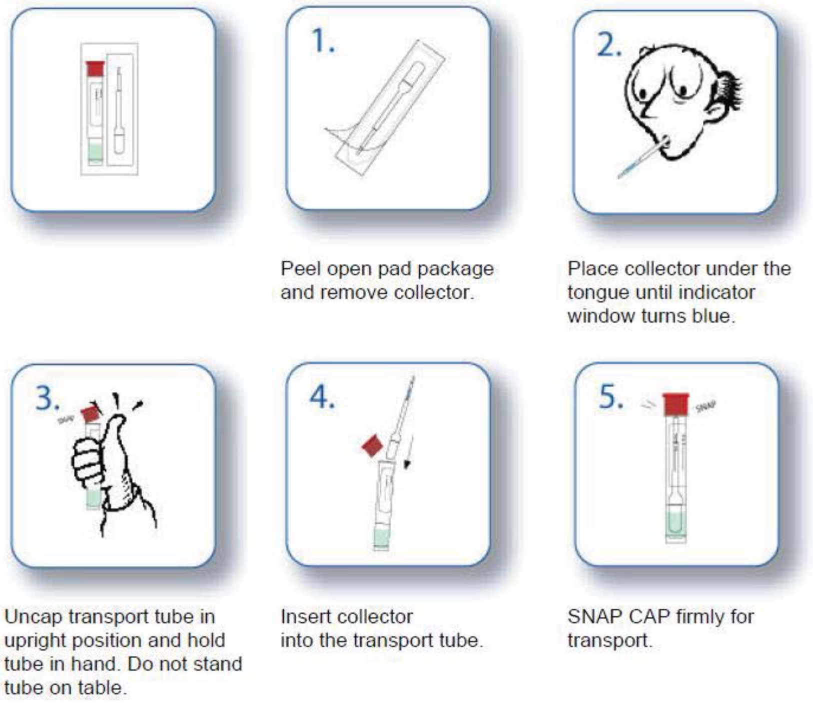

Figure 8. Instructions for the StatSure Saliva Sampler Device™

12.10 ELECTRONIC DATA MANAGEMENT AND REDCAP

The current study uses an electronic Case Report Form (eCRF). Data are maintained on REDCap and stored on the UNSW REDCap server.

Access to the REDCap database will be provided by the Sponsor. This will require obtaining a UNSW staff ID (zID), which will be arranged by the Sponsor. Each member of the study team who requires access to the REDCap database will be provided with their own log-in details. Study team members are to only use their own log-in details to access the database and must not provide their log-in details a third party (even if the third party is authorised to access the REDCap trial database). A two-factor authentication process will be used to maintain security of the database.

Training on the use of REDCap and the eCRF and data entry in REDCap will be provided by the Sponsor. Information on the UNSW REDCap system, including links to training videos, can also be found here: https://research.unsw.edu.au/redcap.

Assessment data should be entered directly into REDCap wherever possible. Offline data entry is available in REDCap using the REDCap Mobile App#, where internet is not available. In this case, data should be uploaded to the REDCap database as soon as practical after the interview.

Where this is not possible to complete the eCRF (e.g., REDCap field App not available/functioning), data may be entered into hard copy forms. All hard copy data should be entered into REDCap as soon as practical after the Assessment.

REDCap has built-in report functions for basic descriptive data. For more complex data analysis, data can be exported from REDCap into various formats that are readable by data analysis software (SPSS, Stata or SAS).

Sites will only have access to there own data during the study. The Sponsor will be able to access de-identified data from all sites. Identifiable data will be firewalled to allow access to only to authorised personnel (i.e., approved on the HREC).

Note. The REDCap Mobile App only needs to be used when there is no internet. It allows data to be entered when offline. The App syncs with the REDCap online database when internet connectivity is restored. Note that it is possible to use REDCap online, including from a tablet or mobile device, without needing to use the REDCap Mobile App provided an internet connection is available. If you are using a device without internet, consider purchasing a portable wireless router to act as an internet hotspot so you can enter data online in the field.

12.11 COVID RELATED PROCEDURES

Local site study procures should be developed to comply with both the trial site and the Institution's COVID-related procedures.

Provision for COVID-related restrictions in this trial include:

- Participant recruitment, screening, eligibility and consent may be done online or over the phone.
- Research assessments can be done by phone or videoconference (e.g., Zoom). Data can be directly entered into the eCRF via REDCap.
- Medication may be couriered to participants where pharmacies have the capacity to do this. No more than one medication bottle should be couriered to the participant at a time. The study team and Sponsor should be consulted in development of local procedures to courier medication to participants. The cost of couriering medication to participants will be covered by the Sponsor. However, arrangements for billing need to be made with the Sponsor apriori because funds for couriering medication are not included in the site budget.
- In the case of couriered medication, the baseline assessment should be scheduled as close to the receipt of medication as practical.
- Return of couriered medication bottled can be done by sending the participant a reply post envelope (costs covered by the sponsor).
- Medical screening and final medical review and any other medical assessments can be conducted by Telehealth.

Due to the need for collection of oral fluid samples, face-to-face assessments should still be conducted where possible, and particularly at the week 12 assessment, as this is the primary endpoint for the study.

13 DETAILS OF STUDY VISITS AND OTHER ASSESSMENTS

13.1 OVERVIEW

The timing of the clinical protocol procedures is provided earlier in Section 6. The following sections are presented in the chronological order of events in the study and are intended to provide additional detail on the content of each assessment.

For trial assessments, participants will be met by the Trial Researcher, either at the trial clinic, or at a suitable alternative location (see field interviewing, Section 12.6). Assessments can be done remotely (e.g., via Zoom or equivalent) where needed (e.g., due to COVID restrictions, see Section 12.11). However, it is good to do at least some face-to-face assessments where possible and practical to collect oral fluid samples (priority should be given to doing the eligibility assessment and the week 12 assessment face-to-face). Phone reviews of AEs at weeks 2 and 16, and the final 20-week assessment, which is also scheduled to be done by phone, can be done online (e.g., Zoom) if more convenient. Medical assessments can be done at the trial site clinic or via telehealth.

Participants should be reimbursed for each assessment as stipulated in Section 12.4.

13.2 PHONE SCREENING

Participants will initiate contact with the Trial Researcher who will brief them on the study and complete the Screening Form (usually by phone). The screening ID must be assigned for each person who completes the Screening Form (even if they are not eligible). See Section 12.7.1 for instructions on how to do this.

Potentially eligible participants need to be referred to the trial website (*www.tinatrial.info*) for further information on the study procedures, and, if possible, they should be provided with the participant information sheet prior to the eligibility assessment. This will give them time to read over the form and discuss it with their significant others.

For participants who are potentially eligible, the Trial Researcher will arrange a time to do the eligibility assessment. They will organise an appointment with the Trial Physician and the participant to do the medical screening (noting that the eCRF eligibility assessment forms must be completed before the medical screening, and these take up to 1.5 hours).

13.3 ELIGIBILITY ASSESSMENT (VISIT 1)

The Trial Researcher will consent the participant into the study prior to starting the eligibility assessment (see Section 12.1 for consent procedures).

The eligibility assessment involves completing the eCRF eligibility assessment forms, the pregnancy test (women only) and the DrugWipe test for methamphetamine.

Biological test results must be recorded on the eCRF. The Trial Researcher does not need to see the pregnancy test result, but they do need to see the DrugWipe test result. In both cases, the test equipment is to be handled and disposed in accordance with the trial site's standard procedures. See Section 12.9 and "*Handling and Transport of Human Specimens*" for further details regarding the handling of biological specimens under this study protocol.

The TLFB should be completed for the past 28 days.

All participants must be provided with an information pack containing the '*On Ice'* brochure, the Wallet Insert (with details of who to contact for unblinding in a medical emergency), and a list of local alcohol and other drug services, and emergency mental health services, through which they can receive support (i.e., the Referral Sheet).

At the end of the eligibility assessment, the Trial Researcher will share with the Trial Physician the Eligibility Form and any additional relevant screening and eligibility information obtained during the eligibility assessment.

13.4 MEDICAL SCREENING (VISIT 2)

The medical screen is part of the Eligibility Assessment. It should be conducted as soon as practical after visit 1.

The initial medical screen should involve:

- Reviewing and confirming patient eligibility against the study inclusion/exclusion criteria on the Eligibility Form, including:
    - discussing with the Trial Researcher the information on the Eligibility Form and any other relevant information arising from the eligibility assessment
    - reviewing the medical history of the patient to assist with determining eligibility and to identify any factors that may increase the risk of adverse events which require monitoring
- Instructing the participant about the risks of medication and likely adverse reactions. Trial Physicians are encouraged to explain dose-related effects of mirtazapine (i.e., sedation at lower doses vs. stimulation at higher doses).
- Recording the height and weight of the participant (self-report is acceptable where assessments are done via Telehealth)
- Reviewing contraception use by female participants, and offering effective contraception if needed
- Conducting other tests as needed to confirm eligibility and assess patient safety (including liver and renal function)#.

The Trial Physician should consult with the Trial Researcher after the medical assessment regarding eligibility and sign the Eligibility Form (see Section 13.5 for details).

The Trial Physician should confirm that they have discussed with the participant the trial medication, and any other medical procedures required for their participation in the trial, and answered any questions that they have, by signing the declaration at the bottom of the Eligibility Form. The participant can then sign the subsequent declaration that they have had this discussion. These declarations can be done either during the medical screening, or at a later date, but they must be done before the participant receives the study medication.

If the participant is not medically stable (e.g., recently begun receiving care/medication), or receiving other treatment that would preclude participation in the study, enrolment can be postponed until a more suitable time. In this case, the pregnancy test and medical screen will need to be repeated. If there is more than a 28-day delay between completing the initial eCRF eligibility assessments and the date of the rescheduled medical assessment, then the eCRF assessment forms need to be repeated/updated.

#The cost of liver and renal tests can be recouped from the Sponsor at the Medicare rate. No other test or treatment costs are covered.

13.5 RUN-IN PERIOD (CONFIRMATION OF ELIGIBILITY AND RANDOMISATION)

The run-in period is approximately one week between when a participant completes the eligibility assessment and when they attend their baseline assessment (when they receive the trial medication). It is designed to allow time for consultation between the Trial Physician and the trial research team to confirm eligibility (including waiting on test results). It is also designed to reduce the risk of randomizing participants who are unlikely to be re-contactable.

During the run-in period, the Trial Researcher needs to consult with the Trial Physician to confirm eligibility. Confirmation of eligibility will be based on the eCRF Eligibility Form. The Trial Physician is responsible for the “final decision” regarding eligibility on each of the eligibility criteria and the participant’s overall eligibility for the trial Both the Trial Researcher and the Trial

Physician need to sign the Eligibility Form. The Trial Physician and the participant need to sign the declarations at the bottom of the Eligibility Form to confirm that they have discussed the study medication and any other medical procedures that are required for trial participation (see Section 12.1 Informed Consent Process).

Once eligibility is confirmed, the Trial Researcher needs to re-contact the participant, using the information provided on the participant's Contact Form, to confirm they are still interested in participating. If so, the Trial Researcher can proceed to schedule the baseline assessment.

Randomisation should take place only after the participant has been re-contacted to confirm participation and once the baseline assessment has been scheduled.

The randomisation is done by the Trial Researcher by allocating a Study ID. Details on how to derive this 6-digit Study ID are can be found in Section 12.7.2. The Study ID must be recorded on the eCRF along with the date of randomisation.

Once the participant is randomised, the Trial Physician will prepare a prescription for the trial medication and provide this to the trial site pharmacy (this can be done via the Trial Researcher if a physical script needs to be delivered). The script must cite the Study ID (i.e., the six-digit numeric code described in Section 12.7.2) which will be provided to the Trial Physician by the Trial Researcher. A template for the script (if needed) is available in the Pharmacy Manual. Details on what should be included in the prescription and the prescribing process can be found in Section 7.4.3.

If participants cannot be recontacted within 4 weeks of the Eligibility Assessment, the eCRF Eligibility Assessment forms will need to be redone. Unless there are mitigating circumstances, the participant should be considered lost to follow-up. This should be recorded on the eCRF under reasons for not being randomised.

Ineligible participants: Participants who are not eligible, or who are no longer interested in participating, will be offered referral information for local alcohol and other drug services. This will involve providing the phone numbers and contact information for any relevant services over the phone, and offering to post, message or email to the participant a contact information sheet containing a list of services (i.e., the Referral Sheet).

13.6 BASELINE ASSESSMENT (VISIT 3)

The eCRF forms for the baseline assessment must be completed. The TLFB should be completed for the past 28 days. This can be filled using data from the eligibility assessment.

The first bottle of medication is to be provided to the participant along with instructions on medication adherence. The start date of medication must be recorded on the eCRF (this is the date of the baseline assessment if done face-to-face).

If the baseline assessment is done virtually (e.g., by Zoom) try to align the start date for the medication with the baseline assessment. For example, make sure the participant has received the couriered medication prior to the scheduled assessment date, and ask them not to start taking the medication until the baseline assessment, when you can give them instructions on how to take the medication and how to use the SmartCap.

Participants need to be counselled to:

- avoid driving or operating machinery if the medication makes them feel drowsy,
- to not take other anti-depressant drugs while on the trial, including SSRIs and St. John's Wort
- avoid taking other depressant drugs, including opioids and benzodiazepines
- contact the trial researcher and/or trial physician if they experience suicidal thoughts, or feel emotionally unstable (including experiencing angry outbursts or violent behaviour) or if they experience any other mental or physical effects from the medication that they are concerned about.

Participants need to be provided with the wallet insert, or equivalent electronic form, providing details of their trial participation, and instructed to show this card to their doctor if they receive any medical treatment during the trial.

13.7 WEEK 2 PHONE REVIEW (VISIT 4)

Participants should be phoned 2 weeks (+/- 1 week) after the baseline assessment to check whether they are experiencing any adverse reactions to the trial medication. This should be done by administering the AE form on the eCRF. If the participant is experiencing any adverse reactions to the trial medication, they should be asked whether they would like to see the Trial Physician, and a consultation with the Trial Physician should be organized accordingly. The AEs recorded at this phone interview should be forwarded to the Trial Physician as per the standard procedure for reporting and reviewing AEs (see Section 8.4). The Trial Physician may request an appointment to review the AEs reported.

13.8 WEEK 4, 8 AND 12 ASSESSMENTS (VISITS 5-7)

The eCRF forms for the relevant assessment need to be completed. The TLFB should be completed back to the previous interview. If the previous interview is more than 5 week (35 days earlier), complete only for the past 28 days.

The used medication bottle must be retrieved and replaced with a new medication bottle (at week 12 the tapering dose in the small bottle should be provided).

An oral fluid sample needs to be taken from the participant. See Section 12.9 for procedures.

The Contact Form should be updated.

At the week 12 assessment, the participant should be counselled about the possible withdrawal symptoms from stopping mirtazapine and be given a handout to take home outlining these symptoms. These can include dizziness, abnormal dreams, sensory disturbances [including paraesthesia and electric shock sensations], agitation, anxiety, fatigue, confusion, headache, tremor, nausea, vomiting and sweating. The participant must be advised to contact the Trial Researcher and/or Trial Physician if they are bothered by these or any other symptoms over the next four weeks. If they contact the study team with any concerns about withdrawal symptoms, they should be reviewed by the Trial Physician who will be responsible for managing withdrawal symptoms.

At week 12 (or sooner) participant should be provided with reply-paid envelope for return of any outstanding medication bottles.

At week 12, participants must be provided with the Referral Sheet, with details of relevant local agencies through which they can receive further support for their substance use or related issues.

13.9 WEEK 16 PHONE ASSESSMENT (VISIT 8)

The week 16 phone assessment involves the Trial Researcher administering the AE section of the eCRF by phone and organizing the time for the final medical assessment. The Contact Form can be updated as needed. The TLFB should be completed for the past 28 days.

13.10 FINAL MEDICAL ASSESSMENT (VISIT 9)

All participants will undergo a final medical assessment, carried out by the Trial Physician, after discontinuation of the trial medication.

The final medical assessment is to be scheduled for week 18, and should be undertaken at least 4 days after discontinuation of the trial medication tapering dose, and can be undertaken up to the end of study date (see 5.6 for definition).

The final medical assessment should involve:

- Review and sign-off of all outstanding AEs and SAEs
- Referral to the participant's treating physician for ongoing AEs
- A discussion with the participant about any mirtazapine withdrawal symptoms that they have been experiencing and providing medical management of these as needed.
- Discussing with the participant their preference for ongoing substance use treatment, and providing referrals to services as appropriate.
- Discussing with the participant whether they would like to continue to take mirtazapine, and options available for this, and providing referral to facilitate ongoing off-label prescription as appropriate.
- Conduct other tests to confirm patient safety as indicated

Note, participants who have been withdrawn or discontinued from the trial medication will be referred for their final medical assessment rather than waiting until the end of the trial. See section 8.11 for details.

13.11 WEEK 20 ASSESSMENT (VISIT 10)

The week 20 assessment will be conducted by the Trial Researcher via phone. The TLFB should be completed for the past 28 days. The purpose of the week 20 assessment is to assess the durability of any treatment benefits and whether stopping mirtazapine is associated with any withdrawal symptoms. It is also to check that the final medical review has been completed and all unused medication has been returned, and that the participant has referral information for ongoing substance use treatment.

The week 20 assessment forms on the eCRF need to be completed. The participant should be provided referral information to other services as needed. Appointments should be made with the Trial Physician to follow-up on any outstanding AEs as required.

13.12 MEETINGS BETWEEN THE TRIAL RESEARCHER AND TRIAL PHYSICIAN

Regular (e.g., usually weekly) meetings need to be scheduled between the Trial Researcher and Trial Physician during the trial to confirm eligibility of potential participants, review AEs, and to organise prescriptions for the trial medication. Participants need to be referred to the Trial Physician for a face-to-face assessment if they report severe AEs or SAEs at any of the assessments. Conversely, the Trial Physician may request a face-to-face assessment following review of a participant's AEs. See Section 8.4 for procedures to review and report on AEs.

14 RELATED DOCUMENTS

The following documentation is available from the Sponsor.

1. Participant Information and Consent Sheet
2. Verbal Consent Script
3. Investigator Brochure
4. Pharmacy Manual
5. Electronic Case Report Form template
6. Contact Form
7. Participant advertisement text
8. Referral Sheet
9. Serious Adverse Event Form
10. Incident Reporting Sheet
11. Wallet Insert
12. Handling and Transport of Human Specimens
13. MEMS Instructions
14. Suicide Protocol
15. Assessment Workload Calculator
16. Safety Protocol (for field interviewing procedures)
17. UNSW Safety Monitoring Register
18. Clinical Trials Delegation and Responsibility Log
19. Pregnancy Reporting Form
20. Pregnancy Participant Information and Consent Form
21. Research Data Management Plan

15 ABBREVIATIONS

AE	Adverse Event
ADR	Adverse Drug Reaction
AIS	Athens Insomnia Scale
ALCOA	Attributable, Legible, Contemporaneous, Original, Accurate
ASCEND	Advancing the health of people who use drugs: hepatitis C and drug dependence
CAPA	Corrective and Preventive Action
CF	Contact Form
CSSRS-S	Columbia Suicide Severity Rating Scale - Screener
DSMB	Data Safety and Monitoring Board
GAD-7	General Anxiety Disorder 7 Item Scale
HREC	Human Research Ethics Committee
eCRF	Electronic Case Report Form
EQ-5D	EuroQol 5D
GCP	Good Clinical Practice
GMP	Good Manufacturing Practice
HSU	Health Service Utilisation
ICMJE	International Committee of Medical Journal Editors
ITT	Intention-To-Treat
MedDRA	Medical Dictionary for Regulatory Activities
PGI	Patient Global Impression
PHQ-9	Patient Health Questionnaire – 9
RCT	Randomised Controlled Trial
SAE	Serious Adverse Event
SOC	System Organ Class
SPIRIT	Standard Protocol Items: Recommendations for Interventions Trials
SSI	Significant Safety Issue
SUSAR	Suspected Unexpected Serious Adverse Reaction (synonym for unexpected serious adverse reaction)
TGA	Therapeutic Goods Administration
TLFB	Timeline Followback
TSQM	Treatment Satisfaction Questionnaire for Medication
UP	Unanticipated Problem
USM	Urgent Safety Measure
WPAI-GH	Work Productivity and Activity Impairment Questionnaire: General Health

16 PROTOCOL AMENDMENT HISTORY

Version	Date	Description of Change	Brief Rationale
1.0	03/12/2021	N/A	Original HREC submission
2.0	13/01/2022	Changes made in response to feedback from the UNSW research office, including:<br><br>Addition of 'General Information' and 'Safety and Monitoring Contacts' tables (Section 2).<br><br>Removal of appendices containing contact information for trial personnel and pharmacy.<br><br>Reformatting/rewording to emphasise the reporting time-frame for reporting of SAEs (Section 8.4.4)<br><br>Addition of information in Significant Safety Issues and Urgent Safety Measures (Section 8.5).<br><br>Addition of text on Serious Breaches (Section 8.7).<br><br>Addition of reference to the UNSW Safety Monitoring Register and addition of this to the list of related documents in Section 14.<br><br>Addition of reference to the Clinical Trials Delegation and Responsibility Log under Section 11.1.3 and addition of this log to the list of related documents and to the appendix.<br><br>Removal of the table of delegated roles and responsibilities from Section 11.1.3.<br><br>Sections 8.3.5, 8.3.6 and 8.3.7 were added/amended to clarify the definitions of adverse reactions, their expectedness, and their relationship to suspected unexpected adverse reactions (SUSARs)	UNSW requirements
3.0	25/2/2022	Addition of Dr Frank Cordaro to protocol under Safety and Monitoring Contacts (Section 2, General Information)<br><br>Correction to email address of Juanita Koeijers (Section 2, General Information)<br><br>Addition of DSMB members (Section 2, General Information)<br><br>Addition of Perth pharmacy contact details (Section 2, General Information)	

		Clarification that 'direct-to-patient dispensing model' is not covered in this version of the protocol (Section 3 'Protocol Summary')<br><br>Correction to Table 1. Schedule of activities: removal of Medical review and AE form at baseline appointment<br><br>Addition 'Rescue Medication' text in Section 7.6<br><br>Change email address from tina@unsw.edu.au to tinatrial@unsw.edu.au<br><br>Addition of wording in section 13.4 'Medical Screening' to explain dose related medication effects<br><br>Correction of typographic errors on pages 81 and 43	
4.0	17/5/2022	<u>Protocol changes:</u><br><br>1. Addition of conditions related to lactose intolerance under exclusion criteria (Section 5.3)<br><br>2. Changed reimbursement for bottle return from $20 per bottle to $10 per bottle (Section 7.4.3)<br><br>3. Changed relatedness that requires reporting to the TGA to include only probably and definitely related (excluding possibly related) to match with Section 8.4.4 that there should be a reasonably possibility of being related before reporting to the TGA (Section 8.3.4)<br><br>4. Removed auditing of pill count by research staff (Section 7.4.5)<br><br>5. Added Medication Tolerability Scale in place of TSQM II at weeks 4 and 8 (Section 3, Table 1, Section 6.4.2,)<br><br>6. Changed the example provided of effect size equivalent from 25 to 20 days to 24 to 18 days to exactly match the detectable rate ratio of 0.75 (Section 10.1)<br><br><u>Administrative and other minor changes:</u>	1. Excipients of medication include lactose and may not be suitable for people with lactose intolerance.<br><br>2. Changed to cover reimbursement for return or each medication bottle, including return of tapering dose<br><br>3. Original reference was an error and inconsistent with other references to TGA reporting in the protocol.<br><br>4. Double counting of pill returns by study team was deemed unnecessary and logistically problematic<br><br>5. Cost of using TSQM II led research team to derive and validate their own scale for future use.<br><br>6. Example of effect size in days of use did not match the detectable rate ratio.

		Updated status of Human Research Ethics Committee status and contact information (Section 2)<br><br>Addition of DSMB members (Section 2)<br><br>Changed ‘trial management committee’ to ‘Trial Management Group’ to be consistent throughout document (Section 2 Section 11.1.3)<br><br>Amended all Quantisal references including diagrams to StatSure Saliva Collection Device (Section 12.9)<br><br>Corrected the name of DrugSwipe Test to DrugWipe Test (Section 13.3)<br><br>Clarified timeframe for the TLFB at each assessment (Sections 13.3, 13.6, 13.8, 13.9, 13.11)<br><br>Updated URL to training on rapport building (Section 12.3.4)<br><br>Clarified that participants should be asked to return study medication if they withdraw from the study, as per general end-of-study procedures (Section 8.11.3)<br><br>Other minor typographic and formatting changes	
5.0	06/09/2022	Protocol changes:	
		1. Addition of the Work productivity (WPA-GH) and the health service utilisation questions to the baseline interview.	To obtain baseline data on health service use to check for group balance in the outcomes analysis.
		2. Removal of item 2 of the HIV Risk Behaviour Scale (Table A1)	The similarity of items 2 and 3 caused confusion during piloting. Item 3 was considered more informative than item 2, so item 2 was removed.
		3. Possible timeframe of the week 16 AE review has been extended from ±7 days to ±21 days, provided that the assessment is conducted at least 7 days after the week 12 assessment.	To allow the AE review to occur from 7 days after the participant starts the tapering dose, and any time up to when the week 20 assessment is due.
		4. Addition of footnote under Table 2 to clarify when the Medication Tolerability Scale will be used in place of the TSQM-II.	To clarify the timepoints when the TSQM-II vs. the Medication Tolerability Scale will be administered.
		5. Addition of the GAD-7 Scale as a tertiary endpoint	To measure changes in anxiety as a tertiary endpoint, given mirtazapine’s anxiolytic effects.

		6. Addition of handout on mirtazapine withdrawal/side effects for participants at Week 12 visit (section 13.8)	Study medical expert advised that the tapering dose may increase adverse reactions to mirtazapine. The handout will be provided to participants to ensure they are aware of possible adverse reactions and know to contact the study team if they experience any adverse reactions from the tapering dose
		7. Inclusion criteria for past year DSM-IV diagnosis of methamphetamine dependence was changed to a past year DSM 5 diagnosis of moderate to severe methamphetamine use disorder	DSM 5 disorder was preferred as the current diagnostic system, but only moderate to severe levels were considered appropriate for the study.
		8. Clarified exclusion criteria around antidepressant use (Section 5.2) to explicitly exclude taking prescribed antidepressant medication use and any MAOIs in the past 14 days.	To remove ambiguity around inclusion/exclusion with regard to ad-hoc extra-medical use of prescribed antidepressants.
		9. Changed numbering on randomisation assignment to match final randomisation schedule	Randomisation program used different digits to identify strata to what was described in the protocol (Section 12.7)
		10. Added inclusion criteria for men being willing to avoid conceiving a child during the trial	Despite no evidence that mirtazapine effects male fertility, the investigator team felt that it would be safer for men to avoid conceiving during the study
		<u>Administrative and minor changes:</u>	
		Removal of HRECs for Brisbane and Geelong sites because HREC approval for these sites was received from Illawarra Shoalhaven Local Health District Drug and Alcohol Services and the University of Wollongong HREC)	
		Addition of additional trial physicians (Robert Lundin, Gina Sherry, Catherine Llewellyn) to protocol under Safety and Monitoring Contacts (Section 2, General Information)	
		Addition of Trial Researchers to under 'Other trial staff (Section 2, General information)	
		Update of HREC and CTN statuses in Human Research Ethics Committees, (Section 2, General Information)	
		Other minor formatting and typographic corrections and updates to contact information.	
6.0	20/1/2023	<u>Administrative changes:</u>	
		Addition of a new staff members, including a trial physician at the Brisbane site (Nikola Ognyenovits), trial staff at NDARC (Tayla Degan and Emma Zahra) and data programmer (Long Nguyen) and their contact details (Section 2, General Information)	
7.0	18/07/2023	<u>Protocol Changes</u>	

		Changes to Sections 12.1, 13.4 13.5 to ensure:<br>(a) The Trial Physician confirms participant eligibility.<br>(b) The Trial Physician signs the Eligibility Form to confirm that they have discussed the medically related aspects of the PISCF with the participant<br>(c) The Participant signs the Eligibility Form to confirm that they have discussed medically related aspects of the PISCF with the Trial Physician<br>The UNSW Clinical Trial Delegation Log (Appendix 2, Section 18.2) has been updated to reflect the above changes.	Response to internal audit conducted by Barwon Health which raised concerns about the consent procedure, specifically, that a medically qualified person confirmed eligibility and was involved in the consent process to answer questions that the participant had about medical procedures.
8.0	20/11/2023	<u>Protocol changes:</u>	
		Change to section 8.11.2 to update pregnancy discontinuation, unblinding and follow up procedures	Clarification of pregnancy related procedures as there have now been two pregnancies on the trial and it has become apparent that further guidelines to pregnancy & child follow up, discontinuation and unblinding were required.
		<u>Administrative Changes</u>	
		Addition of new staff member Ava Kontogiannis, Research Officer at the Wollongong site and removal of Tamsin Thomas who was previously in this position. Addition of new Adelaide site including Site Physician Will Liaw and Pharmacy Contact details (Section 2, General Information). Addition of new pregnancy reporting documentation to Section 14 'Related Documents'.	
9.0	11/03/2024	<u>Protocol Changes</u>	
		Section 3: Update of description of sites to include new sites in Adelaide and Townsville and extension of recruitment in Brisbane.<br>Section 12.7.2. Update of Study Site Codes to include new sites	Planned direct-to-patient dispensing model and recruitment through the ASCEND network has been abandoned. Recruitment is instead to continue through existing sites and addition of two new sites.
		<u>Administrative Changes</u>	
		Section 2: Addition of new staff members Rebecca Hyland and Vanessa Romeo (Research Officers at the Brisbane and Adelaide sites respectively) and Trial Physicians at Townsville (Ellie Holyoak, Brian Wu and Hayley Maher), Adelaide (Blaire Brewerton and Amelia Woods), and Geelong (Emily Tay and James Whittaker). Removal of trial staff Emma Hatton, Emma Zahra and Dr Robert Lundin, who are no longer working on the trial.<br>Update of Pharmacy contact details, Insurance details, and site details.	

10.0	17/06/2024	Administrative Changes	
		Section 2. Addition of new staff member Gift Kiden (Research Officer at the Townsville site) and Trial Physician at Geelong (Sarangi Nanayakkara). Reinstatement of trial physician Dr Robert Lundin as the Geelong Lead Trial Physician and removal of Dr Harry Hill as the Lead Trial Physician.	
11.0	23/7/2024	Administrative Changes	
		Section 2. Addition of new Trial Physician at the Geelong site (Antigone Branchflower).	
		Protocol Changes	
		Section 3. Modification of participant recruitment allocation at sites.	Recruitment is exceeding expectations at some primary sites, and some sites have indicated that they have capacity to recruit beyond the initial agreement of 60 participants. Extending recruitment at these sites will allow recruitment of trial participants until the agreed end of recruitment period.
12..0	01/10/2024	Administrative Changes	
		Section 2. Removal of section 'Other Trial Staff'. Addition of new staff members Alicia Neels (Geelong site pharmacist) and removal of staff Emily Tay and James Whittaker (Geelong Trial Physicians) and Moi Yap Thrift (Geelong pharmacist), addition of Steven Capell (Townsville Pharmacist). Removal of Gina Sherry, Perth trial Physician who has left the Perth team, update of contact details for David Goodman.<br>Update of lead HREC, removing ISLHD HREC and replacement with St Vincents Hospital, Sydney HREC.<br>Update of the Perth HREC removing the North Metropolitan Area Mental Health Service Human Research Ethics Committee (NMAMHS HREC) and updating to the WA Health Central HREC.<br>Update of UNSW Sponsor contact details. Removal of Ted Rohr and addition of Samela Husakovic.	HREC has advised that 'Other Staff' are not required to be listed on the trial protocol. As this list changes frequently, list has been removed and is housed in a separate document listing all personnel on the study (not verified by the HREC as per their direction). All study staff will remain listed on the site delegation log.<br>The North Metropolitan Area Mental Health Service Human Research Ethics Committee (NMAMHS HREC) has transferred its responsibility for the ethical oversight of the research projects that it has approved to the WA Health Central HREC.
13	13/08/2025	11.4.4.1 Update to procedure for study records retention and destruction. Research Data Management Plan has been added to the list of Related Documents (Section 14).	Research Data Management Plan has been updated. Therefore, the protocol has been amended to refer to this document.

17 REFERENCES

[1] Graves SM, Rafeyan R, Watts J, Napier TC. Mirtazapine, and mirtazapine-like compounds as possible pharmacotherapy for substance abuse disorders: evidence from the bench and the bedside. *Pharmacology & therapeutics* 2012;136:343-53

[2] Kroenke K, Spitzer RL, Williams JB. The PHQ-9: validity of a brief depression severity measure. *Journal of general internal medicine* 2001;16:606-13

[3] Soldatos CR, Dikeos DG, Paparrigopoulos TJ. Athens Insomnia Scale: validation of an instrument based on ICD-10 criteria. *Journal of psychosomatic research* 2000;48:555-60

[4] Darke S, Hall W, Wodak A, Heather N, Ward J. Development and validation of a multi-dimensional instrument for assessing outcome of treatment among opiate users: the Opiate Treatment Index. *Br J Addict* 1992;87:733-42

[5] Herdman M, Gudex C, Lloyd A, Janssen M, Kind P, Parkin D, Bonsel G, Badia X. Development and preliminary testing of the new five-level version of EQ-5D (EQ-5D-5L). *Quality of life research : an international journal of quality of life aspects of treatment, care and rehabilitation* 2011;20:1727-36

[6] Posner K, Brent D, Lucas C, Gould M, Stanley B, Brown G, Fisher P, Zelazny J, Burke A, Oquendo M, Mann J. Columbia-Suicide severity rating scale (C-SSRS) Version 6. New York, 2008. New York; 2008.

[7] Bjureberg J, Dahlin M, Carlborg A, Edberg H, Haglund A, Runeson B. Columbia-Suicide Severity Rating Scale Screen Version: initial screening for suicide risk in a psychiatric emergency department. *Psychological medicine* 2021:1-9

[8] NCCIH Clinical Research Toolbox. July 24 2018 2018. *https://nccih.nih.gov/grants/toolbox2018*).

[9] Reilly MC, Zbrozek AS, Dukes EM. The validity and reproducibility of a work productivity and activity impairment instrument. *PharmacoEconomics* 1993;4:353-65

[10] Mohebbi M, Dodd S, Dean OM, Berk M. Patient centric measures for a patient centric era: Agreement and convergent between ratings on The Patient Global Impression of Improvement (PGI-I) scale and the Clinical Global Impressions - Improvement (CGI-S) scale in bipolar and major depressive disorder. *European psychiatry : the journal of the Association of European Psychiatrists* 2018;53:17-22

[11] Atkinson MJ, Sinha A, Hass SL, Colman SS, Kumar RN, Brod M, Rowland CR. Validation of a general measure of treatment satisfaction, the Treatment Satisfaction Questionnaire for Medication (TSQM), using a national panel study of chronic disease. *Health and Quality of Life Outcomes* 2004;2:12-

[12] Degenhardt L, Larney S, Dobbins T, Chan G, Weier M, Roxburgh A, Hall W, McKetin R. Estimating the number of regular and dependent methamphetamine users in Australia, 2002-2014. *Medical Journal of Australia* In press

[13] Tait RJ, Whetton S, Shanahan M, Cartwright K, Ferrante A, Gray D, Kaye S, McKetin R, Pidd K, Ritter A, Roche A, Allsop S. Quantifying the societal cost of methamphetamine use to Australia. *The International journal on drug policy* 2018;62:30-6

[14] Farrell M, Martin NK, Stockings E, Borquez A, Cepeda JA, Degenhardt L, Ali R, Tran LT, Rehm J, Torrens M, Shoptaw S, McKetin R. Responding to global stimulant use: challenges and opportunities. *Lancet (London, England)* 2019;394:1652-67

[15] McKetin R, Najman J, Baker A, Lubman D, Dawe S, Ali R, Lee N, Mattick R, Mamun A. Evaluating the impact of community-based treatment options on methamphetamine use: findings from the Methamphetamine Treatment Evaluation Study (MATES). *Addiction* 2012;107:1998-2008

[16] McKetin R, Degenhardt L, Shanahan M, Baker AL, Lee NK, Lubman DI. Health service utilisation attributable to methamphetamine use in Australia: Patterns, predictors and national impact. *Drug Alcohol Rev* 2018;37:196-204

[17] Australian Institute of Health and Welfare. Alcohol and other drugs (AODT-NMDS) data cubes. Closed treatment episodes: client profile by drug of concern and treatment type by state/territory. 2013 ed; 2013.

[18] McKetin R, Voce A, Burns R, Quinn B. The Short Barriers Questionnaire (SBQ): validity, factor structure and correlates in an out-of-treatment sample of people dependent on methamphetamine. *Journal of Substance Abuse Treatment* In press

[19] McKetin R, Voce A, Burns R. Research into methamphetamine use in the Australian Capital Territory. Perth. Australia: National Drug Research Institute; 2017.

[20] Colfax G, Santos G-M, Chu P, Vittinghoff E, Pluddemann A, Kumar S, Hart C. Amphetamine-group substances and HIV. *Lancet (London, England)* 2010;376:458-74

[21] Brensilver M, Heinzerling KG, Shoptaw S. Pharmacotherapy of amphetamine-type stimulant dependence: An update. *Drug and Alcohol Review* 2013;32:449-60

[22] Trivedi MH, Walker R, Ling W, Dela Cruz A, Sharma G, Carmody T, Ghitza UE, Wahle A, Kim M, Shores-Wilson K, Sparenborg S, Coffin P, Schmitz J, Wiest K, Bart G, Sonne SC, Wakhlu S, Rush AJ, Nunes EV, Shoptaw S. Bupropion and Naltrexone in Methamphetamine Use Disorder. *The New England journal of medicine* 2021;384:140-53

[23] Ezard N, Dunlop A, Clifford B, Bruno R, Carr A, Bissaker A, Lintzeris N. Study protocol: A dose-escalating, phase-2 study of oral lisdexamfetamine in adults with methamphetamine dependence. *BMC Psychiatry* 2016;16

[24] Coffin PO, Santos GM, Hern J, Vittinghoff E, Walker JE, Matheson T, Santos D, Colfax G, Batki SL. Effects of Mirtazapine for Methamphetamine Use Disorder Among Cisgender Men and Transgender Women Who Have Sex With Men: A Placebo-Controlled Randomized Clinical Trial. *JAMA psychiatry* 2019

[25] Colfax GN, Santos GM, Das M, Santos DM, Matheson T, Gasper J, Shoptaw S, Vittinghoff E. Mirtazapine to reduce methamphetamine use: A randomized controlled trial. *Archives of General Psychiatry* 2011;68:1168-75

[26] Brensilver M, Heinzerling KG, Shoptaw S. Pharmacotherapy of amphetamine-type stimulant dependence: an update. *Drug Alcohol Rev* 2013;32:449-60

[27] McKetin R, Lubman DI, Lee NM, Ross JE, Slade TN. Major depression among methamphetamine users entering drug treatment programs. *Medical Journal of Australia* 2011;195

[28] Shoptaw S, Huber A, Peck J, Yang X, Liu J, Jeff D, Roll J, Shapiro B, Rotheram-Fuller E, Ling W. Randomized, placebo-controlled trial of sertraline and contingency management for the treatment of methamphetamine dependence. *Drug Alcohol Depend* 2006;85:12-8

[29] Fals-Stewart W, O'Farrell TJ, Freitas TT, McFarlin SK, Rutigliano P. The timeline followback reports of psychoactive substance use by drug-abusing patients: psychometric properties. *Journal of consulting and clinical psychology* 2000;68:134-44

[30] Trivedi MH, Greer TL, Potter JS, Grannemann BD, Nunes EV, Rethorst C, Warden D, Ring KM, Somoza E. Determining the primary endpoint for a stimulant abuse trial: lessons learned from STRIDE (CTN 0037). *The American journal of drug and alcohol abuse* 2011;37:339-49

[31] Fals-Stewart W, O'Farrell TJ, Freitas TT, McFarlin SK, Rutigliano P. The timeline followback reports of psychoactive substance use by drug-abusing patients: psychometric properties. *J Consult Clin Psychol* 2000;68:134-44

[32] Spitzer RL, Kroenke K, Williams JB, Löwe B. A brief measure for assessing generalized anxiety disorder: the GAD-7. *Arch Intern Med* 2006;166:1092-7

[33] Furukawa TA, Cipriani A, Cowen PJ, Leucht S, Egger M, Salanti G. Optimal dose of selective serotonin reuptake inhibitors, venlafaxine, and mirtazapine in major depression: a systematic review and dose-response meta-analysis. *The lancet Psychiatry* 2019;6:601-9

[34] Stockings E, Tran LT, Santo T, Jr., Peacock A, Larney S, Santomauro D, Farrell M, Degenhardt L. Mortality among people with regular or problematic use of amphetamines: a systematic review and meta-analysis. *Addiction* 2019;114:1738-50

[35] McKetin R, Lubman DI, Baker AL, Dawe S, Ali RL. Dose-related psychotic symptoms in chronic methamphetamine users: Evidence from a prospective longitudinal study. *JAMA psychiatry* 2013;70:319-24

[36] McKetin R, Lubman DI, Najman JM, Dawe S, Butterworth P, Baker AL. Does methamphetamine use increase violent behaviour? Evidence from a prospective longitudinal study. *Addiction* 2014;109:798-806

[37] McKetin R, Boden JM, Foulds JA, Najman JM, Ali R, Degenhardt L, Baker AL, Ross J, Farrell M, Weatherburn D. The contribution of methamphetamine use to crime: Evidence from Australian longitudinal data. *Drug Alcohol Depend* 2020;216:108262

[38] Festinger DS, Marlowe DB, Dugosh KL, Croft JR, Arabia PL. Higher Magnitude Cash Payments Improve Research Follow-up Rates Without Increasing Drug Use or Perceived Coercion. *Drug and alcohol dependence* 2008;96:128-35

[39] Day C, Topp L, Swift W, Kaye S, Breen C, Kimber J, Ross J, Dolan K. Interviewer Safety in the Drug and Alcohol Field: A Safety Protocol and Training Manual for Staff of the National Drug and Alcohol Research Centre: National Drug and Alcohol Research Centre, UNSW; 2002.

18 APPENDICES

18.1 APPENDIX 1: ADAPTATION OF THE HIV RISK BEHAVIOUR SCALE

The original HIV Risk Behaviour Scale from the Opiate Treatment Index has been revised for the Tina Trial. Outdated items on cleaning needles with bleach have been removed, as have items that contributed limited information on virus transmission risk (e.g., having condomless sex with regular partners), and, where possible, items have been combined (e.g., paid sex is now included under casual sex). The specific changes are shown in Table A1.

The resulting modified version of the HIV Risk Behaviour Scale has 6 items, and yields a score from 0 to 20, where higher scores reflect greater risk behaviour. Item 5 is intended to capture whether pre-exposure prophylaxis was used by the participant, so this can be taken into account in analyses as a potential protective factor for transmission of HIV. Item 5 is not included in the scoring of the scale.

Table A1. Details of how original HIV Risk Taking Behaviour Scale was modified for the Tina Trial

Original item	Revised item	Comments
1. How many times have you hit up (i.e. injected any drugs) in the last month?<br><br>Hasn't hit up (0)<br>Once a week or less (1)<br>More than once a week (but less than once a day) (2)<br>Once a day (3)<br>2-3 times a day (4)<br>More than 3 times a day (5)	1. How many times have you injected any drugs in the last month?<br><br>Hasn't injected (0)<br>Once a week or less (1)<br>More than once a week (but less than once a day) (2)<br>Once a day (3)<br>2-3 times a day (4)<br>More than 3 times a day (5)	Retained with modified wording
2. How many times in the last month have you used a needle after someone else had already used it?<br><br>No times (0)<br>One time (1)<br>Two times (2)<br>3-5 times (3)<br>6-10 times (4)<br>More than 10 times (5)		Removed due to confusion expressed by participants on piloting. Retained following question as more closely linked to risk of HIV transmission. .
3. How many different people have used a needle before you in the last month?<br><br>None (0)<br>One person (1)<br>Two people (2)<br>3-5 people (3)<br>6-10 people (4)<br>More than 10 people (5)	2. How many different people have used a needle before you in the last month?<br><br>None (0)<br>One person (1)<br>Two people (2)<br>3-5 people (3)<br>6-10 people (4)<br>More than 10 people (5)	Retained as is.

4. How many times in the last month has someone used a needle after you have used it?<br><br>No times (0)<br>One time (1)<br>Two times (2)<br>3-5 times (3)<br>6-10 times (4)<br>More than 10 times (5)		Dropped. Distributive sharing not directly related to participant's own HIV risk.
5. How often, in the last month, have you cleaned needles before re-using them?<br>Doesn't re-use (0)<br>Every time (1)<br>Often (2)<br>Sometimes (3)<br>Rarely (4)<br>Never (5)		Dropped. Bleach no longer recommended for prevention of HIV transmission.
6. Before using needles again, how often in the last month did you use bleach to clean them?<br><br>Doesn't re-use (0)<br>Every time (1)<br>Often (2)<br>Sometimes (3)<br>Rarely (4)<br>Never (5)		Dropped. Bleach no longer recommended for prevention of HIV transmission.

7. How many people, including clients, have you had sex with in the last month?<br><br>None (0)<br>One person (1)<br>Two people (2)<br>3-5 people (3)<br>6-10 people (4)<br>More than 10 people (5)	3. How many different people have you had sex# with in the last month?<br><br>None (0)<br>One person (1)<br>Two people (2)<br>3-5 people (3)<br>6-10 people (4)<br>More than 10 people (5)<br><br>#Include clients; include only penetrative sex	Retained with modified wording and clarification added.
8. How often have you used condoms when having sex with your regular partner(s) in the last month?<br><br>No reg. partner/No penetrative sex(0)<br>Every time (1)<br>Often (2)<br>Sometimes (3)<br>Rarely (4)<br>Never (5)		Omitted. Minimal contribution to risk.
9. How often did you use condoms when you had sex with casual partners in the last month?<br><br>No casual partners/No penetrative sex (0)<br>Every time (1)<br>Often (2)<br>Sometimes (3)<br>Rarely (4)<br>Never (5)	4. How often did you use condoms when you had sex with casual partners# in the last month?<br><br>No/casual partner (0)<br>Every time (1)<br>Often (2)<br>Sometimes (3)<br>Rarely (4)<br>Never (5)<br><br>#Include paid sex and only penetrative sex	Retained as is with clarification

10. How often have you used condoms when you have been paid for sex in the last month?<br><br>No paid sex/No penetrative sex (0)<br>Every time (1)<br>Often (2)<br>Sometimes (3)<br>Rarely (4)<br>Never (5)		Omitted. Paid sex included under question 5 (above)
	5. Have you used PreP in the last month (pre-exposure prophylaxis?<br><br>No (0)<br>Yes (1)	

18.2 APPENDIX 2: UNSW Clinical Trial Delegation Log

Delegation of Authority Log - **Tina Trial** **Site:**

The purpose of this form is to: a) serve as the Delegation of Authority Log and b) ensure that the individuals performing study-related tasks/procedures are appropriately trained and authorized by the investigator to perform the tasks/procedures. This form should be completed prior to the initiation of any study-related tasks/procedures. The original form should be maintained at your site in the study regulatory/study binder. This form should be updated during the course of the study as needed.

***THIS FORM IS TO BE COMPLETED BY ALL PERSONNEL INVOLVED IN THE STUDY AFTER RECEIVING PROPER STUDY TRAINING AND BEFORE TAKING PART IN ANY STUDY ACTIVITIES**

UNSW Sponsor's Delegate: Before commencing a clinical trial that UNSW will be responsible for as a trial sponsor, written confirmation from the UNSW Sponsors Delegate must be obtained.

Coordinating Principal Investigator (PI)

By signing, I confirm/acknowledge that the UNSW Sponsor's Delegate confirmed UNSWs role as trial sponsor and tasks listed below will only be delegated to appropriately trained, skilled and qualified staff. I will remain responsible for the overall study conduct and reported data, ensuring study oversight. All associates, colleagues, and employees assisting in the conduct of the study are informed about their obligations and have not performed any study tasks before appropriate delegation and completion of appropriate training. Mechanisms are in place to ensure that site staff receives the appropriate information and training throughout the study and that a 2-way communication channel exists between staff and self. Any changes in staff or delegation in staff will be recorded on time.

Name	Principal Investigator's Signature	Initials	Start (dd/mmm/yyyy)	End (dd/mmm/yyyy) (complete only if prior to end of study)

Site Principal Investigator (PI)

By signing, I confirm/acknowledge that the tasks listed below will only be delegated to appropriately trained, skilled and qualified staff. I will remain responsible for the overall study conduct and reported data, ensuring study oversight. All associates, colleagues, and employees assisting in the conduct of the study are informed about their obligations and have not performed any study tasks before appropriate delegation and completion of appropriate training. Mechanisms are in place to ensure that site staff receives the appropriate information and training throughout the study and that a 2-way communication channel exists between staff and self. Any changes in staff or delegation in staff will be recorded on time.

Name	Principal Investigator's Signature	Initials	Start (dd/mmm/yyyy)	End (dd/mmm/yyyy) (complete only if prior to end of study)

	Obtain Informed Consent*	Inform participant about medical interventions*	Subject Selection/Recruitment*	Confirm Eligibility (review inclusion/ exclusion criteria)*	Conduct study visit procedure as outlined in the protocol*	Use IWRS/IVRS ((Interactive Web and voice Response Systems)	Make entries/corrections on (e)CRFs	Perform study-related assessments as per protocol*	Sign off eCRFs*	Sample collection	Make study-related medical decisions*	Perform physical exam*	Obtain Medical History (source documents)	Complete company- specific log (if applicable)	Maintain Essential Documents	Evaluate study related test results*	Sample processing and/or shipment	Assess AEs/SAEs	*Perform drug accountability	Study drug storage and temperature monitoring	Dispense study drug*	MEMS activation and data collection	Other: ___	Other: ___	PI Initials/date
ROLE: Site Lead	☐	☐	☐	☐	☐	☐	☐	☐	☐	☐	☐	☐	☐	☒	☐	☐	☐	☐	☐	☐	☐	☐	☐	☐	Initials:
NAME:		SIGNATURE:								START DATE:									END DATE: (complete only if prior to end of study)						Date:
ROLE: Site Trial Coordinator	☐	☐	☐	☐	☐	☐	☒	☐	☒	☒	☐	☐	☐	☒	☒	☐	☐	☐	☐	☐	☐	☐	☐	☐	Initials:
NAME:		SIGNATURE:								START DATE:									END DATE: (complete only if prior to end of study)						Date:
ROLE: Trial Researcher	☒	☐	☒	☐	☒	☐	☒	☒	☐	☐	☐	☐	☐	☒	☒	☐	☒	☒	☐	☐	☐	☐	☐	☐	Initials:
NAME:		SIGNATURE:								START DATE:									END DATE: (complete only if prior to end of study)						Date:
ROLE: Trial Physician	☐	☒	☐	☒	☐	☐	☐	☐	☐	☐	☒	☒	☒	☐	☒	☒	☐	☒	☐	☐	☐	☐	☐	☐	Initials:
NAME:		SIGNATURE:								START DATE:									END DATE: (complete only if prior to end of study)						Date:
ROLE: Pharmacist	☐	☐	☒	☐	☐	☐	☐	☐	☐	☐	☐	☐	☐	☐	☐	☐	☐	☐	☒	☒	☒	☒	☐	☐	Initials:
NAME:		SIGNATURE:								START DATE:									END DATE: (complete only if prior to end of study)						Date:

*These tasks may only be performed by a qualified individual as permitted by local law, medical or standard of care practices, or applicable required training as per job description or designation.

Electronic Signature Declaration for Principal Investigator and Site Staff

- As it applies to entering electronic data or signing records in sponsor-owned or sponsor-sourced computer systems, my electronic signature is the legally binding equivalent of my handwritten signature.
- I will not share the password(s) assigned to me for this study with any other persons.

Principal Investigator's End of Study Declaration

I hereby confirm that the above information is accurate and complete and that I authorised the delegation of study-related tasks to each individual as listed above.

- **Principal Investigator's Signature:** ____ **Date:** ___________________
