# Supplementary material for: Mirtazapine for Methamphetamine Use Disorder: A Randomized Clinical Trial
Source: JAMA Psychiatry. 2026 Apr 1;83(6):581–9. doi: 10.1001/jamapsychiatry.2026.0159 (PMC13044789; doi:10.1001/jamapsychiatry.2026.0159)
Supplement: Supplement 2. — Statistical Analysis Plan [file jamapsychiatry-e260159-s002.pdf]

STATISTICAL ANALYSIS PLAN FOR THE TINA TRIAL:
A RANDOMISED DOUBLE BLIND PLACEBO CONTROLLED TRIAL OF MIRTAZAPINE AS A PHARMACOTHERAPY FOR METHAMPHETAMINE DEPENDENCE

VERSION NUMBER 2.0

MAY 1, 2025

1 Contents

1 Contents 2
1. Administrative information 6
1.1 Version history 7
2 Introduction 7
2.1 Rationale 7
2.2 Objective 8
2.3 Statistical Hypotheses 8
3 Study methods 8
3.1 Trial design 8
3.2 Randomisation 8
3.3 Sample size 8
3.4 Framework 9
3.5 Statistical interim analysis and stopping guidance 9
3.6 Timing of final analysis 9
3.7 Time points at which outcomes were measured 9
3.8 Description of measures 9
4 Trial population 11
4.1 Eligibility criteria 11
4.2 Screening and recruitment 12
4.3 Reasons for screen and eligibility failures 12
4.4 Medication discontinuation 15
4.5 Withdrawal of participants from the study 15
4.6 Unblinding of participants 16
4.7 Available assessment data 16
4.8 Medication adherence 18
4.9 Sample characteristics 19
4.10 Characteristics of outcome data 19
4.11 Beliefs about condition allocation 19
5 Statistical Principles and definitions 23
5.1 Confidence intervals and p values 23
5.2 Analysis populations 23
5.3 Protocol deviations 23
5.4 Outcome definitions 23
6 Analysis 25

6.1 General approach to planned analyses ... 25
6.2 Planned analysis of primary outcome ... 26
6.3 Planned analysis of the secondary outcomes ... 27
6.4 Planned analysis of the tertiary and exploratory outcomes ... 28
6.5 Sensitivity analyses ... 28
6.6 Additional analyses ... 29
6.7 Exploratory analyses and embedded studies ... 29
6.8 Missing data ... 29
6.9 Statistical software ... 30
7 Related documents ... 30
8 Roles and responsibilities ... 30
9 Appendix ... 31
10 References ... 35

Table of tables

Table 1 Timeframes for each assessment measure and timepoints at which they were measured.......... 10

Table 2 Reasons for ineligibility at the phone screen (n = 338) and eligibility assessment (n = 57) 14

Table 3. Number of follow-up assessments started and availability of assessment data for self-reported days of methamphetamine use and oral fluid samples 17

Table 4 The number of missing data points for measures taken at baseline, week 4, week 8 and week 12[a] 18

Table 5. Sample characteristic variables and how they will be presented 20

Table 6 Description of outcome measures 22

Table of figures

Figure 1 Consort Flow Diagram 13

List of abbreviations

AE	Adverse Event
ADR	Adverse Drug Reaction
BMI	Body Mass Index
AIS-5	Athens Insomnia Scale
CSSRS-S	Columbia Suicide Severity Rating Scale - Screener
GAD-7	General Anxiety Disorder 7 Item Scale
HREC	Human Research Ethics Committee
eCRF	Electronic Case Report Form
EQ-5D	EuroQol 5D
HRBS	HIV Risk Behaviour Scale
HSU	Health Service Utilisation
ITT	Intention-To-Treat
MedDRA	Medical Dictionary for Regulatory Activities
PGI	Patient Global Impression
PHQ-9	Patient Health Questionnaire – 9
RCT	Randomised Controlled Trial
SAE	Serious Adverse Event
SOC	System Organ Class
TLFB	Timeline Followback
TSQM	Treatment Satisfaction Questionnaire for Medication Version 2
WPAI-GH	Work Productivity and Activity Impairment Questionnaire: General Health

1. Administrative information

Project title: Tina Trial: A phase 3 randomised double-blind placebo-controlled trial of mirtazapine as a pharmacotherapy for methamphetamine dependence

Protocol Version Number: Version 12.0, 28th October 2024

Registration: Australian and New Zealand Clinical Trials Registry (ACTRN12622000235707). Registered on February 9, 2022. The Universal Trial Number (UTN): U1111-1271-8220.

Overall study sponsor: University of New South Wales. This research is being conducted jointly by the NHMRC investigator team and their institutions under a co-sponsorship arrangement. Please refer to the study protocol for details.

Funding body: Medical Research Future Fund (Grant No. 2007155)

Authors:

Principal Coordinating Investigator: Rebecca McKetin, National Drug and Alcohol Research Centre, University of New South Wales

Signature: ____________________ Date: ___1 May 2025_______

Biostatistician: Philip Clare, National Drug and Alcohol Research Centre, University of New South Wales

Signature: ____________________ Date: ________________

1.1 Version history

Version	Date	Description of Change	Brief Rationale
1.0	23/4/2025	N/A	N/A
2.0	1/5/2025	Correction to scoring of the AIS-5	The scoring of the AIS-5 was incorrect and the score range cited as 0-20 instead of 0-15.

2 Introduction

2.1 Rationale

Methamphetamine (also known as "crystal meth" or "ice") is a significant and growing global public health concern with an estimated 7.4 million people worldwide dependent on the drug [1, 2] (i.e., experiencing what is now termed a 'methamphetamine use disorder' under the DSM 5). Methamphetamine use disorder is a chronic, relapsing condition [3] that is associated with elevated mortality, increased incidence of HIV and hepatitis C infection, poor mental health (suicidality, psychosis, depression, and violence), and increased risk of cardiovascular events [4]. Use accounts for a significant excess number of psychiatric hospital admissions and emergency department presentations in Australia [5]. In the USA, methamphetamine is increasingly used in conjunction with opioids, and this is contributing to the growing number of opioid-related overdose deaths [6].

Currently there are no approved pharmacotherapies to assist with the treatment of methamphetamine use disorder [7, 8]. Although individual trials have found positive results (e.g., long-acting slow-release amphetamine preparations [9] and combination naloxone and bupropion [10]), many promising options have failed to produce the expected reductions in methamphetamine use [11, 12], and there remains insufficient evidence to support any pharmacotherapy option for methamphetamine use disorder [13]. Effective pharmacotherapy has the potential to dramatically increase treatment coverage, enhance treatment engagement and retention, and mitigate against poor health outcomes for people with methamphetamine use disorder.

One promising pharmacotherapy candidate is the generic tetracyclic antidepressant, mirtazapine, with significant reductions in methamphetamine use found in two preliminary phase 2 trials in the United States [14] [15]. The first trial (N = 60) found 12 weeks of mirtazapine (30 mg/day) significantly reduced methamphetamine use relative to placebo amongst sexually active men who have sex with men [14]. The second trial replicated these positive results after 24-weeks of mirtazapine treatment with a larger sample of cisgender men, transgender men, and transgender women, who have sex with men (N = 120) (positive urine test results at 24 weeks relative to placebo 63% vs. 74%; RR 0.75, 95% CI 0.56 – 1.00, p = 0.04) [15]. In the second trial, mirtazapine also reduced depressive symptoms and improved sleep [15]. A meta-analysis of outcomes for these trials showed consistent signal for

mirtazapine for reduction methamphetamine use, though not for reducing depressive symptoms [16]. The consistent signal across these trials for reducing methamphetamine indicates a need for a phase 3 trial to demonstrate the benefits of mirtazapine when used in a broader population in routine clinical practice.

2.2 Objective

The objective of this trial (the Tina Trial) is to establish the effectiveness and safety of mirtazapine as an outpatient pharmacotherapy for methamphetamine use disorder in routine clinical practice in an Australian setting.

2.3 Statistical Hypotheses

The primary hypothesis is that oral mirtazapine (30 mg/day for 12 weeks) will, compared to placebo, reduce self-reported days of methamphetamine use.

The secondary hypotheses are that oral mirtazapine (30 mg/day for 12 weeks) will, compared to placebo:

- increase abstinence from methamphetamine use, as assessed by methamphetamine negative oral fluid samples,
- reduce depressive symptoms,
- improve sleep quality,
- reduce HIV risk behaviour, and
- improve quality of life.

3 Study methods

3.1 Trial design

The Tina Trial is a multi-site double-blind randomised placebo-controlled parallel group, two arm, superiority trial with a 1:1 allocation ratio.

3.2 Randomisation

Eligible participants are randomly assigned (1:1) to receive either placebo or mirtazapine based on a computer-generated permutated block randomisation sequence with variable block sizes, stratified by site, sex (male vs. female or other) and depression (PHQ-9 score < 10 vs. 10 or greater) assessed at eligibility.

3.3 Sample size

The estimated sample size required for the study (N = 340; 170 per group) was to enable us to detect a minimum rate ratio of 0.75 on our primary outcome (equivalent to a relative reduction from 25 days methamphetamine use in the past 4 weeks at baseline to 20 days use in the past 4 weeks at week 12) with 90% power (two-tailed test, p = 0.05). This sample size calculation was based on the effect size found in the most recent trial of mirtazapine for methamphetamine dependence [15] and allows for up to 25% attrition (ie., a final sample of at least 255, or 128 per group, at week 12).

We randomised 344 participants in the trial, of whom 339 participants received the trial medication and 293 completed their week 12 assessment, meeting the sample size requirements estimated above.

3.4 Framework

Superiority of active medication (mirtazapine) over placebo.

3.5 Statistical interim analysis and stopping guidance

No interim analysis or stopping guidelines were included in the protocol.

3.6 Timing of final analysis

Final analysis will take place after locking the electronic Case Report Form (eCRF) database, which includes all outcome assessments and safety (adverse event) data. This will take place after the final week 20 assessment has been conducted or after the last assessment date that this could have taken place (May 15, 2025).

Analysis of primary and secondary endpoints to be analysed collectively. Sensitivity analyses, sub-group analyses, analysis of tertiary endpoints, and any additional/exploratory analyses, to take place thereafter.

3.7 Time points at which outcomes were measured

The time points at which each of the outcomes were assessed and the measurement timeframe for each outcome assessment is shown in Table 1.

3.8 Description of measures

A detailed description of measures taken in the study is provided in the Appendix. The specific outcomes used in the analysis are detailed in Section 6.

Table 1 Timeframes for each assessment measure and timepoints at which they were measured

	Pre-intervention		Intervention (30 mg/day)					Taper dose	Final medical	Post-study assessment
**Assessment**	Eligibility assessment[a]	Eligi-bility medical	Wk 0 (Baseline)	Wk 2	Wk 4	Wk 8	Wk 12	Wk 16	Wk 18	Wk 20
Demographics and drug use history	Current/ Lifetime									
Methamphetamine use disorder (CIDI)	Past year									
Abstinence from methamphetamine (oral fluid samples)					Current	Current	Current			
Days of methamphetamine use (TLFB)	Past 4 wks		Past 4 weeks		Since last Ax[b]	Since last Ax[b]	Since last Ax[b]			Past 4 weeks
Days of other substance use	Past 4 wks		Past 4 wks		Past 4 wks	Past 4 weeks	Past 4 weeks			Past 4 weeks
Depression (PHQ-9)	Past 2 wks		Past 2 wks		Past 2 wks	Past 2 wks	Past 2 wks			Past 2 wks
Suicidality screener (CSSRS-S)	Past month[c]		Since last Ax		Since last Ax	Since last Ax	Since last Ax			Since last Ax
Anxiety (GAD – 7)			Past 2 wks		Past 2 wks	Past 2 wks	Past 2 wks			
Sleep (AIS-5)			Past month		Past month	Past month	Past month			
HIV Risk (HRBS)			Past month		Past month	Past month	Past month			
Quality of life (EQ-5D)			Today		Today	Today	Today			
Work productivity (WPA-GH)			Past wk				Past wk			
Patient centred assessment (PGI)					Since Wk 0	Since Wk 0	Since Wk 0			
Tolerability/satisfaction (TSQM II)							Past 2-3 wks			Since last used
Other questions on medication tolerability				Current[d]	Current[e]	Current[e]	Current	Current[d]		
Health service use			Past 3 months				Since Wk 0			Since last Ax
Body Mass Index – self-report	Current						Current			
Body Mass Index – verified		Current							Current	
Concomitant medications	Past 2 wks		Since last Ax		Since last Ax	Since last Ax	Since last Ax	Since last Ax		Since last Ax
Adverse events				Since Wk 0	Since last Ax	Since last Ax	Since last Ax	Since last Ax	[f]	Since last Ax
Medication adherence (MEMS®)					Since Wk 0	Since last Ax	Since last Ax			

Notes. [a]Done within 28 days of Wk 0 [b]Up to 35 days prior to assessment [c]Timeframe could vary [d]Only one specific questions about known medication side-effects [e]Unpublished questions on medication tolerability [f]Adverse events are reviewed by the Trial Physician and information on adverse events may be updated at this timepoint. Abbreviations: Week (Wk), Assessment (Ax), Timeline Followback (TLFB), Patient Health Questionnaire–9 (PHQ-9), Athens Insomnia Scale (AIS), HIV Risk Behaviour Scale (HRBS), Euroqol 5D (EQ-5D), Columbia Suicide Severity Risk Scale Screener (CSSRSS), Patient Global Impression (PGI), Composite International Diagnostic Interview (CIDI) modified to capture a DSM 5 methamphetamine use disorder, the Generalised Anxiety Disorder 7 item scale (GAD-7), Work Productivity and Activity Impairment Questionnaire – General Health V2 (WPAI-GH) Treatment Satisfaction Questionnaire for Medication Version II (TSQM II).

4 Trial population

4.1 Eligibility criteria

Inclusion criteria

1. Aged between 18 and 65 years.
2. Moderate to severe methamphetamine use disorder in the past year (DSM-5 past year diagnosis confirmed at Eligibility Assessment using modified version of the Composite International Diagnostic Interview).
3. Current methamphetamine use (defined as using at least twice weekly in past 4 weeks based on the participant's self-reported use, and a positive drug screening test for amphetamines).
4. Willing to use effective contraception (for women only).
5. Willing to provide contact details for a treating physician.
6. Willing to provide contact details for follow-up.
7. Able to provide informed consent and able to comply with the study protocol.

Exclusion criteria

1. In need of acute care (e.g., suicidality or acute psychosis, unstable psychiatric condition; medical detoxification).
2. Pregnant or lactating.
3. Incarceration or current inpatient treatment (including residential rehabilitation, inpatient detoxification)*.
4. Currently taking prescribed antidepressant medication.
5. Any use of monoamine oxidase inhibitors in the 14 days prior to starting the trial medication.
6. Contraindications for mirtazapine, including:
    a. a known hypersensitivity to mirtazapine
    b. use of antidepressant medication (including monoamine oxidase inhibitors, St. John's Wort, or SSRIs) or other serotonergic drugs, and
    c. galactose intolerance, Lapp lactase deficiency or glucose-galactose malabsorption (lactose is an excipient in the trial medication).
7. High risk of adverse reactions to mirtazapine including suicide, overdose, sudden cardiac death, risk of agranulocytosis, or accidents and injuries from motor impairment.
8. Past year suicide attempt.
9. Unable or unwilling to avoid pregnancy during the trial (for both men and women).
10. Participation in another clinical trial.

*This applies to the status of the participant at trial enrolment and does not preclude the participant from entering treatment or receiving other care during the trial.

4.2 Screening and recruitment

A total of 1,267 participants were phone-screened for the trial (Figure 1). Of these prospective participants:

- 338 were not eligible based on the initial phone screening
- 29 did not complete the initial phone screening
- 900 were potentially eligible on the phone screen:
    - 453 of these participants did not initiate a face-to-face eligibility assessment: 4 presented at the eligibility assessment but declined to consent; 1 presented to the eligibility assessment but was not consented to the study because they required acute health care; and, the remaining 448 were lost to follow-up (i.e., participants did not show for their eligibility assessment, wait-listed participants who could not be recontacted or were no longer interested in participating).
    - 447 were consented to the study and started a face-to-face eligibility assessment: 368 were eligible, 58 were not eligible, and 21 did not complete the eligibility assessment (5 declined to continue with participation, 2 were in need of acute care, 1 assessment was terminated because the participant had no recent methamphetamine use, and 13 failed to show for their eligibility medical assessment).

Of the 368 eligible participants, 344 were randomised and 24 were not randomised (were lost to follow-up after eligibility or declined to participate).

Of the 344 randomised participants, 5 failed to attend their baseline assessment and hence did not receive the trial medication. Of the remaining 339 participants who received their trial medication, 20 participants did not attend any further assessments after baseline. Details of follow-up for the remaining participants can be found in Figure 1.

4.3 Reasons for screen and eligibility failures

Reasons for screen failure at the phone screening and face-to-face eligibility assessment are shown in Table 2. The most common reasons for not being eligible were that participants were currently taking prescribed antidepressants, they did not meet the methamphetamine use criteria, or they had other factors that meant they would be unable to comply with the protocol (e.g., pending incarceration, living outside of the area health service, unable to understand the protocol and/or provide informed consent, or were in need of acute care).

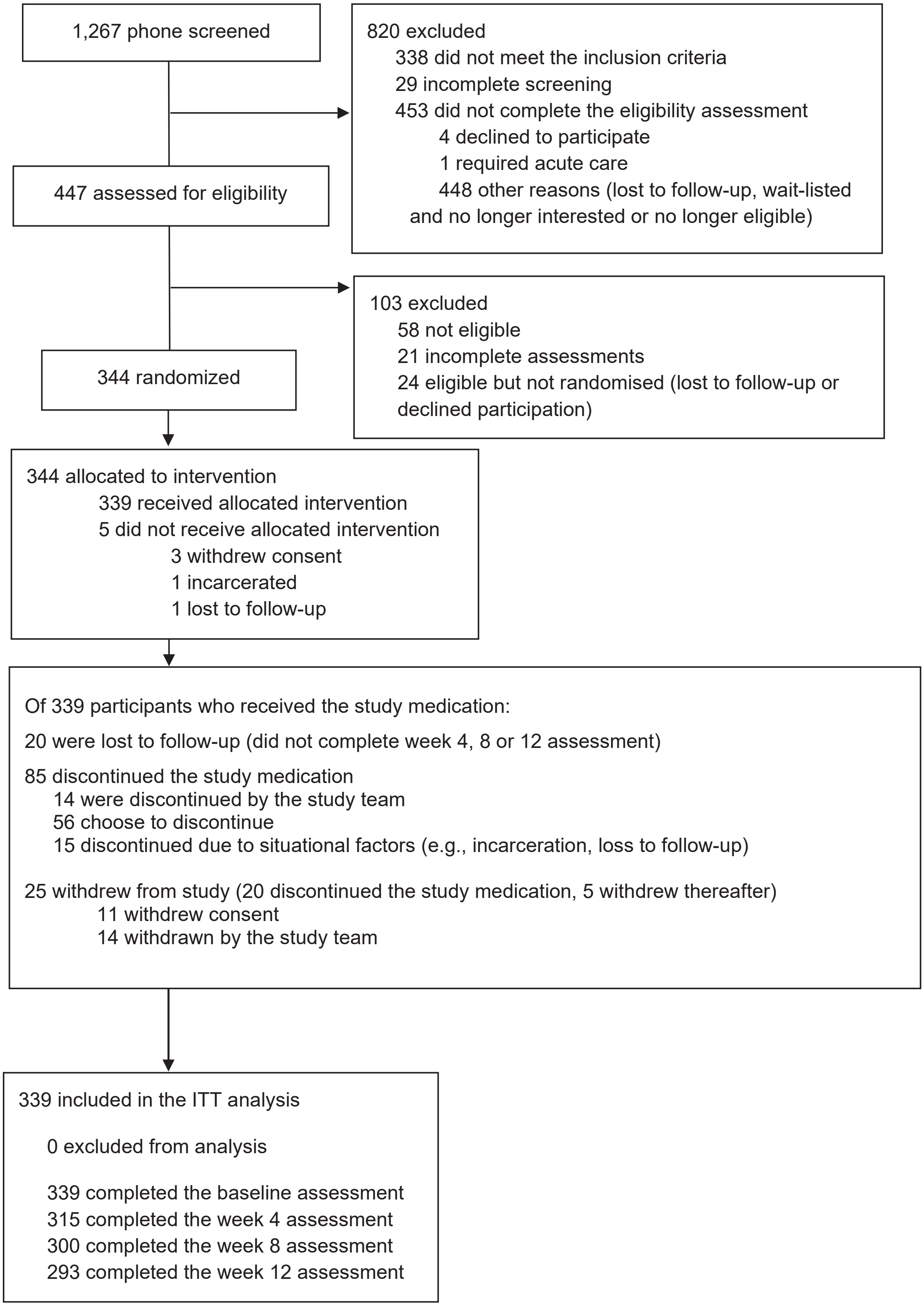

Figure 1 Consort Flow Diagram

Table 2 Reasons for ineligibility at the phone screen (n = 338) and eligibility assessment (n = 57)

**Reason:**	Phone screen		Eligibility assessment		Total	
	N =338	%	N = 57	%	N = 395	%
Outside area	60[a]	18	0	0	60	15
**Inclusion criteria**						
18 and 65 years	1	<1 %	0	0	1	< 1
Moderate to severe methamphetamine use disorder	N/A		5	9	5	2
Current methamphetamine use (using twice weekly + positive drug screen for amphetamines at the eligibility assessment)	120[b]		19[b,c]	33	139	10
Willing to use effective contraception (women only)	N/A		0	0	0	0
Contact details for treating physician	N/A		5	8	5	1
Contact information for follow-up	2	4	0	0	2	< 1
Able to provide informed consent and comply with protocol	0	0	28[d]	49	28	7
**Exclusion criteria**						
In need of acute care (e.g., suicidality or acute psychosis, unstable psychiatric condition; medical detoxification)	N/A		11	19	11	3
Pregnant or lactating	1	<1	1	2	2	<1
Incarceration or current inpatient treatment	9	3	0	0	9	2
Currently taking prescribed antidepressant medication	115	34	4	7	119	30
Monoamine oxidase inhibitors in the 14 days	0	0	0	0	0	0
Contraindications for mirtazapine	5[e]	1	2	4	7	2
High risk of adverse reactions to mirtazapine	N/A		19	33	19	5
Past year suicide attempt	27	8	5	9	32	8
Unable or unwilling to avoid pregnancy during the trial	5	1	1	2	6	2
Participation in another clinical trial	1	<1	0	0	1	<1

Notes. Phone screens terminate at the first ineligible criterion. At the face-to-face eligibility assessment, participants could be ineligible on more than one criterion. For this reason, the sum of the number of participants ineligible on each criterion is greater than the number of ineligible participants.
[a]This does not include participants who did not initiate screening because they did not live near any of the trial sites. [b]Self-reported use of methamphetamine was less than twice weekly [c]Negative drug screen for amphetamines [d]This included other circumstances that would preclude participation such as an inability to understand the consent form, an inability to comply with the study protocol (e.g., unable to attend assessments), or impending incarceration. [e]Self-reported lactose intolerance

4.4 Medication discontinuation

Of the 339 participants who attended their baseline assessment and received the trial medication, 85 participants were permanently discontinued from the 30 mg trial medication. (Note. Participants who did not receive or stopped taking the 15 mg taper dose were not recorded as medications discontinuations under the study protocol.)

- 14 participants were discontinued from the medication by the study team (e.g., due to adverse reactions or other medical risks).
- 56 participants chose to stop the medication (e.g., because they did not like the side-effects or no longer wanted to participate in the study).
- 15 participants ceased medication due to circumstantial factors (e.g., incarceration, loss to follow-up, being withdrawn from the study).

Participants who discontinued the trial medication included 20 participants who were withdrawn from the study prior to week 12 (Figure 1). The remaining 65 participants who were discontinued from the study medication were followed up for study assessments.

4.5 Withdrawal of participants from the study

Of the 339 participants who attended the baseline assessment and were provided with their medication, 25 were subsequently withdrawn during the study, meaning they had no follow-up data after the date of withdrawal. Twenty of these 25 participants were withdrawn during the intervention phase of the study (i.e., between baseline and the week 12 assessment, whilst receiving the 30 mg study medication). These participants are also recorded as having discontinued the trial medication.

Reasons for withdrawal are provided below.

- 11 participants withdrew their consent because they no longer wanted to participate in the study.
    - Five participants cited situational factors, such as not having the time to attend study appointments due to work or other time commitments.
    - Three participants felt that the trial medication or the trial in general was not helping them, and they did not wish to continue.
    - One participant had a decline in their mental health and wanted to seek other treatment rather than participate in the trial.
    - Two participants did not disclose why they withdrew.

- 14 were withdrawn by the study investigators.
    - Six were incarcerated.
    - Five were unable to comply with the protocol, failing to attend appointments or respond to communication (i.e., lost to follow-up).
    - Two participants were unable to comply with the protocol because they breached the health service regulations.
    - One required acute mental health care.

-

4.6 Unblinding of participants

Three participants were unblinded during the study:

- Two participants were unblinded in the context of pregnancy (study IDs 221001 and 222007). Both were on placebo.
- One participant (722003) was accidentally unblinded due to an error by the trial pharmacist. The site trial researcher doing the assessments became aware of the participant's condition allocation. Subsequent assessments for this participant were conducted by a different trial researcher who was blind to the participant's condition allocation. Neither the participant nor the other study team members became aware of the participant's condition allocation.

4.7 Available assessment data

Assessments attended

The number (%) of participants starting each assessment is shown in Table 3.

Five participants failed to complete their baseline assessment, and therefore did not receive the trial medication. These participants were not included in the intention-to-treat dataset.

Of the participants who completed their baseline assessment and were provided with the trial medication (N = 339), 315 (93%) started the week 4 assessment, 300 (89%) started the week 8 assessment, and 292 (86%) started the week 12 assessment. The overall follow-up of participants across the week 4, 8 and 12 assessments was 89% (907 of 1,014 assessments were started).

Week 2 adverse event assessments were completed on 320 of the 339 participants (94%) who received the trial medication.

Final data on the week 16 adverse event assessment, the final medical assessment, and the week 20 assessment were not available at the time of writing this statistical analysis plan.

Available outcomes data

There were several cases where assessments were started but data collection was not completed (e.g., because the interview was terminated or questionnaire items were skipped) or where data could not be collected because the assessment was done by phone (i.e., biological samples, weight) (Table 4).

Primary outcome of days of methamphetamine use

Note data on the TLFB is censored if a participant is incarcerated or is in an inpatient facility. This can reduce the number of days observed in the TLFB assessment window. The observed number of days can also vary depending on the actual assessment date.

Data on days of methamphetamine use at week 12 was available for 86% of participants (n = 293) (Table 4). Across weeks 4, 8 and 12 data on days of methamphetamine use was available for 89% of these assessment periods. Details for data availability at other assessments can be found in Table 4.

There was no missing data for the TLFB data for started assessments. However, data for 2 participants at weeks 8 and 12 (i.e., 4 data points) will be censored from the analysis of the TLFB data because these participants were in an inpatient facility for the entire assessment window (i.e., they had zero days of observation on their TLFB).

The median (interquartile range) days of methamphetamine use in the four weeks prior to the baseline (Week 0) assessment was 24 days (17-24 days; range 1 – 28 days). This was over a median of 28 days (IQR 28 to 28 days; range 16 to 28 days).

The median (interquartile range) days of methamphetamine use during week 12 assessment period was 17 days (9-24 days; range 0 – 34 days). This was over a period with a median of 28 days (IQR 26 to 28 days; range 0 to 34 days).

Across the three follow-up assessment periods (weeks 4, 8 and 12), the median days of methamphetamine use was 18 days (IQR 10-25; range 0-34) and the median number of observed days for these periods was 28 days (IQR 27-28 days; range 0-34).

Secondary outcome of methamphetamine abstinence (oral fluid tests)

Oral fluid test samples were taken at 96% of assessments conducted at week 4, 8 and 12 assessments (or 86% of all possible assessments), giving 871 oral fluid samples across the week 4, 8 and 12 assessments (Table 4). The analysis of oral fluid samples was yet to be completed at the time of writing this statistical analysis plan.

Missing assessment data for planned analyses

Table 4 documents how many missing data points there were for assessments that were started. These missing data points were due to incomplete interviews or missed survey items. Oral fluid tests are missed when assessments were conducted by phone and it was not possible to follow-up with the participant in person to collect the oral fluid sample.

At the time of writing this statistical analysis plan, toxicology results were available for 797 oral fluid samples, 73 samples were pending analysis, and one oral fluid sample was not able to be analysed because of insufficient oral fluid (week 4 assessment in Brisbane).

Tertiary/exploratory outcomes that are not included here include concomitant medications, adverse events, treatment satisfaction, health economic measures (health service use and the WPAIQ-GH) and Body Mass Index.

Table 3. Number of follow-up assessments started and availability of assessment data for self-reported days of methamphetamine use and oral fluid samples

	Week 4	Week 8	Week 12	Total
Number of assessments due	339	339	339	1,017
Number (%) of assessments conducted	315 (93%)	300 (89%)	293 (86%)	907 (89%)
Number (%) with primary outcome (TLFB)	315 (93%)	300 (89%)	293 (86%)	908 (89%)
Number (%) with oral fluid samples	305 (90%)	287 (85%)	279 (82%)	871 (86%)

Table 4 The number of missing data points for measures taken at baseline, week 4, week 8 and week 12[a]

	Baseline N = 339	Week 4 N = 315	Week 8 N = 300	Week 12 N = 293
Assessment started	339	315	300	293
Primary outcome				
Days of methamphetamine use (TLFB)	0	0	0[b]	0[b]
Secondary outcomes				
Abstinence (oral fluid tests)	N/A	10	12	14
Depression (PHQ-9)	3	2	3	4
Sleep (AIS-5)	0	1	3	2
HIV risk (HRBS)	0	0	2	5
Quality of life (EQ-5D)	1	1	4	5
Tertiary outcomes				
Other drug use	0	0	0	1
Anxiety (GAD-7)	2	1	2	2
Suicidality (CSSRS-S)	N/A	0	0	3[c]
Patient satisfaction (PGI)	N/A	0	1	0

[a]Includes only assessments that were started

[b]Data for 2 participants at week 8 and week 12 (4 data points) were censored due to in-patient care covering the entire assessment window

[c]Three participants with missing data at week 12 did not have any suicidal ideation reported at weeks 4 and 8. They have been left as missing data points at week 12.

4.8 Medication adherence

Adherence to the trial medication was monitored using a medication event monitoring system, MEMS® Smart Caps, which recorded the date and time of each bottle opening. Each of the 30 mg medication bottles provided to the participant was fitted with a MEMS Smart Cap. A new 30 mg medication bottle was provided to each participant at baseline, week 4 and week 8. Adherence data from all returned bottles was collated in the MEMS software, providing continuous adherence data for the 12-week intervention period.

Adherence data was recorded as the percentage of adherent medication days during the assessment window (i.e., since the last assessment date at weeks 4, 8 and 12). An adherent medication day was defined as any bottle opening in a 24-hour period (ending at 3 am) with no penalty for multiple openings.

Adherence data was recorded as missing if the medication bottle for that assessment window was dispensed and not returned. Adherence data was excluded from the analysis (recorded as missing) if the medication bottle for that assessment window was dispensed and returned but a previously dispensed medication bottle was not returned. This was done because participants could (and often would) continue to take medication from the previously dispensed but unreturned bottle, rendering the adherence data for subsequent bottles incomplete.

The calculation of adherence for the 12-week trial medication period will be based on an average of the medication adherence for each assessment window, adjusting for the number of observed days in each assessment windows.

<u>Available adherence data:</u> 900 bottles with MEMS Smartcaps were dispensed (339 at baseline, 296 at week 4 and 265 at week 12). Medication bottles could be returned at any until the close of the study. At the time of writing this statistical analysis plan, 296 of the bottles dispensed at baseline had been returned, 258 of the bottles dispensed at week 4 had been returned, and 229 of the bottles dispensed at week 8 had been returned (783 bottles in total, 87% of dispensed bottles).

4.9 Sample characteristics

Sample characteristics will be based on demographics and drug use data collected at the eligibility assessment and will only include those participants in the intention-to-treat analysis dataset (i.e., participants who attended their baseline assessment and received the trial medication).

Summary variables are presented in Table 5 along with how they will be reported and their summary values.

4.10 Characteristics of outcome data

Table 6 documents how outcomes data will be presented. It also includes baseline and follow-up summary values for the data available at the time of writing this report. Oral fluid test results were not available at the time of writing this report. Tertiary outcomes are included where analyses have been planned. Tertiary outcomes not included here include concomitant medications, adverse events, treatment satisfaction, health economic measures (health service use and the WPAIQ-GH) and Body Mass Index.

4.11 Beliefs about condition allocation

At weeks 4 and 8 participants were asked whether they thought they were taking the active medication (mirtazapine) or the placebo pill, and given the response options of 'Don't know', 'Placebo' and 'Active'. At week 4, 41% thought they were on the active medication, 29% thought they were on placebo, 30% said they didn't know. At week 8, 39% thought they were on the active medication, 31% thought they were on placebo, 30% said they didn't know.

Table 5. Sample characteristic variables and how they will be presented

Baseline measure	Description	Variable	Presentation	Value
Demographics				
Age	Age in years	age	Mean (SD)	42.0 (8.6)
Sex[a]	Male (vs. female or other)	sex	n, (%)	213 (63)
Immigrant	Born outside of Australia (vs. born in Australia)	cob	n, (%)	55 (16%)
Married	Married/de-facto (vs. single, separated, divorced, widowed)	married	n, (%)	69 (20)
Employment	Current employment status	employcat	n, (%)	
	Unemployed	employcat_1		179 (63)
	Full-time employment	employcat_2		78 (23)
	Other (Casual or part-time employment, home duties, student)	employcat_3		82 (24)
Income	Net income (from all sources) in the past fortnight	incomecat	n, (%)	
	< $800,	incomecat_1		121 (36)
	$800-1119	incomecat_2		95 (28)
	> $1200	incomecat_3		123 (36)
Schooling	Years of completed school (primary and secondary)	dg_education	Median (IQR)	10 (10-12)
Qualifications	Completed tertiary qualifications (Nil, vs. Trade/technical or University)	quals		231 (68)
	Nil	quals_1	n, (%)	108 (32)
	Trade/technical	quals_2	n, (%)	206 (61)
	University	quals_3	n, (%)	25 (7)
Prison history	Ever been to prison (i.e., served a prison sentence)		n, (%)	189 (42)
Children	Number of biological children	children		
	No children	children_1	n (%)	171 (50)
	One child	children_2	n (%)	90 (27)
	Two children	children_3	n (%)	45 (13)
	Three or more children	children_4	n (%)	33 (10)
Living arrangement	Who they were living with in the past month (more than one category could apply)			
	Living alone	live_alone	n (%)	95 (28)
	Living with children (any children under the age of 16 years)	live_kids	n (%)	100 (30)
	Living with partner	live_partner	n (%)	80 (24)
	Living with other family	live_othfamily	n (%)	72 (21)
	Living with unrelated adults	live_share	n (%)	69 (20)
Accommodation	Usual accommodation in the past month	housing		
	Privately rented or owned	housing_1	n (%)	243 (72)
	Public housing	housing_2		63 (19)
	Other (boarding hous/ shelter, no fixed address, or other e.g., couch surfing, caravan)	housing_3		33 (10)
Body Mass Index	Verified weight and height (kgs/m^2)	bmi	Mean (SD)	27.0 (5.8)
Methamphetamine use				
Treatment history				
	Drug treatment history[b] (ever started drug treatment for	methtxhistory	n, (%)	154 (45)

	methamphetamine use, e.g., detox, rehab, drug counselling)			
	Received other professional help (ever received other professional help, e.g., other counselling/ psychologist, GP, narcotics anonymous etc)	dh_help	n (%)	130 (38)
	Any treatment or professional help (either of the above categories)	methhelphistory	n (%)	189 (56)
Duration of use	Years since first use of methamphetamine	methduration	Mean (SD)	20.8 (9.0)
Injecting	Main way participant took methamphetamine in the past month (inject vs. smoke, other [snort or swallow])	methinject	n, (%)	155 (46%)
Days of use	TLFB days of methamphetamine use in the past four weeks[c]	methdays	Median (IQR)	24 (18-28)
Other substance use	Days of other substance use (summed across all drug types) in the past 4 weeks	polydrug	Mean (IQR)	35.2 (19.4)

Notes. Data values provided are indicative only and may change due to data cleaning. Inter-quartile range (IQR), standard deviation (SD), Timeline Followback (TLFB).

[a]All participants identified as either male or female at birth. Current gender identity was collected from 321 of the 339 participants in the ITT sample and this matched their sex at birth.

[b]Includes detoxification rehabilitation and drug counselling.

[c]Days of incarceration or inpatient stays will not be censored from the TLFB calculation of days of methamphetamine use at the eligibility assessment. This is to ensure that all participants have the same denominator of 28 days.

Table 6 Description of outcome measures

Outcome measure	Description	Variable name	Presentation	Value			
**Primary outcome:**				Baseline	Week 4	Week 8	Week 12
Days of methamphetamine use	TLFB days of methamphetamine use in the past four weeks (or since the last assessment date up to 35 days), with an offset for the days observed.	methdays	Median (IQR) days	24 (17 - 28)	20 (12 - 26)	18 (11 - 25)	17 (9 - 24)
	Days in observed period (offset used in analysis model)	methden	Median (IQR) days	28 (28 - 28)	28 (28 - 29)	28 (27 - 28)	28 (26 - 28)
**Secondary outcomes:**							
Abstinence	Number of methamphetamine negative oral fluid samples (< 25 ng/mL)	meth_positive	n (%)	N/A	31 (11%)[a]	33 (13%)[a]	36 (14%)[a]
Depression (PHQ-9)	PHQ-9 score		Median (IQR)	9 (4 - 13)	6 (3 - 10)	5 (2-9)	6 (2 - 9)
Sleep (AIS-5)	AIS-5 score		Median (IQR)	3 (0 -6)	1 (0 - 3)	1 (0-3)	1 (0 - 4)
HIV risk	HRBS score		Median (IQR)	4 (2 - 8)	3 (1 - 6)	3 (1 - 6)	2 (1 - 6)
Quality of life (EQ-5D)	EQ-5D utility score		Median (IQR)	0.92 (0.85 - 0.97)	0.93 (0.85 - 1.00)	0.93 (0.85 - 1.00)	0.93 (0.89 - 1.00)
**Tertiary/exploratory outcomes:**							
Other substance use	Total days use for other drug classes (tobacco, alcohol, cannabis, cocaine, ecstasy, hallucinogens, inhalants, and heroin) in the past 28 days		Mean (SD)	34 (20)	31 (28 - 49)	31 (28 - 46)	30 (28 - 49)
Suicidality	Score of 3 or more on the CSSRS-S since the previous assessment		n (%)	0 (0)	N/A	N/A	11 (4)
Anxiety	Total score on the GAD-7		Median (IQR)	7 (3 - 12)	6 (2 - 11)	5 (2 - 10)	5 (1 - 10)
Patient Impression	Patient Global Impression (PGI) scale score (range 1-7)[b]		Mean (SD)	N/A	3.5 (0.9)	3.6 (0.9)	3.5 (1.2)

Notes. Data values are indicative only and may change with data cleaning. Inter-quartile range (IQR), standard deviation (SD), Timeline Followback (TLFB), Severity of Dependence Scale (SDS), Craving Experience Questionnaire (CEQ), Brief Psychiatric Rating Scale (BPRS)

[a]Based on data available at the time of writing this statistical analysis plan (797 oral fluid samples).

[b]A score of 4 represents no change. Scores lower than 4 represent a perceived improvement in health. Scores higher than 4 represent a perceived worsening of health.

5 Statistical Principles and definitions

5.1 Confidence intervals and p values

All tests will be two-tailed. Significance will be $p < 0.05$ for all outcomes. Confidence intervals will be 95%.

5.2 Analysis populations

The intention-to-treat (ITT) dataset will include all randomised participants who received the trial medication (regardless of medication adherence or follow-up).

The modified intention-to-treat analysis dataset will include randomised participants who received the trial medication and who also completed at least one follow-up assessment at weeks 4, 8 or 12.

The safety analysis dataset will include randomised participants who received the trial medication and who completed at least one follow-up assessment at weeks 4, 8 or 12 (i.e., had some safety data).

The Per-Protocol Analysis Dataset will include randomised participants who received the intervention and for whom data was available for at least one follow-up assessment at weeks 4, 8 or 12. It will censor data for participants from the point from which they permanently discontinued medication if this was for reasons unrelated to the study medication.

The Treatment Complier Analysis Dataset will include participants in the Per-Protocol Analysis Dataset that censors assessment windows in the study if the medication adherence was below the median medication adherence for the study. The median medication adherence will be based on the ITT dataset for the total baseline to week 12 assessment period, excluding assessment windows when participants were discontinued from the medication.

5.3 Protocol deviations

Protocol deviations were defined as noncompliance with the clinical trial protocol, the approved Human Research Ethics Committee protocol, or the guidelines for Good Clinical Practice in Australia. Protocol Deviations are reported to the Data Safety and Monitoring Board and Human Research Ethics Committee. The number of trial participants withdrawn from the study, discontinued from the study medication, and unblinded, will be reported. Serious deviations from the protocol will be reported where they potentially impact on the integrity of the trial data, and, if necessary, sensitivity analysis will be performed to assess their impacts.

5.4 Outcome definitions

Primary outcome

Reported days of methamphetamine use: The primary outcome is change in self-reported days of methamphetamine use in the past 4 weeks from baseline to weeks 4,8,12 and 20, assessed using the Timeline Followback (TLFB)[17], with week 12 being the primary endpoint.

Days when a participant is incarcerated or in hospital (inpatient only) are censored from the TLFB. The length of each assessment window can vary dependent on the actual follow-up date. For this reason an offset term will be included in the model to adjust for variation in the days observed in each assessment window.

TLFB data will be validated against biologically verified abstinence from methamphetamine use contained in oral fluid samples. Data on methamphetamine use from oral fluid samples was not complete at the time of writing this statistical analysis plan.

Secondary outcomes

<u>Abstinence from methamphetamine use:</u> Methamphetamine-negative oral fluid samples (< 25 ng/mL methamphetamine) taken at weeks 4, 8 and 12 using a commercial oral fluid collection device. Oral fluid is a sensitive and stable medium for the detection of methamphetamine and correlates highly with plasma tests [18].

<u>Depressive symptoms:</u> Change in the total score on the Patient Health Questionnaire-9 (PHQ-9) [19] from baseline to weeks 4, 8, 12 and 20, with week 12 being the primary endpoint. The PHQ-9 is a brief 9-item questionnaire with scores ranging from zero to 27 (higher scores indicate more depression) [19].

<u>Sleep quality:</u> Change in total score on the Athens Insomnia Scale (AIS) -5 [20] from baseline to weeks 4, 8, and 12, with week 12 being the primary endpoint. The AIS is a 5-item self-report measure that gives scores from 0 to 15 with higher scores indicating more insomnia [20].

<u>HIV risk behaviour:</u> Change in the total score on a modified version of the HIV Risk-taking Behaviour Scale (HRBS) from the Opioid Treatment Index [21] from baseline to weeks 4, 8 and 12, with week 12 being the primary endpoint. The HRBS is a validated and reliable scale that provides a composite risk index for injecting and sexual behaviour. The modified version of this scale is available from the authors on request. The scores range from 0 to 22 with higher scores indicating more risk.

<u>Quality of life:</u> Change in the utility score on the EuroQol-5D-5L (EQ-5D) [22] from baseline to weeks 4, 8 and 12, with week 12 being the primary endpoint. Utility scores in this sample range from -0.138 to 1, with higher scores indicating better quality of life.

Tertiary/exploratory outcomes included in planned analysis

<u>Suicidality</u>**:** A score of 3 or greater on the Columbia Suicide Severity Rating Scale Screener (CSSRS-S) [23, 24] at any time in the 12-week trial medication phase (i.e., wk 0 to wk 12).

<u>Other substance use:</u> Change in total days of use for other drug classes (tobacco, alcohol, cannabis, cocaine, ecstasy, hallucinogens, inhalants, and heroin) in the past 4 weeks from baseline to weeks 4, 8, 12 and 20, with week 12 being the primary endpoint. This gives a potential 224 days of other substance use (the range in this sample is 0 to 91).

<u>Anxiety</u>: Change in total scores on the Generalized Anxiety Disorder – 7 Item (GAD-7) [25] from baseline to weeks 4, 8 and 12, with week 12 being the primary endpoint. The GAD-7 is a validated screening tool for Generalized Anxiety Disorder that can also be used to assess the severity of anxiety [25]. Scores range from 0 to 21 with higher scores indicating more anxiety.

<u>Participants impression on health status:</u> The score on the single-item Patient Global Impression – Improvement (PGI - I) [26] at weeks 4, 8 and 12. The PGI provides a single score that reflects whether participants' perceive that their health status has improved since the start of the trial.

Outcomes for which there are no planned analyses

Treatment satisfaction: Scores on the Treatment Satisfaction Questionnaire for Medication Version II (TSQM II) [27] at week 12 are being used to assess treatment satisfaction. The TSQM II provides a global satisfaction summary score and subscale scores for medication convenience, side-effects, and effectiveness. Additional unpublished questions have been included at other weeks to assess tolerability and expected reactions to mirtazapine (see Appendix for details).

Concomitant medications: All medications taken by participants during the trial are recorded on a template adapted from the National Institute of Health Concomitant Medications Form [28]. For each medication is coded against the WHO generic medication code and information is recorded on the dose, frequency and route.

Health Economics: Health economics data collected include: the EuroQol 5D – 5L [22] at baseline and weeks 4, 8 and 12; the Work Productivity and Activity Impairment Questionnaire – General Health (WPAIQ-GH) V2 [29] at week 12; and, contact with health services and the criminal justice system in the three months prior to baseline and from baseline to the week 12 assessment (collected at the week 12 assessment).

Adverse events (AEs)**:** The percentage of participants reporting AEs, and serious adverse events (SAEs), by System Organ Classification, will be coded according to the Medical Dictionary for Regulatory Activities (MedDRA)[30]. AEs will be counted once only for a given participant.

Body Mass Index (BMI): BMI based on verified weight and height at eligibility and the final medical assessment.

6 Analysis

6.1 General approach to planned analyses

The analysis plan below is based on blinded data that did not include the treatment allocation variable. After unblinding, model assumptions will be checked (e.g., distribution of the outcome, cell sizes and model convergence and model fit) and models will be modified as necessary; any covariates that need to be included in the model will be identified.

Descriptive statistics will be presented as the mean (standard deviation) for continuous parametric measures and median (inter-quartile range) for highly skewed measures. Categorical variables will be presented as a percentage per category. Baseline descriptive statistics will be compared between the treatment conditions using appropriate inferential statistics. Bootstrapped confidence intervals will be used for outcomes that are highly skewed.

As a general principle, linear models will be used for normally distributed continuous outcomes. A logistic link will be used for categorical outcomes. A Poisson or negative binomial link will be used for count data and may be used for categorical outcomes with small cell sizes where this produces superior model fit. If model assumptions are breached, other model options will be examined to identify the best model fit.

A random intercept term for participant identifier will be included in all models with time-varying data model to account for clustering of data on repeated assessments. A random intercept for site will be included in models only if this significantly improves model fit (based on a likelihood ratio test; $p < 0.05$).

6.2 Planned analysis of primary outcome

The analysis of the primary outcome will be based on the intention-to-treat dataset and based on unimputed data. All tests will be two-sided with $p < 0.05$.

Descriptive data on days of methamphetamine use will include the median number of days at each time point (baseline, week 4, week 8, week 12 and week 20), with interquartile ranges reported. Variance estimates (i.e., standard errors and confidence limits) will be bootstrapped.

The main effect of medication on days of methamphetamine use will be tested using a mixed model with a group (placebo [0] vs. mirtazapine [1]) by time (baseline [0], week 4 [1], week 8 [2], week 12 [3]) interaction effect, with time entered as a factor variable, producing individual effect estimates for each time point and making no assumptions about the linearity of changes over time. The primary timepoint is week 12. A negative binomial generalised linear mixed model will be used to test this effect. As noted under the general principles above, a random intercept term will be included in the model to account for clustering on repeats [31], and a random intercept term will be included in the model to account for site only if this significantly improves model fit (based on a likelihood ratio test; $p < 0.05$).

Days of methamphetamine use in this model will be based on the past 28 days prior to baseline, and since the previous assessment at the week 4, 8 and 12 assessments (up to 35 days prior to the assessment). Days when the participant was incarcerated or hospitalised will be censored, as per the TLFB guidelines. An exposure term (i.e. offset) will be included in the model to adjust for the exact number of days in each of these periods, because this can vary depending on the actual assessment dates and censoring.

Interpretation of the treatment effect from the model (i.e., interaction effect rate ratio): The main treatment effect will be the time (baseline vs. active medication phase) x condition (active vs. placebo) interaction effect, with the primary timepoint being week 12. This interaction effect represents the rate ratio of change in days of methamphetamine from baseline to follow-up (i.e., rate of days of use at baseline/rate of days of use at follow-up) for the active condition, over the rate ratio of change from baseline to follow-up for the placebo condition. That is:

r = (days of use at follow-up/days of exposure at follow-up) / (days of use at baseline/days of exposure at baseline)

r_p = r in placebo group

r_a = r in active group

The interaction coefficient representing the treatment effect is the rate ratio (rr) of these two parameters, i.e., r_a / r_p.

Thus, the interaction term can be interpreted as the relative difference in the rate of *change* between the intervention and control groups, which allows for random misbalance at baseline rather than assuming baseline methamphetamine use is the same in both groups.

Analysis of the primary outcome at week 20: A separate analysis will be conducted to compare days of methamphetamine use in the past 4 weeks at the week 20 assessment. We have chosen to analyse the week 20 endpoint separately because we believe that (a) differences in the conduct of the week 20 assessment may impact data variability (e.g., phone interviews and greater attrition), and (b) there are differences in the clinical interpretation of the outcome at the week 20 assessment (because

participants are no longer on the trial intervention and are offered off-label mirtazapine and other substance use treatment prior to week 20). We will test the main effect of the trial medication on days of methamphetamine use at week 20 using a mixed model with a group (placebo [0] vs. mirtazapine [1]) by time (baseline [0], week 20 [1]) interaction effect. A negative binomial generalised linear mixed model will be used to test this effect. An exposure term (i.e. offset) will be included in the model to adjust for the exact number of days in each of these periods if the observed number of days in the TLFB varies from 28 days. As noted under the general principles, a random intercept term will be included in the model to account for clustering on repeats [31], and a random intercept term will be included in the model to account for site only if this significantly improves model fit (based on a likelihood ratio test; $p < 0.05$).

6.3 Planned analysis of the secondary outcomes

The analysis of the secondary endpoints will be based on the intention-to-treat dataset and based on unimputed data. All tests will be two-sided with $p < 0.05$.

Abstinence (negative oral fluid samples)

Descriptive data on the proportion of negative oral fluid samples in each group across the active trial phase will be presented. The effect of mirtazapine on methamphetamine negative oral fluid samples (no [0], yes [1]) will be tested using a group contrast (placebo [0] vs. mirtazapine [1]) across all follow-up time points (weeks 4, 8 and 12) to obtain an average treatment effect across the 12-week intervention period. A generalised linear mixed model with a logistic link will be used to test this effect.

Depression

The effect of mirtazapine on depression will be tested using a mixed model using a group (placebo [0] vs. mirtazapine [1]) by time (baseline [0] vs. weeks 4 [1], week 8 [2], week 12 [3]) interaction, with time entered as a factor variable, producing individual effect estimates for each time point and making no assumptions about the linearity of changes over time. The primary timepoint for this endpoint is week 12. A generalised linear mixed model will be used to test this effect.

The effect of mirtazapine on depression at week 20 will be tested using a separate analysis. We will test the main effect of the trial medication on depression at week 20 using a mixed model with a group (placebo [0] vs. mirtazapine [1]) by time (baseline [0], week 20 [1]) interaction effect. A generalised linear mixed model will be used to test this effect. We have chosen to analyse the week 20 endpoint separately because we believe that (a) differences in the conduct of the week 20 assessment may impact data variability (e.g., phone interviews and greater attrition), and (b) there are differences in the clinical interpretation of the outcome at the week 20 assessment (because participants are no longer on the trial intervention and are offered off-label mirtazapine and other substance use treatment prior to week 20).

Other secondary endpoints (sleep, quality of life and HIV risk)

The effect of mirtazapine on other secondary endpoints (sleep, quality of life and HIV risk) will be tested with a mixed model using a group (placebo [0] vs. mirtazapine [1]) by time (baseline [0] vs. weeks 4 [1], week 8 [2] and 12 [3]) interaction, with time entered as a factor variable, producing individual effect estimates for each time point and making no assumptions about the linearity of

changes over time. The primary timepoint for these endpoints is week 12. A generalised linear mixed model will be used to test these effects.

6.4 Planned analysis of the tertiary and exploratory outcomes

Planned analyses of the tertiary outcomes of other substance use, anxiety, and patient impression will be based on the unimputed intention-to-treat dataset.

Other substance use: The effect of the trial medication on days of other substance use will be tested using a mixed model with a group (placebo [0] vs. mirtazapine [1]) by time (baseline [0], week 4 [1], week 8 [2], week 12 [3], week 20 [4]) interaction effect, with time entered as a factor variable, producing individual effect estimates for each time point and making no assumptions about the linearity of changes over time. The primary timepoint is week 12. A generalised linear mixed model will be used to test this effect.

Anxiety: The effect of mirtazapine on anxiety will be tested using a mixed model using a group (placebo [0] vs. mirtazapine [1]) by time (baseline [0] vs. weeks 4 [1], week 8 [2] and 12 [3]) interaction effect, with time entered as a factor variable, producing individual effect estimates for each time point and making no assumptions about the linearity of changes over time. The primary timepoint is week 12. A generalised linear mixed model will be used to test this effect.

Patient impression: The effect of mirtazapine on the PGI scale score will be tested a group contrast (placebo [0] vs. mirtazapine [1]) across all follow-up time points (weeks 4, 8 and 12) to obtain an average treatment effect across the 12-week intervention period. A generalised linear mixed model will be used to test this effect.

Adverse events: The analysis of adverse event data will be based on the safety dataset and include only adverse event data for the 12-week trial medication period (wk 0 to wk 12). The percentage of participants reporting adverse events and serious adverse events for each System Organ Class will be compared between conditions using a Pearson's Chi-Square test. For serious adverse events, expectedness and causality will be reported.

Suicidality: The analysis of suicidality will be based on the safety dataset and include only suicidality data for the 12-week trial medication period (wk 0 to wk 12). The percentage of participants who screened positive for suicide risk (CSSRS-S score of 3 or greater) during the 12-week trial medication period (wk 0 to wk 12) will be compared between conditions (placebo [0] vs. mirtazapine [1]) using a Pearson's Chi-Square test.

6.5 Sensitivity analyses

Sensitivity analyses will be conducted that imputes missing data for the analyses of the primary outcome of methamphetamine use days and for the secondary outcome of methamphetamine abstinence.

Sensitivity analysis may be conducted to test the assumption that the data are not missing at random for the primary outcome of methamphetamine use days and for the secondary outcome of methamphetamine abstinence. This will involve testing the impact of different assumed outcome values for missing data on the final treatment effect estimate.

6.6 Additional analyses

The following analyses may be done after the primary analysis to supplement the primary analyses or as separate research papers.

Subgroup analyses: Subgroup analyses are planned for the sub-groups of (1) depressed (PHQ-9 score 10 or greater at eligibility), (2) not depressed (PHQ-9 score of < 10 at eligibility), (3) participants who reported being male at birth, and (3) participants who reported being female or other sex at birth. The randomisation was stratified on these subgroups, meaning that participants are randomly assigned to condition within each subgroup.

Per-protocol analysis: A set of per-protocol analyses will be undertaken on the per-protocol dataset. This may be done for the primary and secondary end-points and for any sub-group analyses. The per protocol analysis will use non-imputed data. These models will incorporate adjustment to account for any group differences in baseline characteristics.

Treatment complier effect: A set of treatment complier analyses may be conducted on the treatment complier dataset. This may be done for the primary and secondary endpoints and for any sub-group analyses. Treatment complier analysis will incorporate adjustment to account for any group differences in baseline characteristics.

Average treatment effect: Additional analyses may be conducted that repeat the primary and secondary analyses but which collapse the follow-up data across the week 4, 8 and 12 follow-up time points (i.e., baseline [0] vs. week 4 [1], week 8 [1], week 12 [1]) such that the outcome is the average treatment effect across the 12-week trial medication period.

Covariate adjustment. Analyses of the primary outcome measure will be repeated with adjustment for a set of prognostic variables, as per the recommendations of Kahan et al. [32], to improve model precision and aid interpretation of the main analyses of the primary outcome. These prognostic variables will include: age, sex (male vs. female or other), methamphetamine use days in the past 4 weeks at eligibility, main route of methamphetamine administration (injecting vs. other routes), and PHQ-9 depression score at eligibility. All of these variables were collected at the eligibility assessment, prior to randomisation, and therefore they are not collider variables.

6.7 Exploratory analyses and embedded studies

Exploratory analyses that have been planned to-date are detailed in the protocol. No statistical analysis plan has been developed for these exploratory analyses. Nor have any other exploratory analyses had been planned at the time of writing this Statistical Analysis Plan.

If the intervention is effective, cost analyses will be undertaken. These will be detailed in a subsequent Health Economic Analysis Plan. Data collected to facilitate this analysis include: (1) the EQ-5D-5L[22] version 2.1 (2) the Work Productivity and Activity Impairment Questionnaire – General Health V2 (WPAI-GH), and (3) data on health service utilisation and criminal justice contact.

6.8 Missing data

Missing data will be imputed using multiple chained equations (fully conditional specification) in R. For imputed data on days of methamphetamine use in the past four weeks, the exposure time (offset) for any missing periods will be set at 28 days. Imputation models will include days of methamphetamine use in the past 28 days at the baseline assessment and any data on days of methamphetamine use from the observed follow-up assessments. For imputed data on

methamphetamine negative oral fluid samples, the number of assessments where oral fluid test results were missing will be included. Other variables related to attrition will be considered for inclusion in the imputation model. Based on Monte-Carlo error, the number of imputations used will be based on the percentage of cases with missing data, rounded up to the nearest 10 (e.g. for 15% missing data, M=20 imputations will be used [33]).

If adequate imputation models cannot be derived, the alternative strategy will be to use Inverse Probability of Censoring Weighting (IPCW) to adjust for differences between groups in baseline for baseline variables that are correlated with missingness.

Details of any imputation models will be included in online supplementary material on publication.

6.9 Statistical software

All analyses will be conducted in Stata Version 18.0 or later and R 4.4.3

7 Related documents

The Trial Protocol, Trial Masterfile, Statistical Masterfile and Data Management Plan are held at the University of New South Wales with the Coordinating Principal Investigator, Rebecca McKetin. Each site Principal Investigator holds a copy of the Masterfile.

8 Roles and responsibilities

Rebecca McKetin (Coordinating Principal Investigator)
Professor
National Drug and Alcohol Research Centre
Street address: 22-32 King Street Randwick
UNSW SYDNEY NSW 2052 AUSTRALIA
T: +61 (2) 9385 0294 / + 61 406538259
E: r.mcketin@unsw.edu.au

Philip Clare (Biostatistician)
Dr. Philip Clare – Research Fellow/Biostatician
National Drug and Alcohol Research Centre
Street address: 22-32 King Street Randwick
UNSW SYDNEY NSW 2052 AUSTRALIA
T: +61 (2) 9385 0333
E: p.clare@ unsw.edu.au

See study protocol for details of other study investigators.

9 Appendix

Demographic and substance use history collected at eligibility:

The eligibility assessment was conducted within the 4 weeks prior to randomisation. If this timeframe was exceeded, the assessment was updated to confirm eligibility.

The following variables relating to demographics and substance use history were assessed at the eligibility assessment.

Demographic variables:

- Age in years
- Sex at birth: male, female, other
- Current gender (missing for 18 participants): male, female, non-binary, I use a different term, prefer not to answer
- Sexual orientation: heterosexual, gay or lesbian, bisexual, other, unsure or prefer not to say
- Country of birth: Australia, other
- Main language spoken at home: English, other
- Years of completed schooling
- Qualifications: none, trade/technical, university
- Employment status: Full-time employment, part-time or casual employment, unemployed, student, home duties
- Current accommodation: Privately owned or rented house or flat, Boarding house/hostel/shelter or refuge, Drug treatment residence, No fixed address/homeless, Other
- Living arrangement: Alone, Partner/Spouse/De-facto, Children, Parent(s) or other relatives (grandparents/aunts etc), Non-related adult/flatmates, Other
- Net income in the past fortnight (all sources): Less than $200, $200-399, $400-799, $800-1199, $1200 or more
- Current marital status: Single, Married/De-facto, Separated, Divorced, Widowed
- Biological children: yes/no, if yes, how many under 16 years of age; how many under 16 years of age living with participant
- Prison history: Ever served a prison sentence
- Previous diagnosis of mental disorders: Mania or bipolar, Schizophrenia, Drug-induced psychosis, Other psychosis, Depression, Anxiety, ADHD, PTSD, Personality disorder, Other

Methamphetamine use variables:

- A DSM 5 diagnosis of moderate to severe methamphetamine use disorder made using questions from the Composite International Diagnostic Interview.
- Self-reported days of methamphetamine use in the past 28 days, assessed using the Timeline Followback (TLFB) method
- Age of first methamphetamine use
- Ever injected methamphetamine or any other drug
- Main route of methamphetamine administration in the past month (inject, smoke, other)
- Other routes of methamphetamine administration in the past month (inject, smoke, other

- Main form of methamphetamine used in the past month (crystalline, powder, other)

Treatment history

- Currently receiving treatment or other help for methamphetamine
- Methamphetamine treatment history: Ever started treatment for methamphetamine use (e.g., detox, rehab or drug counselling).
- Other help for methamphetamine use: Ever received other help for methamphetamine use (e.g., GP, counsellor, psychologist, psychiatrist, Narcotics Anonymous, online help).

Other measures

Other substance use: Self-reported days of other drug use in the past 28 days (all major drug classes: tobacco, alcohol, cannabis, heroin, other opioids, cocaine, ecstasy/MDMA, other hallucinogens, inhalants, benzodiazepines not prescribed, antidepressants not prescribed, antipsychotics not prescribed, 'other' drugs and other medications not prescribed). Substance use included only extra-medical use of substances.

- Depression: depressive symptoms in the past 2 weeks, assessed using the PHQ-9
- Suicidality: Suicidality risk in the month prior to the eligibility assessment was assessed using the CSSRS-S. The timeframe of this assessment could be extended to the past year to confirm eligibility.
- Concomitant medications: Current concomitant medications were assessed at eligibility and recorded from 14 days prior to the eligibility assessment.
- Self-reported height and weight
- Measured height and weight

Primary and secondary outcome assessments:

The primary and secondary outcomes were assessed at baseline, week 4, week 8 and week 12. These assessments were scheduled 28 days apart, but the assessment date could vary from seven days prior to the due date until 21 days after the due date. In addition, these assessments could be scheduled no sooner than 7 days after the previous assessment.

Primary outcome:

The primary outcome was self-reported days of methamphetamine use assessed using the Timeline Followback (TLFB) [17] for the past 4 weeks at Baseline and updated at the week 4, week 8 and week 12 assessments for the period since the previous assessment (or up to 35 days, if the previous assessment was more than 35 days earlier). Days when a participant is incarcerated or in hospital (inpatient only) are censored from the TLFB [17].

Secondary outcomes:

1. *Abstinence from methamphetamine use* was assessed at weeks 4, 8 and 12 by taking oral fluid samples from participants, using a commercial oral fluid collection device, and analysing samples for methamphetamine and amphetamine concentration. A methamphetamine-negative oral fluid

sample was defined as having < 25 ng/mL methamphetamine. Oral fluid is a sensitive and stable medium for the detection of methamphetamine and correlates highly with plasma tests [18].

2. *Depressive symptoms* in the past 2 weeks were assessed at baseline, week 4, week 8 and week 12 using the Patient Health Questionnaire-9 (PHQ-9) [19] The PHQ-9 is a brief 9-item questionnaire that has excellent internal consistency (Cronbach's alpha of 0.89), test-retest reliability (r = 0.84) and good construct validity against other measures [19]
3. *Sleep quality* was assessed in the past month at baseline, week 4, week 8 and week 12 using the Athens Insomnia Scale (AIS) [20]. The AIS is an 8-item self-report measure which has 0.9 test-retest reliability and has been validated against other measures of sleep quality [20].
4. *HIV risk behaviour* was assessed in the past month at baseline, week 4, week 8 and week 12 using a modified version of the HIV Risk-taking Behaviour Scale (HRBS) from the Opioid Treatment Index [21]. The HRBS is a validated and reliable scale that provides a composite risk index for injecting and sexual behaviour. The modified version of this scale is available from the authors on request.
5. *Quality of life* was assessed at baseline, week 4, week 8 and week 12 using the EuroQol-5D-5L (EQ-5D) [22] which is based on the participants perception of their health on that day.

Tertiary outcomes:

1. *Other substance use*: Self-reported days of use in the past 28 days at baseline, week 4, week 8 and week 12 was assessed for all major drug classes (alcohol, tobacco, cannabis, heroin, cocaine, ecstasy, hallucinogens, inhalants, other) and extra medical use of prescribed psychotropic drugs (categorised as opioids, benzodiazepines, antidepressants, antipsychotics, other).
2. *Anxiety* was assessed in the past 2 weeks at baseline, week 4, week 8 and week 12 using the Generalized Anxiety Disorder – 7 Item (GAD-7) [25].
3. *Suicidality* was assessed at eligibility (for the past month), and since the last assessment at baseline, week 4, week 8 and week 12, using the Columbia Suicide Severity Rating Scale Screener (CSSRS-S) [23, 24]. This instrument was used to monitor suicidality throughout the study. A score of 3 or more on the CSSRS-S predicts a significant increase in the risk of a subsequent suicide attempt (28).
4. *Participants impression on health status* was assessed using the single-item Patient Global Impression – Improvement (PGI - I) [26], which reflects participant's view on whether their health has improved or worsened since the start of the study. This single-item scale was administered at weeks 4, 8 and 12.

Exploratory outcomes:

5. *Adverse events (AEs)**:*** Adverse events were assessed using open ended prompts about problems with the participant's health and problems with the study medication. All AEs were logged using a structured form that included information on date of onset and completion, severity, treatment received, relatedness to the trial medication, and whether the AE was a serious AE (SAE). AEs were assessed at week 2 after starting the trial medication; AEs were reviewed and updated at each assessment thereafter. AEs also include events that were identified outside this assessment process (e.g., at medical assessments) with these recorded at the nearest completed assessment. All AEs were coded by system organ classification according to the Medical Dictionary for Regulatory Activities (MedDRA)[30]
6. *Height and weight:* Self-reported height and weight were recorded at the week 12 assessment. Actual height and weight were re-assessed at the final medical assessment (week 18) when this was done in person.
7. *Treatment satisfaction* was assessed at week 12 using Treatment Satisfaction Questionnaire for Medication Version II (TSQM II) [27] which assesses medication convenience, side-effects and effectiveness for the previous 2-3 weeks (or since the participant had last used the medication, if

they had ceased medication). The TSQM II provides a global satisfaction summary score and subscale scores for medication convenience, side-effects, and effectiveness.

Unpublished questions on medication tolerability: Additional questions were included at weeks 4 and 8 to assess medication tolerability and adverse reactions to mirtazapine. These included a set of 5 generic questions about participants' perceived medication impacts and 7 questions specific to the expected effects of mirtazapine. These questions did not have a timeframe (they rated the participant's current beliefs and attitudes). At week 2 and week 16 a single question was included asking participants whether they had experienced any adverse reactions to mirtazapine, and withdrawal effects from ceasing mirtazapine, respectively. A final question asked participants whether the participant thought they were receiving the placebo (dummy pill) or active medication (mirtazapine) with the options of don't know, placebo (dummy pill), active medication (mirtazapine).

Work Productivity and Activity Impairment Questionnaire – General Health (WPAIQ-GH) V2 *[29]* was administered at baseline and week 12; this questionnaire asks about current work status and the impact of health problems on work productivity in the past 7 days.

8. *Health service use* questions administered at baseline ask about health service use in the past 3 months; these questions were repeated at week 12 when they asked about health service use since starting the trial medication (i.e., since baseline). These questions included (a) treatment for methamphetamine or other drug use, by counselling [number of sessions], rehabilitation [number of times started and total days spent in rehabilitation], detoxification [number of times started and total days spent in detoxification], and other drug treatment [number of times started]), (b) occasions of care from the following: ambulance, emergency department, general practitioner, counsellor or psychologist, psychiatrist, dentist, other health service; (c) total number of hospitalisations (including psychiatric hospital admissions), (d) number of arrests, (e) number of court appearances, and (f) how many days detained in a lock-up.
9. *Concomitant medications:* All medications taken by participants during the trial were recorded on a template adapted from the National Institute of Health Concomitant Medications Form [28]. These were assessed at eligibility and recorded from 2 weeks prior to the eligibility assessment. The concomitant medication form was reviewed and updated at each subsequent assessment. Information recorded for each medication included: name of the medication, start date (recorded from 14 days prior to the eligibility assessment), whether the medication use was ongoing or complete, the dose unit, the total dose per day, frequency of dosing, route and reason for medication. All medications were coded against the REDCap dictionary of WHO generic medications.

<u>Post-intervention assessments:</u>

A phone assessment was done at week 20 to assess outcomes after ceasing the trial intervention. Outcome measures assessed at week 20 are listed below. Details can be found in Tables 1 and 2. The measures were the same as those described in the sections above.

- Days of methamphetamine use in the past 28 days, assessed using the TLFB
- Self-reported days of other drug use in the past 28 days, by major drug class
- Depressive symptoms in the past 2 weeks were assessed using the PHQ-9
- Suicidality since the previous assessment (week 12) was assessed using the CSSRS-S
- Concomitant medications since the previous assessment
- Adverse events since the previous assessment
- Health service use since the previous (week 12) assessment
- Tolerability/Satisfaction TSQM-II

10 References

1. Degenhardt L, Larney S, Dobbins T, Chan G, Weier M, Roxburgh A et al. Estimating the number of regular and dependent methamphetamine users in Australia, 2002-2014. Medical Journal of Australia. In press;
2. Strickland JC, Stoops WW, Dunn KE, Smith KE, Havens JR. The continued rise of methamphetamine use among people who use heroin in the United States. Drug Alcohol Depend. 2021;225:108750.
3. Mcketin R, Najman J, Baker A, Lubman D, Dawe S, Ali R et al. Evaluating the impact of community-based treatment options on methamphetamine use: findings from the Methamphetamine Treatment Evaluation Study (MATES). Addiction. 2012;107:1998-2008
4. Farrell M, Martin NK, Stockings E, Bórquez A, Cepeda JA, Degenhardt L et al. Responding to global stimulant use: challenges and opportunities. Lancet. 2019;394:1652-67.
5. Mcketin R, Degenhardt L, Shanahan M, Baker AL, Lee NK, Lubman DI. Health service utilisation attributable to methamphetamine use in Australia: Patterns, predictors and national impact. Drug Alcohol Rev. 2018;37:196-204.
6. Han B, Compton WM, Jones CM, Einstein EB, Volkow ND. Methamphetamine Use, Methamphetamine Use Disorder, and Associated Overdose Deaths Among US Adults. JAMA Psychiatry. 2021;78:1329-42.
7. Brensilver M, Heinzerling KG, Shoptaw S. Pharmacotherapy of amphetamine-type stimulant dependence: An update. Drug and Alcohol Review. 2013;32:449-60.
8. Colfax G, Santos G-M, Chu P, Vittinghoff E, Pluddemann A, Kumar S et al. Amphetamine-group substances and HIV. Lancet. 2010;376:458-74.
9. Ezard N, Dunlop A, Clifford B, Bruno R, Carr A, Bissaker A et al. Study protocol: A dose-escalating, phase-2 study of oral lisdexamfetamine in adults with methamphetamine dependence. BMC Psychiatry. 2016;16:
10. Trivedi MH, Walker R, Ling W, Dela Cruz A, Sharma G, Carmody T et al. Bupropion and Naltrexone in Methamphetamine Use Disorder. N Engl J Med. 2021;384:140-53.
11. Mcketin R, Dean OM, Turner A, Kelly PJ, Quinn B, Lubman DI et al. N-acetylcysteine (NAC) for methamphetamine dependence: A randomised controlled trial. EClinicalMedicine. 2021;38:101005.
12. Sharafi H, Bakouni H, Mcanulty C, Drouin S, Coronado-Montoya S, Bahremand A et al. Prescription psychostimulants for the treatment of amphetamine-type stimulant use disorder: A systematic review and meta-analysis of randomized placebo-controlled trials. Addiction. 2024;119:211-24.
13. Shoptaw S, Huber A, Peck J, Yang X, Liu J, Jeff D et al. Randomized, placebo-controlled trial of sertraline and contingency management for the treatment of methamphetamine dependence. Drug Alcohol Depend. 2006;85:12-8.
14. Colfax GN, Santos GM, Das M, Santos DM, Matheson T, Gasper J et al. Mirtazapine to reduce methamphetamine use: A randomized controlled trial. Archives of General Psychiatry. 2011;68:1168-75.
15. Coffin PO, Santos GM, Hern J, Vittinghoff E, Walker JE, Matheson T et al. Effects of Mirtazapine for Methamphetamine Use Disorder Among Cisgender Men and Transgender Women Who Have Sex With Men: A Placebo-Controlled Randomized Clinical Trial. JAMA Psychiatry. 2019;
16. Naji L, Dennis B, Rosic T, Wiercioch W, Paul J, Worster A et al. Mirtazapine for the treatment of amphetamine and methamphetamine use disorder: A systematic review and meta-analysis. Drug Alcohol Depend. 2022;232:109295.
17. Fals-Stewart W, O'farrell TJ, Freitas TT, Mcfarlin SK, Rutigliano P. The timeline followback reports of psychoactive substance use by drug-abusing patients: psychometric properties. J Consult Clin Psychol. 2000;68:134-44.
18. Huestis MA, Cone EJ. Methamphetamine Disposition in Oral Fluid, Plasma, and Urine. Annals of the New York Academy of Sciences. 2007;1098:104-21.

19. Kroenke K, Spitzer RL, Williams JB. The PHQ-9: validity of a brief depression severity measure. J Gen Intern Med. 2001;16:606-13.
20. Soldatos CR, Dikeos DG, Paparrigopoulos TJ. Athens Insomnia Scale: validation of an instrument based on ICD-10 criteria. J Psychosom Res. 2000;48:555-60.
21. Darke S, Hall W, Wodak A, Heather N, Ward J. Development and validation of a multi-dimensional instrument for assessing outcome of treatment among opiate users: the Opiate Treatment Index. British Journal of Addiction. 1992;87:733-42.
22. Herdman M, Gudex C, Lloyd A, Janssen M, Kind P, Parkin D et al. Development and preliminary testing of the new five-level version of EQ-5D (EQ-5D-5L). Qual Life Res. 2011;20:1727-36.
23. Posner K, Brent D, Lucas C, Gould M, Stanley B, Brown G et al. Columbia-Suicide severity rating scale (C-SSRS) Version 6. New York, 2008. New York; 2008.
24. Bjureberg J, Dahlin M, Carlborg A, Edberg H, Haglund A, Runeson B. Columbia-Suicide Severity Rating Scale Screen Version: initial screening for suicide risk in a psychiatric emergency department. Psychol Med. 2021;1-9.
25. Spitzer RL, Kroenke K, Williams JB, Löwe B. A brief measure for assessing generalized anxiety disorder: the GAD-7. Arch Intern Med. 2006;166:1092-7.
26. Mohebbi M, Dodd S, Dean OM, Berk M. Patient centric measures for a patient centric era: Agreement and convergent between ratings on The Patient Global Impression of Improvement (PGI-I) scale and the Clinical Global Impressions - Improvement (CGI-S) scale in bipolar and major depressive disorder. Eur Psychiatry. 2018;53:17-22.
27. Atkinson MJ, Sinha A, Hass SL, Colman SS, Kumar RN, Brod M et al. Validation of a general measure of treatment satisfaction, the Treatment Satisfaction Questionnaire for Medication (TSQM), using a national panel study of chronic disease. Health and Quality of Life Outcomes. 2004;2:12-12.
28. NCCIH Clinical Research Toolbox, Bethesda, MD: National Institute for Health, National Centre for Complementary and Integrative Health; 2018.
29. Reilly MC, Zbrozek AS, Dukes EM. The validity and reproducibility of a work productivity and activity impairment instrument. Pharmacoeconomics. 1993;4:353-65.
30. Brown EG, Wood L, Wood S. The medical dictionary for regulatory activities (MedDRA). Drug Saf. 1999;20:109-17.
31. Gueorguieva R, Krystal JH. Move over ANOVA: progress in analyzing repeated-measures data and its reflection in papers published in the Archives of General Psychiatry. Archives of General Psychiatry. 2004;61:310-7.
32. Kahan BC, Jairath V, Doré CJ, Morris TP. The risks and rewards of covariate adjustment in randomized trials: an assessment of 12 outcomes from 8 studies. Trials. 2014;15:139.
33. White IR, Royston P, Wood AM. Multiple imputation using chained equations: Issues and guidance for practice. Stat Med. 2011;30:377-99.
