# Supplementary material for: Mirtazapine for Methamphetamine Use Disorder: A Randomized Clinical Trial
Source: JAMA Psychiatry. 2026 Apr 1;83(6):581–9. doi: 10.1001/jamapsychiatry.2026.0159 (PMC13044789; doi:10.1001/jamapsychiatry.2026.0159)
Supplement: Supplement 3. — eMethods. Supplemental Methods eFigure 1. Estimated Mean Days of Methamphetamine Use by Condition From the ITT Model eFigure 2. Estimated Mean Days of Methamphetamine Use by Condition From the ITT Model Adjusted for Baseline Covariates eFigure 3. Estimated Means by Whether Participants Were Depressed for (A) Methamphetamine Use Days, (B) Depression, and (C) Insomnia eTable 1. Summary of Missing Data in Each Analysis and Auxiliary Variable eTable 2. Baseline Correlates of the Number of Missed Follow-Up Assessments (Weeks 4, 8, and 12) eTable 3. Participant Characteristics at Eligibility by Condition eTable 4. Primary, Secondary, and Tertiary End Points for the 12-Week Intention-to-Treat Estimand eTable 5. Model Output for the Primary End Point for the Intention-to-Treat Analysis eTable 6. Estimated Mean (95% CI) Days of Methamphetamine Use Extracted From the Intention-to-Treat Analysis eTable 7. Model Output for the Primary End Point for the Intention-to-Treat Analysis With Imputed Missing Data eTable 8. Model Output for the Primary End Point for the Intention-to-Treat Analysis With Adjustment for Baseline Covariates eTable 9. Estimated Mean (95% CI) Days of Methamphetamine Use Extracted From the Intention-to-Treat Analysis Adjusted for Baseline Covariates eTable 10. Primary and Secondary End Points for the Intention-to-Treat Estimand for Participants Who Were Male at Birth eTable 11. Primary and Secondary End Points for the Intention-to-Treat Estimand for Participants Who Were Female or Other at Birth eTable 12. Primary and Secondary End Points for the Intention-to-Treat Estimand for Participants Who Were Not Depressed at Eligibility eTable 13. Primary and Secondary End Points for the Intention-to-Treat Estimand for Participants Who Were Depressed at Baseline eTable 14. Adverse Events Within Each System Organ Classification (SOC) by Condition (From Baseline to Week 12) eTable 15. Adverse Events by MedDRA Diagnosis by Condition eTable 16. Serious Adverse Events by Co [file jamapsychiatry-e260159-s003.pdf]

Supplemental Online Content

McKetin R, Shoptaw S, Saunders L, et al. Mirtazapine for methamphetamine use disorder: a randomized clinical trial. *JAMA Psychiatry*. Published online April 1, 2026. doi:10.1001/jamapsychiatry.2026.0159

eMethods. Supplemental Methods

eFigure 1. Estimated Mean Days of Methamphetamine Use by Condition From the ITT Model

eFigure 2. Estimated Mean Days of Methamphetamine Use by Condition From the ITT Model Adjusted for Baseline Covariates

eFigure 3. Estimated Means by Whether Participants Were Depressed for (A) Methamphetamine Use Days, (B) Depression, and (C) Insomnia

eTable 1. Summary of Missing Data in Each Analysis and Auxiliary Variable

eTable 2. Baseline Correlates of the Number of Missed Follow-Up Assessments (Weeks 4, 8, and 12)

eTable 3. Participant Characteristics at Eligibility by Condition

eTable 4. Primary, Secondary, and Tertiary End Points for the 12-Week Intention-to-Treat Estimand

eTable 5. Model Output for the Primary End Point for the Intention-to-Treat Analysis

eTable 6. Estimated Mean (95% CI) Days of Methamphetamine Use Extracted From the Intention-to-Treat Analysis

eTable 7. Model Output for the Primary End Point for the Intention-to-Treat Analysis With Imputed Missing Data

eTable 8. Model Output for the Primary End Point for the Intention-to-Treat Analysis With Adjustment for Baseline Covariates

eTable 9. Estimated Mean (95% CI) Days of Methamphetamine Use Extracted From the Intention-to-Treat Analysis Adjusted for Baseline Covariates

eTable 10. Primary and Secondary End Points for the Intention-to-Treat Estimand for Participants Who Were Male at Birth

eTable 11. Primary and Secondary End Points for the Intention-to-Treat Estimand for Participants Who Were Female or Other at Birth

© 2026 McKetin R et al. *JAMA Psychiatry*.

eTable 12. Primary and Secondary End Points for the Intention-to-Treat Estimand for Participants Who Were Not Depressed at Eligibility

eTable 13. Primary and Secondary End Points for the Intention-to-Treat Estimand for Participants Who Were Depressed at Baseline

eTable 14. Adverse Events Within Each System Organ Classification (SOC) by Condition (From Baseline to Week 12)

eTable 15. Adverse Events by MedDRA Diagnosis by Condition

eTable 16. Serious Adverse Events by Condition

eReferences.

This supplemental material has been provided by the authors to give readers additional information about their work.

© 2026 McKetin R et al. *JAMA Psychiatry*.

eMethods. Supplemental Methods

1.1 INCLUSION AND EXCLUSION CRITERIA

Inclusion criteria

- Aged between 18 and 65 years
- Moderate to severe methamphetamine use disorder in the past year (DSM-5 past year diagnosis confirmed at Eligibility Assessment using modified version of the Composite International Diagnostic Interview)
- Current methamphetamine use (defined as using at least twice weekly in past 4 weeks based on the participant's self-reported use, and a positive drug screening test for methamphetamine)
- Willing to use effective contraception (for women only)
- Willing to provide contact details for their treating physician
- Willing to provide contact details for follow-up
- Able to provide informed consent and able to comply with both the requirements of the informed consent and the treatment protocol

Exclusion criteria

- In need of acute care (e.g., suicidality or acute psychosis, unstable psychiatric condition; medical detoxification)
- Pregnant or lactating
- Incarceration or current inpatient treatment (including residential rehabilitation, inpatient detoxification); this applies to the status of the participant at trial enrolment and does not preclude the participant from entering treatment or receiving usual care during the trial
- Currently taking prescribed antidepressant medication
- Any use of monoamine oxidase inhibitors in the 14 days prior to starting the trial medication
- Contraindications for mirtazapine, including:
  - Known hypersensitivity to mirtazapine
  - Use of antidepressant medication (including monoamine oxidase inhibitors, St. John's Wort, or SSRIs) other serotonergic drugs.
  - Galactose intolerance, Lapp lactase deficiency or glucose-galactose malabsorption (lactose is an excipient in the trial medication)
- High risk of adverse reactions to mirtazapine including suicide, overdose, sudden cardiac death, risk of agranulocytosis, or accidents and injuries from motor impairment
- Past year suicide attempt
- Unable or unwilling to avoid pregnancy during the trial (for both men and women)
- Participation in another clinical trial

1.2 ADDITIONAL EXPLORATORY ENDPOINTS

Concomitant medications: All medication received by participants will be recorded on a template adapted from the National Institute of Health Concomitant Medications Form. [1]

Health Economics data were collected to facilitate any subsequent economic evaluation. This data included the EuroQol V2.1, [2] the Work Productivity and Activity Impairment Questionnaire – General Health V2, [3] and contact with health services and the criminal justice system.

The *participant's impression* was assessed using the Patient Global Impression (PGI) scale. [4]

© 2026 McKetin R et al. *JAMA Psychiatry*.

Treatment satisfaction was assessed with the Treatment Satisfaction Questionnaire for Medication II (TSQM-II)[5].

Anxiety was assessed using the Generalised Anxiety Disorder 7 Item (GAD – 7) Scale.

Post Trial outcomes: At week 20 we assessed Adverse Events, health service use, days of methamphetamine use in0.001 the past 28 days, days of other drug use in the past 28 days, and depression using the PHQ-9.

1.3 IMPUTATION METHODS

There were two main mechanisms for missing data in the TINA trial:

1. Loss to follow-up;
2. Intermittent missingness due to refusal to answer, not knowing, etc;

with the main mechanism of missing data in the analysis variables being loss to follow-up.

Following the framework for the treatment of missing data proposed by the STRATOS initiative,[6] we conducted a sensitivity analysis using multiple imputation. The amount of missing data in each analysis and auxiliary variable, and the patterns of missingness, are shown in Table S1.

We imputed the data using chained equations with the data in long form, using the R package 'mice'[7]. To handle possible complexity in the data, we imputed all variables using random forests, from the package 'ranger',[8] except for the outcome variable, which we imputed using random intercept negative binomial regression, the same type of model as the outcome model, in order to ensure that all features of the outcome models (including the random intercept of individuals) was included in the imputation models. Based on the proportion of missing information in the data, we used M=20 imputations.[9] All variables used in the analysis models were included in imputation, as well as a number of auxiliary variables that might be related to missingness (Table S2).[6]

We then re-ran the primary analysis on each imputed dataset and combined the results using Rubin's rules.

© 2026 McKetin R et al. *JAMA Psychiatry*.

eFigure 1. Estimated Mean Days of Methamphetamine Use by Condition From the ITT Model

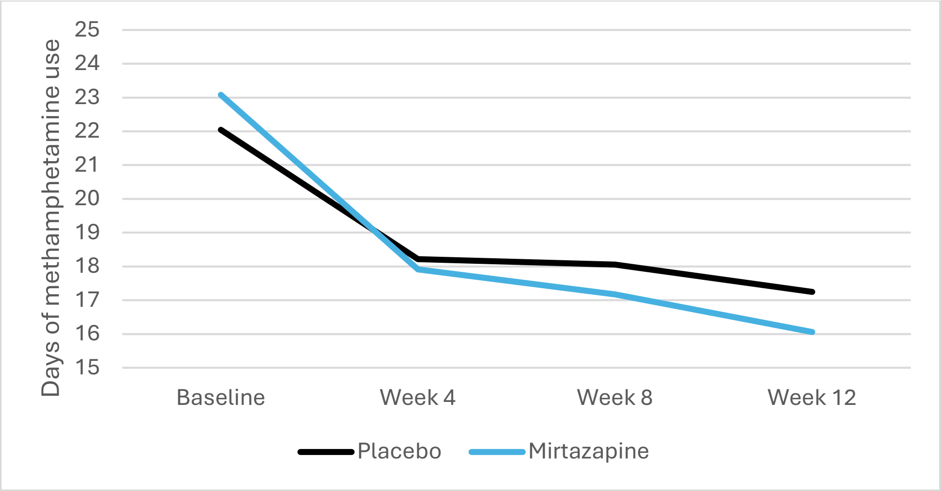

© 2026 McKetin R et al. *JAMA Psychiatry*.

eFigure 2. Estimated Mean Days of Methamphetamine Use by Condition From the ITT Model Adjusted for Baseline Covariates

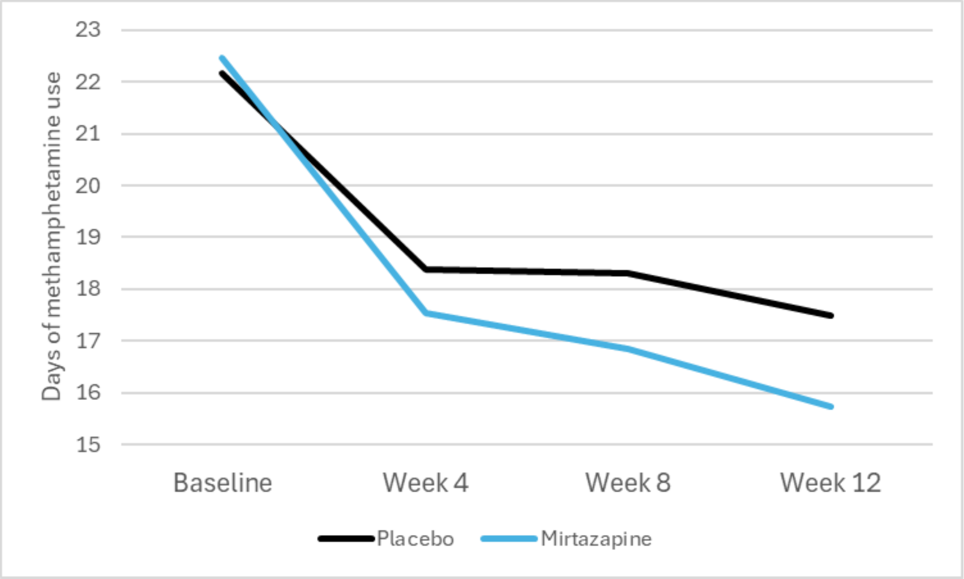

© 2026 McKetin R et al. *JAMA Psychiatry*.

eFigure 3. Estimated Means by Whether Participants Were Depressed for (A) Methamphetamine Use Days, (B) Depression, and (C) Insomnia

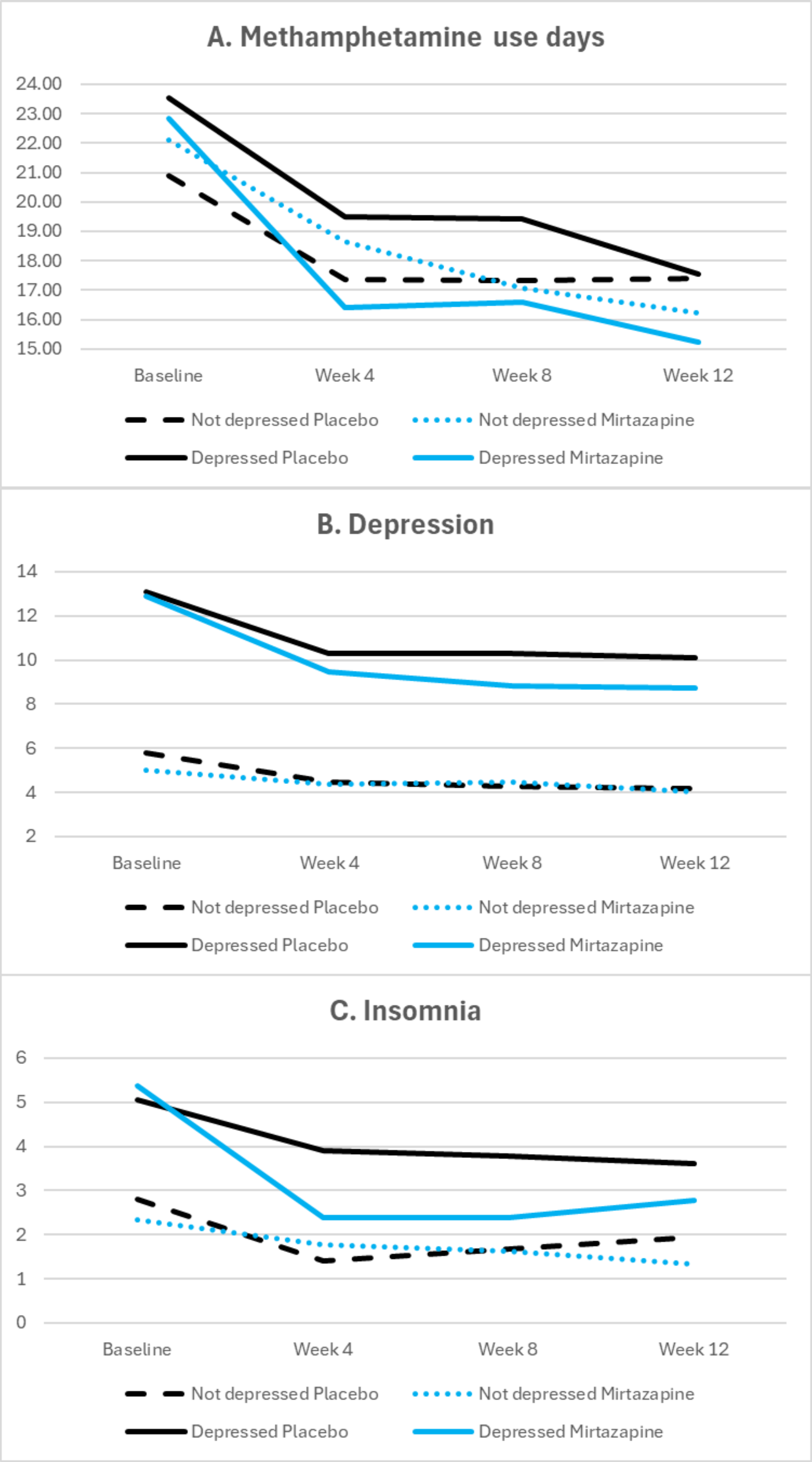

© 2026 McKetin R et al. *JAMA Psychiatry*.

eTable 1. Summary of Missing Data in Each Analysis and Auxiliary Variable

Variable	Patterns of missing data											n missing in variable
	**(1)**	**(2)**	**(3)**	**(4)**	**(5)**	**(6)**	**(7)**	**(8)**	**(9)**	**(10)**	**(11)**	
Assessment date	X	X										109
PHQ-Score	X									X		88
Marital status				X								4
TLFB Days of methamphetamine use	X	X										109
EQ-5D utilitiy score	X	X			X	X		X		X	X	120
AIS-5 insomnia score	X	X						X	X	X	X	115
Modified OTI HRBS score	X	X				X		X		X	X	116
BMI			X									8
GAD score	X	X					X	X		X	X	116
Polysubstance use in past 4 weeks	X	X									X	110
Years of completed education												0
Ever been in prison												0
Received help for methamphetamine use												0
Intervention group												0
event												0
Site												0
Age												0
Sex												0
Borth outside of Australia												0
Living situation												0
Number of biological children												0
Qualifications												0
Employment category												0
Income category												0
Current housing												0
Duration of methamphetamine use												0
Injecting methamphetamine use												0
History of specialist AOD treatment												0
TLFB Days observed												0
**Number in pattern**	87	22	8	4	4	3	3	2	2	1	1	

Note: X signifies that a particular variable was missing in that ‘pattern’ of missing data.

© 2026 McKetin R et al. *JAMA Psychiatry*.

eTable 2. Baseline Correlates of the Number of Missed Follow-Up Assessments (Weeks 4, 8, and 12)

	Condition	
	Spearman's Rho	P value
**Age in years**	**-0.13**	**0.013**
Male	0.02	0.788
Born outside of Australia	-0.02	0.706
Married or de-facto	-0.02	0.694
Have you ever been in prison (i.e., served a prison sentence)?	-0.02	0.673
How many years of school did you complete?	-0.04	0.437
Tertiary education		
No tertiary education	-0.04	0.523
Trade or technical qualification	0.06	0.235
University degree	-0.06	0.289
Employment		
Unemployed	0.02	0.710
Full-time employment	-0.04	0.488
Other employment (part-time, casual, student or home duties)	0.01	0.783
Income category		
Less than $800	0.05	0.357
$800-1199	-0.03	0.612
$1200 or more	-0.02	0.673
Living arrangement		
Living alone	-0.09	0.080
Living with children	-0.02	0.736
Living with spouse/partner	-0.03	0.567
**Living with other family**	**0.17**	**0.003**
Living with unrelated adults	-0.03	0.645
Accommodation		
Privately owned or rented	0.05	0.336
**Public housing**	**-0.11**	**0.027**
Other (e.g., boarding house, shelter, caravan, no fixed address)	0.07	0.213
Children		
No children	-0.05	0.369
1 child	0.02	0.755
2 children	0.04	0.508
Three or more children	0.02	0.708
Body Mass Index	-0.03	0.534
Methamphetamine treatment history		
Specialist drug treatment	-0.02	0.740
Other professional help	0.05	0.408
Either specialist AOD treatment or other professional help	0.02	0.655
Methamphetamine use		
Injecting main route of use	0.00	0.979
Duration of use (years)	-0.09	0.105
Days of use in past 4 weeks	0.02	0.680
Polysubstance use in the past 4 weeks	-0.06	0.247
Other measures		
Depression (PHQ-Score)	0.07	0.183
HIV risk (Score on the modified OTI HRBS)	0.05	0.336
Insomnia (AIS-5 score)	-0.05	0.387
Quality of life (EQ-5D utility score)	-0.05	0.317
Anxiety (GAD anxiety score)	0.05	0.357

© 2026 McKetin R et al. *JAMA Psychiatry*.

	Condition	
	Spearman's Rho	P value
Site	-0.09	0.112
Adelaide, n (%)	0.05	0.386
Brisbane, n (%)	-0.02	0.734
**Geelong, n (%)**	**0.16**	**0.007**
Perth, n (%)	0.01	0.882
Townsville, n (%)	-0.05	0.422
**Wollongong, n (%)**	**-0.13**	**0.010**
Condition allocation, n (%)	-0.02	0.748
Date of Assessment, n (%)	-0.09	0.112

© 2026 McKetin R et al. *JAMA Psychiatry*.

eTable 3. Participant Characteristics at Eligibility by Condition

	Placebo (n = 167)	Mirtazapine (n = 172)	P value	Total (N = 339)
Demographics				
Age, mean years (SD)	41.9 (8.3)	42.1 (8.9)	0.790	42.0 (8.6)
Male, n (%)	106 (63)	107 (62)	0.810	213 (63)
Immigrant (born outside of Australia), n (%)	27 (16)	28 (16)	0.978	55 (16)
Married or de-facto, n (%)	38 (23)	31 (18)	0.267	69 (20)
Prison history, n (%)	66 (40)	70 (41)	0.825	136 (40)
Schooling, median years (IQR)	10 (10-12)	10 (10-12)	0.952	10 (10-12)
Employment, n (%)				
Unemployed	90 (54)	89 (52)	0.692	179 (53)
Full-time employment	39 (23)	39 (23)	0.882	78 (23)
Other employment[a]	38 (23)	44 (26)	0.543	82 (24)
Income, n (%)				
Less than $800	64 (38)	57 (33)	0.319	121 (36)
$800-1199)	40 (24)	55 (32)	0.100	95 (28)
$1200 or more	63 (38)	60 (35)	0.587	123 (36)
Education, n (%)				
No tertiary education	49 (29)	59 (34)	0.327	108 (32)
Trade or technical qualification	106 (63)	100 (58)	0.315	206 (61)
University degree	12 (7)	13 (8)	0.896	25 (7)
Living situation,[b] n (%)				
Living alone	43 (26)	49 (28)	0.571	92 (27)
Living with children	55 (33)	52 (30)	0.593	107 (32)
Living with spouse/partner	42 (25)	38 (22)	0.508	80 (24)
Living with other family	34 (20)	38 (22)	0.696	72 (21)
Living with unrelated adults	33 (20)	36 (21)	0.789	69 (20)
Accommodation, n (%)				
Privately owned or rented	124 (74)	119 (69)	0.301	243 (72)
Public housing	31 (19)	32 (19)	0.992	63 (19)
Other[c]	12 (7)	21 (12)	0.119	33 (10)
Children,[d] n (%)				
No children	77 (46)	94 (55)	0.116	171 (50)
1 child)	51 (31)	39 (23)	0.101	90 (27)
2 children	21 (13)	24 (14)	0.708	45 (13)
3 or more children	18 (11)	15 (9)	0.523	33 (10)
Body Mass Index, mean (SD)	26.8 (5.8)	26.8 (5.7)	0.991	26.8 (5.8)
Methamphetamine treatment history, n (%)				
Drug treatment	68 (41)	86 (50)	0.086	154 (45)
Other professional help (e.g. GP, counsellor)	67 (40)	63 (37)	0.509	130 (38)
Either drug treatment or other professional help	91 (54)	98 (57)	0.645	189 (56)
Methamphetamine use at eligibility				
Days of use in the past 4 weeks,[e] median (IQR)	24 (17-28)	24 (20-28)	0.634	24 (18-28)
Injecting,[f] n (%)	81 (49)	74 (43)	0.311	155 (46)
Duration of use, mean years (SD)	20.7 (8.7)	20.9 (9.3)	0.851	20.8 (9.0)

[a] Part-time, casual, student or home duties

[b] Multiple responses could be selected

[c] For example: boarding house, shelter, caravan, no fixed address

[d] Includes biological children only

[e] Censoring days incarcerated or hospitalised

[f] The remaining participants either smoked (n = 178) or used other non-parenteral routes of administration (n = 6)

eTable 4. Primary, Secondary, and Tertiary End Points for the 12-Week Intention-to-Treat Estimand

	Placebo (n = 167)	Mirtazapine (n = 172)	Difference between mirtazapine and placebo (95% CI)
**Primary endpoint**			
Mean change in days of methamphetamine use – (95% CI)	-4.8 (-6.2 – -3.4)	-7.0 (-8.5 – -5.6)	-2.2 (-4.2 – -0.2)
**Secondary endpoints**			
Percent methamphetamine negative oral fluid – (95% CI)	12.0 (7.5 – 16.4)	13.2 (8.8 – 17.7)	1.3 (-4.7 – 7.2)
Mean change in PHQ-9 score – (95% CI)	-2.3 (-3.2 – -1.5)	-2.5 (-3.3 – -1.7)	-0.2 (-1.3 – 1.0)
Mean change in AIS insomnia score – (95% CI)	-1.2 (-1.7 – -0.6)	-1.8 (-2.3 – -1.2)	-0.6 (-1.4 – 0.2)
Mean change in HIV risk behaviour score – (95% CI)	-1.1 (-1.6 – -0.6)	-1.4 (-1.9 – -1.0)	-0.3 (-1.0 – 0.3)
Mean change in EQ5D quality of life utility score – (95% CI)	1.3 (-1.6 – 4.1)	2.2 (-0.6 – 5.0)	0.9 (-3.1 – 4.9)
**Tertiary endpoints**			
Mean change in days of other substance use	-1.4 (-3.2 – 0.3)	-0.8 (-2.5 – 0.9)	0.6 (-1.8 – 3.1)
Mean change in GAD anxiety score	-1.6 (-2.4 – -0.8)	-1.6 (-2.4 – -0.9)	-0.0 (-1.1 – 1.0)
Mean PGI score during treatment[a]	3.6 (3.5 – 3.7)	3.5 (3.3 – 3.6)	-0.2 (-0.3 – 0.0)
Mean change in body mass index[b] – 95% CI	0.4 (0.2 – 0.7)	0.4 (0.1 – 0.7)	-0.1 (-0.5 – 0.3)

[a] Lower scores represent greater improvement in general health

[b] Body mass index was available for 172 of 339 participants at the final medical assessment

© 2026 McKetin R et al. *JAMA Psychiatry*.

eTable 5. Model Output for the Primary End Point for the Intention-to-Treat Analysis

Methamphetamine use days	IRR	Std. err.	z	P>z	95% confidence interval	
					Lower bound	Upper bound
Time:						
Week 4	0.83	0.03	-5.42	0	0.77	0.89
Week 8	0.82	0.03	-5.55	0	0.76	0.88
Week 12	0.78	0.03	-6.69	0	0.73	0.84
Condition:						
Mirtazapine	1.05	0.06	0.81	0.417	0.94	1.17
time#condition interaction:						
Week 4#Mirtazapine	0.94	0.05	-1.29	0.198	0.85	1.03
Week 8#Mirtazapine	0.91	0.05	-1.92	0.055	0.82	1.00
Week 12#Mirtazapine	0.89	0.05	-2.3	0.022	0.80	0.98
_constant	0.73	0.03	-7.69	0	0.68	0.79
ln(methden)	1.00	(exposure)				
study_id:						
/lnalpha	-3.21	0.15			-3.50	-2.92

Notes. IRR = Incidence Rate Ratio. St Err. = Standard Error of the IRR.

© 2026 McKetin R et al. *JAMA Psychiatry*.

eTable 6. Estimated Mean (95% CI) Days of Methamphetamine Use Extracted From the Intention-to-Treat Analysis

	Mean	95% confidence interval	
		Low	High
Baseline#Placebo	22.0	20.3	23.8
Baseline#Mirtazapine	23.1	21.3	24.9
Week 4#Placebo	18.2	16.7	19.7
Week 4#Mirtazapine	17.9	16.5	19.4
Week 8#Placebo	18.1	16.5	19.6
Week 8#Mirtazapine	17.2	15.7	18.6
Week 12#Placebo	17.2	15.8	18.7
Week 12#Mirtazapine	16.1	14.7	17.4

Note. Confidence interval calculated using the Delta method.

© 2026 McKetin R et al. *JAMA Psychiatry*.

eTable 7. Model Output for the Primary End Point for the Intention-to-Treat Analysis With Imputed Missing Data

Methamphetamine use days	Coefficient	Std. err.	t	P>t	95% confidence interval	
					Lower bound	Upper bound
Time:						
Week 4	-0.19	0.03	-5.6	0	-0.26	-0.13
Week 8	-0.20	0.04	-5.5	0	-0.27	-0.13
Week 12	-0.25	0.04	-6.68	0	-0.32	-0.17
1.condition (Mirtazapine)	0.05	0.06	0.82	0.413	-0.06	0.16
Time#condition interaction:						
Week 4#1	-0.06	0.05	-1.25	0.213	-0.16	0.03
Week 8#1	-0.09	0.05	-1.79	0.073	-0.19	0.01
Week 12#1	-0.12	0.05	-2.33	0.020	-0.22	-0.02
_constant	-0.31	0.04	-7.7	0	-0.39	-0.23
ln(methden)	1.00	(exposure)				
/lnalpha	-3.19	0.15			-3.49	-2.89
study_id:						
var(_cons[study_id])	0.18	0.02			0.15	0.21

Notes. IRR = Incidence Rate Ratio. St Err. = Standard Error of the IRR.

© 2026 McKetin R et al. *JAMA Psychiatry*.

eTable 8. Model Output for the Primary End Point for the Intention-to-Treat Analysis With Adjustment for Baseline Covariates

methdays	IRR	Std. err.	z	P>z	95% confidence interval	
					Lower	Upper
time						
Week 4	0.83	0.03	-5.4	0	0.78	0.89
Week 8	0.83	0.03	-5.41	0	0.77	0.89
Week 12	0.79	0.03	-6.57	0	0.74	0.85
condition						
Mirtazapine	1.01	0.05	0.29	0.77	0.93	1.11
time#condition						
Week 4#Mirtazapine	0.94	0.05	-1.25	0.21	0.86	1.03
Week 8#Mirtazapine	0.91	0.04	-1.97	0.049	0.82	1.00
Week 12#Mirtazapine	0.89	0.04	-2.38	0.017	0.80	0.98
Covariates:						
Methamphetamine use days at eligibility	1.05	0.00	15.14	0	1.04	1.06
PHQ-9 (depression) score at eligibility	0.99	0.00	-2.78	0.005	0.99	1.00
Injecting main route of methamphetamine administration	0.92	0.04	-2.17	0.03	0.85	0.99
Age	1.00	0.00	-1.22	0.223	0.99	1.00
Sex	0.96	0.04	-1.14	0.256	0.88	1.03
_constant	0.33	0.04	-8.45	0	0.26	0.43
ln(methden)	1.00	(exposure)				
/lnalpha	-3.27	0.15			-3.57	-2.97
study_id:						
var(_cons)	0.09	0.01			0.07	0.11

Notes. IRR = Incidence Rate Ratio. St Err. = Standard Error of the IRR.

© 2026 McKetin R et al. *JAMA Psychiatry*.

eTable 9. Estimated Mean (95% CI) Days of Methamphetamine Use Extracted From the Intention-to-Treat Analysis Adjusted for Baseline Covariates

		95% confidence interval	
	Mean	Lower	Upper
Baseline#Placebo	22.16	20.72	23.60
Baseline#Mirtazapine	22.45	21.03	23.87
Week 4#Placebo	18.39	17.12	19.65
Week 4#Mirtazapine	17.55	16.39	18.71
Week 8#Placebo	18.31	17.02	19.59
Week 8#Mirtazapine	16.84	15.68	18.00
Week 12#Placebo	17.49	16.24	18.74
Week 12#Mirtazapine	15.73	14.63	16.83

© 2026 McKetin R et al. *JAMA Psychiatry*.

eTable 10. Primary and Secondary End Points for the Intention-to-Treat Estimand for Participants Who Were Male at Birth

	Placebo (n = 106)	Mirtazapine (n = 107)	Treatment estimand (95% CI)
**Primary endpoint**			
Mean change in days of methamphetamine use – (95% CI)	-4.7 (-6.5 – -3.0)	-7.0 (-8.7 – -5.2)	-2.3 (-4.7 – 0.2)
**Secondary endpoints**			
Percent methamphetamine negative oral fluid – (95% CI)	13.4 (7.8 – 19.0)	15.2 (9.5 – 20.9)	1.8 (-6.0 – 9.5)
Mean change in PHQ-9 score – (95% CI)	-2.9 (-3.9 – -1.8)	-2.7 (-3.7 – -1.7)	0.2 (-1.3 – 1.7)
Mean change in AIS insomnia score – (95% CI)	-1.3 (-1.9 – -0.6)	-1.6 (-2.3 – -0.9)	-0.4 (-1.4 – 0.6)
Mean change in HIV risk behaviour score – (95% CI)	-1.0 (-1.6 – -0.4)	-1.3 (-1.9 – -0.7)	-0.3 (-1.1 – 0.6)
Mean change in EQ5D quality of life utility score – (95% CI)	2.1 (-1.4 – 5.7)	1.3 (-2.3 – 4.9)	-0.1 (-5.9 – 4.2)

Notes. Treatment effect: Difference between mirtazapine and placebo

© 2026 McKetin R et al. *JAMA Psychiatry*.

eTable 11. Primary and Secondary End Points for the Intention-to-Treat Estimand for Participants Who Were Female or Other at Birth

	Placebo (n = 61)	Mirtazapine (n = 65)	Treatment estimand (95% CI)	Difference between treatment estimand for male versus female or other at birth (95%CI)
**Primary endpoint**				
Mean change in days of methamphetamine use – (95% CI)	-4.9 (-7.4 – -2.5)	-7.1 (-9.5 – -4.7)	-2.2 (-5.6 – 1.2)	0.1 (-4.1 – 4.3)
**Secondary endpoints**				
Percent methamphetamine negative oral fluid – (95% CI)	9.1 (2.8 - 15.3)	9.8 (3.7 - 15.9)	0.7 (-7.9 - 9.3)	-1.1 (-12.7 – 10.5)
Mean change in PHQ-9 score – (95% CI)	-1.3 (-2.7 – 0.1)	-2.2 (-3.5 – -0.9)	-1.0 (-2.9 – 1.0)	-1.2 (-3.6 – 1.3)
Mean change in AIS insomnia score – (95% CI)	-1.0 (-2.0 – -0.1)	-2.0 (-2.9 – -1.1)	-1.0 (-2.3 – 0.3)	-0.6 (-2.2 - 1.0)
Mean change in HIV risk behaviour score – (95% CI)	-1.3 (-2.1 – -0.5)	-1.7 (-2.4 – -0.9)	-0.4 (-1.5 – 0.8)	-0.1 (-1.5 – 1.3)
Mean change in EQ5D quality of life utility score – (95% CI)	-0.4 (-5.3 – 4.4)	3.6 (-0.9 – 8.1)	4.1 (-2.5 – 10.6)	4.9 (-3.4 – 13.2)

Notes. Treatment effect: Difference between mirtazapine and placebo

© 2026 McKetin R et al. *JAMA Psychiatry*.

eTable 12. Primary and Secondary End Points for the Intention-to-Treat Estimand for Participants Who Were Not Depressed at Eligibility

	Placebo (n = 106)	Mirtazapine (n = 107)	Treatment estimand (95% CI)
**Primary endpoint**			
Mean change in days of methamphetamine use – (95% CI)	-3.7 (-5.6 – -1.8)	-6.5 (-8.6 – -4.5)	-2.8 (-5.7 – -0.0)
**Secondary endpoints**			
Percent methamphetamine negative oral fluid – (95% CI)	9.0 (3.7 – 14.4)	11.3 (5.5 – 17.1)	2.3 (-5.4 – 10.0)
Mean change in PHQ-9 score – (95% CI)	-1.6 (-2.8 – -0.5) -	1.0 (-2.1 – 0.1)	0.6 (-1.0 – 2.2)
Mean change in AIS insomnia score – (95% CI)	-0.9 (-1.6 – -0.1)	-1.0 (-1.7 – -0.2)	-0.1 (-1.2 – 1.0)
Mean change in HIV risk behaviour score – (95% CI)	-0.7 (-1.4 – -0.1)	-1.4 (-2.1 – -0.8)	-0.7 (-1.7 – 0.2)
Mean change in EQ5D quality of life utility score – (95% CI)	0.1 (-3.9 – 4.0)	0.9 (-3.0 – 4.8)	0.8 (-4.8 – 6.3)

Notes. Treatment effect: Difference between mirtazapine and placebo

© 2026 McKetin R et al. *JAMA Psychiatry*.

eTable 13. Primary and Secondary End Points for the Intention-to-Treat Estimand for Participants Who Were Depressed at Baseline

	Placebo (n = 61)	Mirtazapine (n = 65)	Treatment estimand (95% CI)	Difference between treatment estimand for depressed versus not-depressed (95%CI)
**Primary endpoint**				
Mean change in days of methamphetamine use – (95% CI)	-5.9 (-8.0 – -3.9)	-7.4 (-9.3 – -5.5)	-1.4 (-4.2 – 1.4)	1.4 (-2.6 – 5.4)
**Secondary endpoints**				
Percent methamphetamine negative oral fluid – (95% CI)	14.8 (8.2 – 21.4))	15.0 (8.7 – 21.4	0.2 (-8.6 – 9.1)	-2.0 (-13.8 – 9.7)
Mean change in PHQ-9 score – (95% CI)	-3.0 (-4.2 – -1.8)	-4.2 (-5.3 – -3.0)	-1.2 (-2.8 – 0.5)	-1.8 (-4.1 – 0.5)
Mean change in AIS insomnia score – (95% CI)	-1.4 (-2.2 – -0.6)	-2.6 (-3.4 – -1.8)	-1.2 (-2.3 – -0.0)	-1.0 (-2.6 – 0.5)
Mean change in HIV risk behaviour score – (95% CI)	-1.4 (-2.1 – -0.7)	-1.4 (-2.1 – -0.7)	0.1 (-0.9 – 1.0)	0.8 (-0.6 – 2.1)
Mean change in EQ5D quality of life utility score – (95% CI)	2.4 (-1.7 – 6.5)	3.7 (-0.3 – 7.7)	1.3 (-4.5 – 7.0)	0.5 (-7.5 – 8.5)

Notes. Treatment effect: Difference between mirtazapine and placebo

© 2026 McKetin R et al. *JAMA Psychiatry*.

eTable 14. Adverse Events Within Each System Organ Classification (SOC) by Condition (From Baseline to Week 12)

	Condition			
	Placebo (n = 167)	Mirtazapine (n = 172)	P value	Total sample (N = 339)
Blood and lymphatic system disorders 10005329, n (%)	0 (0)	0 (0)	.	0 (0)
Cardiac disorders 10007541, n (%)	1 (0)	1 (0)	0.983	2 (0)
Congenital, familial and genetic disorders 10010331, n (%)	0 (0)	0 (0)	.	0 (0)
Ear and labyrinth disorders 10013993, n (%)	1 (0)	1 (0)	0.983	2 (0)
Endocrine disorders 10014698, n (%)	0 (0)	0 (0)	.	0 (0)
Eye disorders 10015919, n (%)	1 (0)	2 (1)	0.579	3 (1)
Gastrointestinal disorders 10017947, n (%)	26 (16)	22 (13)	0.463	48 (14)
General disorders and administration site conditions 0018065, n (%)	22 (13)	21 (12)	0.790	43 (13)
Hepatobiliary disorders 10019805, n (%)	2 (1)	1 (0)	0.545	3 (1)
Immune system disorders 10021428, n (%)	1 (0)	0 (0)	0.309	1 (0)
Infections and infestations 10021881, n (%)	33 (20)	32 (19)	0.787	65 (19)
Injury, poisoning and procedural complications 10022117, n (%)	21 (13)	24 (14)	0.708	45 (13)
**Investigations 10022891, n (%)**	**8 (5)**	**19 (11)**	**0.033**	**27 (8)**
Metabolism and nutrition disorders 10027433, n (%)	48 (29)	55 (32)	0.517	103 (30)
Musculoskeletal and connective tissue disorders 10028395, n (%)	17 (10)	23 (13)	0.362	40 (12)
Neoplasms benign, malignant and unspecified (incl cysts and polyps) 10029104, n (%)	0 (0)	0 (0)	.	0 (0)
**Nervous system disorders 10029205, n (%)**	**78 (47)**	**111 (65)**	**0.001**	**189 (56)**
Pregnancy, puerperium and perinatal conditions 10036585, n (%)	3 (2)	0 (0)	0.077	3 (1)
Psychiatric disorders 10037175, n (%)	74 (44)	85 (49)	0.346	159 (47)
Renal and urinary disorders 10038359, n (%)	1 (0)	6 (3)	0.061	7 (2)
Reproductive system and breast disorders 10038604, n (%)	6 (4)	5 (3)	0.722	11 (3)
Respiratory, thoracic and mediastinal disorders 10038738, n (%)	23 (14)	26 (15)	0.725	49 (14)
Skin and subcutaneous tissue disorders 10040785, n (%)	15 (9)	15 (9)	0.933	30 (9)
Social circumstances 10041244, n (%)	0 (0)	0 (0)	.	0 (0)
Surgical and medical procedures 10042613, n (%)	1 (0)	0 (0)	0.309	1 (0)
Vascular disorders 10047065, n (%)	3 (2)	3 (2)	0.971	6 (2)
Product issues 10077536, n (%)	0 (0)	0 (0)	.	0 (0)

© 2026 McKetin R et al. *JAMA Psychiatry*.

eTable 15. Adverse Events by MedDRA Diagnosis by Condition

	Condition			
	Placebo (n = 167)	Mirtazapine (n = 172)	P value	Total sample (N = 339)
**Cardiac disorders:**				
Palpitations, n (%)	0 (0)	1 (0)	0.324	1 (0)
Tachycardia, n (%)	1 (0)	0 (0)	0.309	1 (0)
**Ear and labyrinth disorders:**				
Ear congestion, n (%)	0 (0)	1 (0)	0.324	1 (0)
Ear pain, n (%)	1 (0)	0 (0)	0.309	1 (0)
**Eye disorders:**				
Panuveitis, n (%)	1 (0)	0 (0)	0.309	1 (0)
Light sensitivity to eye, n (%)	0 (0)	1 (0)	0.324	1 (0)
Abnormal vision, n (%)	0 (0)	1 (0)	0.324	1 (0)
**Gastrointestinal disorders:**				
Abdominal cramps, n (%)	2 (1)	0 (0)	0.150	2 (0)
Abdominal pain, n (%)	1 (0)	2 (1)	0.579	3 (1)
Acid reflux, n (%)	0 (0)	1 (0)	0.324	1 (0)
Burning tongue, n (%)	1 (0)	0 (0)	0.309	1 (0)
Constipation, n (%)	1 (0)	1 (0)	0.983	2 (0)
Diarrhea, n (%)	5 (3)	1 (0)	0.092	6 (2)
Dry mouth, n (%)	0 (0)	2 (1)	0.162	2 (0)
Heartburn, n (%)	2 (1)	1 (0)	0.545	3 (1)
Abdominal pain lower, n (%)	0 (0)	1 (0)	0.324	1 (0)
Nausea, n (%)	6 (4)	4 (2)	0.491	10 (3)
Pancreatitis, n (%)	1 (0)	1 (0)	0.983	2 (0)
Stomach cramps, n (%)	2 (1)	0 (0)	0.150	2 (0)
Stomach pain, n (%)	1 (0)	0 (0)	0.309	1 (0)
Throat irritation, n (%)	0 (.)	1 (100)	.	1 (100)
Toothache, n (%)	3 (2)	7 (4)	0.216	10 (3)
Vomited, n (%)	4 (2)	1 (0)	0.166	5 (1)

© 2026 McKetin R et al. *JAMA Psychiatry*.

	Condition			
	Placebo (n = 167)	Mirtazapine (n = 172)	P value	Total sample (N = 339)
**General disorders and administration site conditions:**				
Ache NOS, n (%)	0 (0)	1 (0)	0.324	1 (0)
Chest pain, n (%)	3 (2)	1 (0)	0.300	4 (1)
Chills, n (%)	3 (2)	0 (0)	0.077	3 (1)
Discharge, n (%)	1 (0)	0 (0)	0.309	1 (0)
Fatigue, n (%)	7 (4)	8 (5)	0.837	15 (4)
Fingers swollen feeling of, n (%)	0 (0)	1 (0)	0.324	1 (0)
Procedural pain, n (%)	1 (0)	0 (0)	0.309	1 (0)
Hernia, n (%)	0 (0)	1 (0)	0.324	1 (0)
Increased thirst, n (%)	0 (0)	1 (0)	0.324	1 (0)
Oedema limbs, n (%)	1 (0)	0 (0)	0.309	1 (0)
Swelling, n (%)	1 (0)	2 (1)	0.579	3 (1)
Calf swelling, n (%)	1 (0)	0 (0)	0.309	1 (0)
Swelling of feet, n (%)	1 (0)	1 (0)	0.983	2 (0)
Oedema hands, n (%)	1 (0)	0 (0)	0.309	1 (0)
Swelling, n (%)	1 (0)	2 (1)	0.579	3 (1)
Tiredness, n (%)	0 (0)	5 (3)	0.026	5 (1)
Unsteady gait, n (%)	1 (0)	1 (0)	0.983	2 (0)
Weakness, n (%)	1 (0)	0 (0)	0.309	1 (0)
**Hepatobiliary disorders:**				
Biliary colic, n (%)	1 (0)	0 (0)	0.309	1 (0)
Gallstones, n (%)	0 (0)	1 (0)	0.324	1 (0)
Fibrosis liver, n (%)	1 (0)	0 (0)	0.309	1 (0)
**Immune system disorders:**				
Hay fever, n (%)	1 (0)	0 (0)	0.309	1 (0)
**Infections and infestations:**				
Bacterial infection, n (%)	1 (0)	0 (0)	0.309	1 (0)
Ruptured appendix, n (%)	0 (0)	1 (0)	0.324	1 (0)
Cellulitis, n (%)	2 (1)	0 (0)	0.150	2 (0)

© 2026 McKetin R et al. *JAMA Psychiatry*.

	Condition			
	Placebo (n = 167)	Mirtazapine (n = 172)	P value	Total sample (N = 339)
Ear infection, n (%)	2 (1)	3 (2)	0.676	5 (1)
Eye infection, n (%)	2 (1)	0 (0)	0.150	2 (0)
Foot infection, n (%)	0 (0)	1 (0)	0.324	1 (0)
Gastroenteritis, n (%)	3 (2)	1 (0)	0.300	4 (1)
Gonorrhoea, n (%)	1 (0)	1 (0)	0.983	2 (0)
Cold, n (%)	14 (8)	15 (9)	0.912	29 (9)
HIV infection, n (%)	1 (0)	0 (0)	0.309	1 (0)
Infection, n (%)	2 (1)	0 (0)	0.150	2 (0)
Infection, n (%)	2 (1)	0 (0)	0.150	2 (0)
Injection site infection, n (%)	0 (0)	1 (0)	0.324	1 (0)
Skin infection NOS, n (%)	1 (0)	2 (1)	0.579	3 (1)
Laryngitis, n (%)	0 (0)	1 (0)	0.324	1 (0)
Staphylococcus aureus infection, n (%)	0 (0)	1 (0)	0.324	1 (0)
Respiratory syncytial virus infection, n (%)	1 (0)	0 (0)	0.309	1 (0)
Shingles, n (%)	1 (0)	0 (0)	0.309	1 (0)
Sinus infection, n (%)	2 (1)	0 (0)	0.150	2 (0)
Recurring skin boils, n (%)	0 (0)	2 (1)	0.162	2 (0)
Stye, n (%)	0 (0)	1 (0)	0.324	1 (0)
Throat infection, n (%)	1 (0)	0 (0)	0.309	1 (0)
Candidiasis genital, n (%)	1 (0)	1 (0)	0.983	2 (0)
Tonsillitis, n (%)	2 (1)	1 (0)	0.545	3 (1)
Tooth infection, n (%)	0 (0)	2 (1)	0.162	2 (0)
Infection NOS, n (%)	0 (0)	1 (0)	0.324	1 (0)
**Injury, poisoning and procedural complications:**				
Ankle injury, n (%)	0 (0)	1 (0)	0.324	1 (0)
Accident, n (%)	4 (2)	5 (3)	0.769	9 (3)
Broken ankle, n (%)	1 (0)	1 (0)	0.983	2 (0)
Fractured finger, n (%)	1 (0)	2 (1)	0.579	3 (1)
Bruise, n (%)	1 (0)	1 (0)	0.983	2 (0)
Bruising of face, n (%)	1 (0)	0 (0)	0.309	1 (0)
Rib contusion, n (%)	0 (0)	1 (0)	0.324	1 (0)

© 2026 McKetin R et al. *JAMA Psychiatry*.

	Condition			
	Placebo (n = 167)	Mirtazapine (n = 172)	P value	Total sample (N = 339)
Bruising, n (%)	1 (0)	2 (1)	0.579	3 (1)
Burn, n (%)	0 (0)	1 (0)	0.324	1 (0)
Corneal abrasion, n (%)	0 (0)	1 (0)	0.324	1 (0)
Laceration of finger, n (%)	0 (0)	1 (0)	0.324	1 (0)
Dog bite, n (%)	1 (0)	0 (0)	0.309	1 (0)
Accident, n (%)	4 (2)	5 (3)	0.769	9 (3)
Fractured toe, n (%)	0 (0)	1 (0)	0.324	1 (0)
Overdose, n (%)	3 (2)	1 (0)	0.300	4 (1)
Injury, n (%)	5 (3)	1 (0)	0.092	6 (2)
Sting, n (%)	0 (0)	1 (0)	0.324	1 (0)
Knee dislocation, n (%)	1 (0)	0 (0)	0.309	1 (0)
Motor cycling accident, n (%)	0 (0)	1 (0)	0.324	1 (0)
Injury, n (%)	5 (3)	1 (0)	0.092	6 (2)
Rib fracture, n (%)	1 (0)	0 (0)	0.309	1 (0)
Vehicular accident, n (%)	0 (0)	1 (0)	0.324	1 (0)
Accident, n (%)	4 (2)	5 (3)	0.769	9 (3)
Spider bite, n (%)	1 (0)	0 (0)	0.309	1 (0)
Laceration of head, n (%)	0 (0)	1 (0)	0.324	1 (0)
Tooth injury, n (%)	1 (0)	2 (1)	0.579	3 (1)
Head injury, n (%)	0 (0)	1 (0)	0.324	1 (0)
Laceration of hand, n (%)	0 (0)	1 (0)	0.324	1 (0)
**Investigations:**				
Blood in urine, n (%)	0 (0)	1 (0)	0.324	1 (0)
Blood pressure increased, n (%)	3 (2)	0 (0)	0.077	3 (1)
Heart rate increased, n (%)	0 (0)	1 (0)	0.324	1 (0)
**Weight gain, n (%)**	**5 (3)**	**17 (10)**	**0.010**	**22 (6)**
Fluid retention, n (%)	2 (1)	6 (3)	0.165	8 (2)
Fluid retention, n (%)	2 (1)	6 (3)	0.165	8 (2)
Increased appetite, n (%)	45 (27)	48 (28)	0.843	93 (27)
Decreased appetite, n (%)	3 (2)	5 (3)	0.501	8 (2)

© 2026 McKetin R et al. *JAMA Psychiatry*.

	Condition			
	Placebo (n = 167)	Mirtazapine (n = 172)	P value	Total sample (N = 339)
**Musculoskeletal and connective tissue disorders:**				
Knee pain, n (%)	1 (0)	2 (1)	0.579	3 (1)
Arthritis, n (%)	1 (0)	0 (0)	0.309	1 (0)
Back pain, n (%)	4 (2)	6 (3)	0.552	10 (3)
Bucket handle tear of medial meniscus of knee, n (%)	0 (0)	1 (0)	0.324	1 (0)
Groin pain, n (%)	0 (0)	1 (0)	0.324	1 (0)
Pain in hip, n (%)	1 (0)	2 (1)	0.579	3 (1)
Joint pain, n (%)	3 (2)	1 (0)	0.300	4 (1)
Knee injury, n (%)	1 (0)	1 (0)	0.983	2 (0)
Aches & pains in legs, n (%)	0 (0)	1 (0)	0.324	1 (0)
Leg cramps, n (%)	1 (0)	0 (0)	0.309	1 (0)
Leg pain, n (%)	0 (0)	2 (1)	0.162	2 (0)
Muscle ache, n (%)	1 (0)	0 (0)	0.309	1 (0)
Muscle cramps, n (%)	0 (0)	1 (0)	0.324	1 (0)
Back muscle spasms, n (%)	1 (0)	0 (0)	0.309	1 (0)
Low back pain, n (%)	2 (1)	0 (0)	0.150	2 (0)
Pain in hip, n (%)	1 (0)	2 (1)	0.579	3 (1)
Knee pain, n (%)	1 (0)	2 (1)	0.579	3 (1)
Shoulder bursitis, n (%)	0 (0)	1 (0)	0.324	1 (0)
Shoulder pain, n (%)	1 (0)	2 (1)	0.579	3 (1)
Pain in arm, n (%)	0 (0)	2 (1)	0.162	2 (0)
Pain in hip, n (%)	1 (0)	2 (1)	0.579	3 (1)
Ankles swelling, n (%)	0 (0)	1 (0)	0.324	1 (0)
Hip bursitis, n (%)	1 (0)	0 (0)	0.309	1 (0)
**Nervous system disorders:**				
Amnesia, n (%)	0 (0)	1 (0)	0.324	1 (0)
Brain fog, n (%)	4 (2)	7 (4)	0.384	11 (3)
Taste changed, n (%)	0 (0)	1 (0)	0.324	1 (0)
Confusion, n (%)	0 (0)	5 (3)	0.026	5 (1)

© 2026 McKetin R et al. *JAMA Psychiatry*.

	Condition			
	Placebo (n = 167)	Mirtazapine (n = 172)	P value	Total sample (N = 339)
Dizziness, n (%)	6 (4)	9 (5)	0.463	15 (4)
**Drowsiness, n (%)**	**55 (33)**	**80 (47)**	**0.011**	**135 (40)**
Felt faint, n (%)	0 (0)	1 (0)	0.324	1 (0)
Forgetfulness, n (%)	0 (0)	2 (1)	0.162	2 (0)
Groggy on awakening, n (%)	3 (2)	1 (0)	0.300	4 (1)
Headache, n (%)	23 (14)	21 (12)	0.669	44 (13)
Headaches, n (%)	4 (2)	3 (2)	0.673	7 (2)
Increased need for sleep, n (%)	0 (0)	1 (0)	0.324	1 (0)
Lethargy, n (%)	4 (2)	6 (3)	0.552	10 (3)
Light-headed, n (%)	1 (0)	2 (1)	0.579	3 (1)
Microsleep, n (%)	1 (0)	0 (0)	0.309	1 (0)
Migraine, n (%)	3 (2)	6 (3)	0.333	9 (3)
Nerve pain, n (%)	1 (0)	0 (0)	0.309	1 (0)
Pins and needles, n (%)	0 (0)	1 (0)	0.324	1 (0)
Restless arms, n (%)	1 (0)	0 (0)	0.309	1 (0)
Sedation, n (%)	0 (0)	3 (2)	0.086	3 (1)
Blackout, n (%)	0 (0)	1 (0)	0.324	1 (0)
Coordination abnormal, n (%)	1 (0)	0 (0)	0.309	1 (0)
Seizure, n (%)	0 (0)	1 (0)	0.324	1 (0)
**Pregnancy, puerperium and perinatal conditions:**				
Pregnancy, n (%)	2 (1)	0 (0)	0.150	2 (0)
**Psychiatric disorders:**				
Aggression, n (%)	0 (0)	1 (0)	0.324	1 (0)
Anxiety, n (%)	0 (0)	4 (2)	0.047	4 (1)
Exacerbation of anxiety, n (%)	7 (4)	7 (4)	0.955	14 (4)
Auditory hallucinations, n (%)	1 (0)	1 (0)	0.983	2 (0)
Nervous, n (%)	0 (0)	1 (0)	0.324	1 (0)
Depression, n (%)	5 (3)	2 (1)	0.236	7 (2)
Depression NOS, n (%)	0 (0)	1 (0)	0.324	1 (0)
Dissociation, n (%)	0 (0)	2 (1)	0.162	2 (0)
Stress, n (%)	3 (2)	5 (3)	0.501	8 (2)

© 2026 McKetin R et al. *JAMA Psychiatry*.

	Condition			
	Placebo (n = 167)	Mirtazapine (n = 172)	P value	Total sample (N = 339)
Drug-induced psychosis, n (%)	0 (0)	1 (0)	0.324	1 (0)
Flat affect, n (%)	0 (0)	3 (2)	0.086	3 (1)
Increased agitation, n (%)	1 (0)	2 (1)	0.579	3 (1)
Anger, n (%)	1 (0)	0 (0)	0.309	1 (0)
Gambling addiction, n (%)	0 (0)	1 (0)	0.324	1 (0)
Drug use disorder, n (%)	5 (3)	6 (3)	0.797	11 (3)
Insomnia, n (%)	3 (2)	1 (0)	0.300	4 (1)
Irritability, n (%)	2 (1)	3 (2)	0.676	5 (1)
Irritable, n (%)	9 (5)	13 (8)	0.418	22 (6)
Lack of motivation, n (%)	2 (1)	3 (2)	0.676	5 (1)
Lack of motivation, n (%)	2 (1)	3 (2)	0.676	5 (1)
Low mood, n (%)	20 (12)	24 (14)	0.588	44 (13)
Sexual desire decreased, n (%)	2 (1)	1 (0)	0.545	3 (1)
Active suicidal ideation, n (%)	2 (1)	1 (0)	0.545	3 (1)
Mood alteration NOS, n (%)	1 (0)	0 (0)	0.309	1 (0)
Mood swings, n (%)	4 (2)	2 (1)	0.390	6 (2)
Negative thoughts, n (%)	1 (0)	0 (0)	0.309	1 (0)
Nightmare, n (%)	5 (3)	1 (0)	0.092	6 (2)
Panic attacks, n (%)	0 (0)	2 (1)	0.162	2 (0)
Paranoia, n (%)	1 (0)	1 (0)	0.983	2 (0)
Psychotic episode, n (%)	0 (0)	0 (0)	.	0 (0)
Restless, n (%)	1 (0)	1 (0)	0.983	2 (0)
Low mood, n (%)	20 (12)	24 (14)	0.588	44 (13)
Sleep disturbance, n (%)	2 (1)	1 (0)	0.545	3 (1)
Difficulty sleeping, n (%)	5 (3)	5 (3)	0.962	10 (3)
Sleep talking, n (%)	0 (0)	1 (0)	0.324	1 (0)
Bizarre dreams, n (%)	0 (0)	1 (0)	0.324	1 (0)
Suicidal ideation, n (%)	11 (7)	11 (6)	0.943	22 (6)
Suicide attempt, n (%)	1 (0)	3 (2)	0.329	4 (1)
Visual hallucinations, n (%)	1 (0)	2 (1)	0.579	3 (1)
Vivid dreams, n (%)	16 (10)	21 (12)	0.438	37 (11)

© 2026 McKetin R et al. *JAMA Psychiatry*.

	Condition			
	Placebo (n = 167)	Mirtazapine (n = 172)	P value	Total sample (N = 339)
**Renal and urinary disorders:**				
Bladder infection, n (%)	0 (0)	1 (0)	0.324	1 (0)
Urination frequency of, n (%)	0 (0)	2 (1)	0.162	2 (0)
Kidney pain, n (%)	0 (0)	1 (0)	0.324	1 (0)
Bladder pain syndrome, n (%)	0 (0)	1 (0)	0.324	1 (0)
**Reproductive system and breast disorders:**				
Breast lump, n (%)	0 (0)	1 (0)	0.324	1 (0)
Ejaculation failure, n (%)	1 (0)	0 (0)	0.309	1 (0)
Erectile dysfunction, n (%)	2 (1)	0 (0)	0.150	2 (0)
Menstruation delayed, n (%)	1 (0)	0 (0)	0.309	1 (0)
Heavy menstrual bleeding, n (%)	1 (0)	1 (0)	0.983	2 (0)
Testicle adenoma, n (%)	1 (0)	0 (0)	0.309	1 (0)
Spotting menstrual, n (%)	0 (0)	2 (1)	0.162	2 (0)
Vaginal discharge, n (%)	0 (0)	1 (0)	0.324	1 (0)
**Respiratory, thoracic and mediastinal disorders:**				
Asthma, n (%)	0 (0)	1 (0)	0.324	1 (0)
Asthmatic attack, n (%)	1 (0)	0 (0)	0.309	1 (0)
Chest infection, n (%)	1 (0)	4 (2)	0.187	5 (1)
Cough, n (%)	2 (1)	5 (3)	0.269	7 (2)
COVID-19, n (%)	7 (4)	8 (5)	0.837	15 (4)
Difficulty breathing, n (%)	1 (0)	0 (0)	0.309	1 (0)
Influenza, n (%)	8 (5)	5 (3)	0.367	13 (4)
Nosebleed, n (%)	1 (0)	0 (0)	0.309	1 (0)
Pneumonia, n (%)	0 (0)	1 (0)	0.324	1 (0)
Runny nose, n (%)	1 (0)	3 (2)	0.329	4 (1)
Shortness of breath, n (%)	0 (0)	1 (0)	0.324	1 (0)
Sore throat, n (%)	1 (0)	2 (1)	0.579	3 (1)
Wheezing, n (%)	1 (0)	0 (0)	0.309	1 (0)
**Skin and subcutaneous tissue disorders:**				
Eczema, n (%)	0 (0)	1 (0)	0.324	1 (0)
Skin breakout, n (%)	1 (0)	0 (0)	0.309	1 (0)

© 2026 McKetin R et al. *JAMA Psychiatry*.

	Condition			
	Placebo (n = 167)	Mirtazapine (n = 172)	P value	Total sample (N = 339)
Foot callus, n (%)	1 (0)	0 (0)	0.309	1 (0)
Sweating increased, n (%)	2 (1)	0 (0)	0.150	2 (0)
Itch, n (%)	3 (2)	2 (1)	0.629	5 (1)
Pimples, n (%)	1 (0)	0 (0)	0.309	1 (0)
Dry skin, n (%)	1 (0)	1 (0)	0.983	2 (0)
Skin irritation, n (%)	0 (0)	2 (1)	0.162	2 (0)
Rash, n (%)	6 (4)	8 (5)	0.624	14 (4)
Skin lesion, n (%)	1 (0)	1 (0)	0.983	2 (0)
Sweating, n (%)	2 (1)	1 (0)	0.545	3 (1)
**Surgical and medical procedures:**				
Wisdom teeth removal, n (%)	1 (0)	0 (0)	0.309	1 (0)
**Vascular disorders:**				
Hot flush, n (%)	1 (0)	3 (2)	0.329	4 (1)
Peripheral artery occlusion, n (%)	1 (0)	0 (0)	0.309	1 (0)
Numbness, n (%)	1 (0)	0 (0)	0.309	1 (0)

© 2026 McKetin R et al. *JAMA Psychiatry*.

eTable 16. Serious Adverse Events by Condition

Condition	Description	Relatedness*
Placebo:	Biliary colic	Possibly related
	Preterm labour	Possibly related
	GHB overdose	Possibly related
	Suicide attempt (intentional GHB overdose)	Possibly related
	Pancreatitis	Possibly related
	Panuveitis	Unrelated
	Blackout / suspected overdose	Possibly related
	Pregnancy-related hospitalisation	Unrelated
	Cellulitis-related hospitalisation	Unrelated
	Injury-related hospitalisation from assault	Unrelated
	Peripheral artery occlusion	Unrelated
Mirtazapine:	Broken ankle	Unrelated
	Suicide attempt	Unrelated
	Pancreatitis	Unrelated
	Suicide attempt (intentional overdose)	Possibly related
	Ruptured appendix	Unrelated
	Motor cycling accident	Unrelated
	Suicide attempt	Possibly related

* SAEs were only regarded as related to the medication if they were graded as probably related or definitely related. Relatedness of an AE to the medication was graded as unrelated, possibly related, probably related or definitely related.

© 2026 McKetin R et al. *JAMA Psychiatry*.

eReferences.

1. NCCIH Clinical Research Toolbox. National Institute for Health, National Centre for Complementary and Integrative Health. July 24 2018 (https://nccih.nih.gov/grants/toolbox).
2. Herdman M, Gudex C, Lloyd A, et al. Development and preliminary testing of the new five-level version of EQ-5D (EQ-5D-5L). Quality of life research : an international journal of quality of life aspects of treatment, care and rehabilitation 2011;20(10):1727-36. (In eng). DOI: 10.1007/s11136-011-9903-x.
3. Reilly MC, Zbrozek AS, Dukes EM. The validity and reproducibility of a work productivity and activity impairment instrument. PharmacoEconomics 1993;4(5):353-65. (In eng). DOI: 10.2165/00019053-199304050-00006.
4. Mohebbi M, Dodd S, Dean OM, Berk M. Patient centric measures for a patient centric era: Agreement and convergent between ratings on The Patient Global Impression of Improvement (PGI-I) scale and the Clinical Global Impressions - Improvement (CGI-S) scale in bipolar and major depressive disorder. European psychiatry : the journal of the Association of European Psychiatrists 2018;53:17-22. (In eng). DOI: 10.1016/j.eurpsy.2018.05.006.
5. Atkinson MJ, Sinha A, Hass SL, et al. Validation of a general measure of treatment satisfaction, the Treatment Satisfaction Questionnaire for Medication (TSQM), using a national panel study of chronic disease. Health and Quality of Life Outcomes 2004;2:12-12. DOI: 10.1186/1477-7525-2-12.
6. Lee KJ, Tilling KM, Cornish RP, et al. Framework for the treatment and reporting of missing data in observational studies: The Treatment And Reporting of Missing data in Observational Studies framework. Journal of Clinical Epidemiology 2021;134:79-88. DOI: 10.1016/j.jclinepi.2021.01.008.
7. van Buuren S, Groothuis-Oudshoorn K. mice: multivariate imputation by chained equations in R. Journal of Statistical Software 2011;45(3). DOI: 10.18637/jss.v045.i03.
8. Wright MN, Wager S, Probst P. Ranger: A fast implementation of random forests. R package version 012 2020;1.
9. Graham JW, Olchowski AE, Gilreath TD. How many imputations are really needed? Some practical clarifications of multiple imputation theory. Prevention Science 2007;8(3):206-213. (journal article). DOI: 10.1007/s11121-007-0070-9.

© 2026 McKetin R et al. *JAMA Psychiatry*.
