# Supplementary material for: Mirtazapine for Methamphetamine Use Disorder: A Randomized Clinical Trial
Source: JAMA Psychiatry. 2026 Apr 1;83(6):581–9. doi: 10.1001/jamapsychiatry.2026.0159 (PMC13044789; doi:10.1001/jamapsychiatry.2026.0159)
Supplement: Supplement 4. — Data Sharing Statement [file jamapsychiatry-e260159-s004.pdf]

Data Sharing Statement

McKetin. Mirtazapine for Methamphetamine Use Disorder. *JAMA Psychiatry*. Published April 01, 2026. doi:10.1001/jamapsychiatry.2026.0159

Data
Additional Information: Tina Trial. Australian and New Zealand Clinical Trials Registry https://anzctr.org.au/ ACTRN12622000235707
Data available: Yes
Data types: Deidentified participant data, Data dictionary
How to access data: Requests to be made to Rebecca McKetin, r.mcketin@unsw.edu.au
When available: beginning date: 04-01-2027

Supporting Documents
Document types: Other (please specify)
Additional Information: Codebook and protocol
How to access documents: r.mcketin@unsw.edu.au
When available: beginning date: 04-01-2027

Additional Information
Who can access the data: Research whose proposed use of the data has received ethics approval and approval of the study investigators.
Types of analyses: Approved research.
Mechanisms of data availability: After approval of proposal, with investigator support, and with a signed data access agreement.
Any additional restrictions: Acknowledgement of funding source and study team in all publications arising from the data. Data security requirements and ethics approvals. Data provided may be restricted or aggregated to maintain participant confidentiality.
